# Global, regional, and national burden of ischemic heart disease in youths and young adults aged 15–39 years in 204 countries/territories, 1990–2021: a systematic analysis of global burden of disease study 2021

**DOI:** 10.3389/fcvm.2025.1649408

**Published:** 2025-10-14

**Authors:** Weixin Sun, Peijie Li, Qimeng Ni, Renyou Pan, Tingting Gu, Xiaolong Song, Ping Liu, Yuexing Gu

**Affiliations:** ^1^ Department of Cardiology, Yancheng TCM Hospital Affiliated to Nanjing University of Chinese Medicine, Yancheng, China; ^2^ Department of Cardiology, Yancheng TCM Hospital, Yancheng, China

**Keywords:** ischemic heart disease, youths and young adults, global burden of disease, risk factor, prediction

## Abstract

**Background:**

Ischemic heart disease (IHD) remains a global public health challenge. This study explores global trends in IHD burden among youths and young adults aged 15–39 years from 1990 to 2021.

**Methods:**

Data were obtained from the 2021 Global Burden of Disease (GBD) study. Estimated annual percentage change was used to assess trends in age-standardized prevalence rate (ASPR), incidence rate (ASIR), mortality rate (ASMR), and disability-adjusted life years (DALYs). Risk factors were analyzed globally and by socio-demographic index (SDI) regions. Bayesian age-period-cohort models predicted trends over the next 30 years.

**Results:**

From 1990 to 2021, IHD-related mortality and DALYs declined overall, while prevalence and incidence increased. The largest increases in ASPR, ASIR, ASMR, and DALY rates were observed in middle-SDI regions. Geographically, Asia bore the heaviest burden, whereas high-income North America showed the greatest decreases in prevalence and incidence. In 2021, Oceania had the highest IHD-related deaths and DALYs, with Lesotho exhibiting the greatest rise in ASMR and DALY rates. The IHD burden rose with age, peaking in the 35–39 years group, and was higher in males. Major risk factors included high low-density lipoprotein cholesterol, smoking, and high systolic blood pressure. Projections suggest a global decline in IHD burden, with decreasing incidence and deaths across both sexes by 2050.

**Conclusions:**

While mortality and DALYs have decreased over the past 30 years, prevalence and incidence of IHD in youths and young adults have increased. The burden is projected to decline, emphasizing the need for targeted interventions, particularly in males aged 35–39 years, based on regional epidemiological patterns and risk factors.

## 1 Introduction

Ischemic heart disease (IHD), also known as coronary heart disease, is one of the leading causes of death and disability worldwide (1–4). On an annual basis, deaths caused by IHD account for about 13.2% of all deaths globally (5). Recent epidemiological study shows that, in 2021, the number of people suffering from IHD worldwide reaches 31.87 million (6). And the number of deaths related to IHD globally has reached 8.99 million (6). Emerging evidence highlights that a multitude of factors contribute to IHD. Notably, a recent investigation identified high systolic blood pressure (54.6%), high low-density lipoprotein cholesterol (46.6%), and smoking (23.9%) as the three predominant contributors (4). IHD is typically more prevalent in middle-aged and elderly populations, with its incidence increasing with age (1). However, recent evidence demonstrates that IHD imposes a substantial and often overlooked burden on adolescents and young adults aged 15–39 years (7). This trend has attracted global attention, as the early onset of IHD may have significant long-term health implications for individuals and lead to a higher social and economic burden. Therefore, understanding the temporal trends and attributable risk factors of IHD in adolescents and young adults is essential to elucidate its evolving epidemiology and to inform targeted primary prevention and early intervention strategies for this vulnerable population. Consequently, a comprehensive and up-to-date assessment of the IHD burden specifically in the 15–39 age group remains limited.

In recent years, the establishment of the Global Burden of Disease (GBD) database has provided essential data foundation for analyzing the temporal trends of IHD across different dimensions (7, 8). The GBD 2017 study indicates that IHD affects approximately 126 million people globally, accounting for about 1.72% of the world's population (9). According to GBD 2019 study, 59 countries were identified where IHD was the dominant cause of mortality (10). Data from GBD 2021 indicate that high systolic blood pressure caused the greatest IHD burden, followed by high low-density lipoprotein (11). As a common cardiovascular disease, the global age-standardized incidence rate (ASIR) of IHD among youths and young adults is elevated from 126.8 per 100,000 persons in 1990 to 129.85 per 100,000 persons in 2019 (7). The increase of IHD burden among youths and young adults is linked with various factors, including smoking, high systolic blood pressure (SBP), high body mass index, and unhealthy lifestyle choices (7, 12). Currently, the global trend of IHD burden among youths and young adults based on GBD 2021 data remains uncertain, and this trend may undergo significant changes due to the global COVID-19 pandemic.

In present study, the epidemiological trends of IHD in youths and young adults aged at 15–39 years are explored using the GBD 2021 database. The objective is to provide an updated assessment in prevalence, incidence, mortality, and disability-adjusted life years (DALYs) of IHD in youths and young adults from 1990 to 2021. We also aim to explore the changes in the main attributable risk factors and future trends for IHD, with a particular focus on region and gender differences. This analysis will provide a critical evidence base for developing targeted public health strategies. Investigating the global burden of IHD not only deepens our understanding of its epidemiological patterns but also provides key scientific evidence to guide the formulation of health policies and the optimal allocation of medical resources.

## 2 Methods

### 2.1 Data acquirement

The IHD-related data from 1990 to 2021 were downloaded from the GBD database (http://ghdx.healthdata.org), including prevalence, incidence, mortality, DALYs, and their corresponding age-standardized rates (ASRs). GBD database provided IHD data at global, regional and national levels, covering 5 socio-demographic index (SDI) regions, 4 continents, 6 World Health Organization (WHO) regions, and 21 GBD super-regions. The 204 countries and territories were grouped into five levels, including low, low-middle, middle, high-middle, and high SDI regions (13). This study focused on youths (15–19 years) and young adults (20–39 years) with IHD as per earlier publication (7). The age was grouped into 5 subgroups: 15–19 years, 20–24 years, 25–29 years, 30–34 years, and 35–39 years age group. The reference year for the analysis of IHD risk factors was 2021. Ethical approval and informed consent were not required as the GBD data were publicly accessible and the analyses did not involve any identifiable information.

### 2.2 Statistical analysis

The ASR tendencies of IHD in prevalence (ASPR), incidence, mortality (ASMR), and DALYs were evaluated using the estimated annual percentage change (EAPC), describing with 95% confidence intervals (CI). The EAPC was derived from a regression model that represented the change pattern of ASRs during a designated time period. EAPC was calculated with the formula of 100 × [exp (*β*) − 1]. EAPC > 0: the increase of ASRs. EAPC = 0: the stable of ASRs. EAPC < 0: the decrease of ASRs (14). Furthermore, the ASR indicators were exhibited along with their 95% uncertainty interval (UI). The future burden tendencies of IHD for males and females were forecasted using the Bayesian age-period-cohort (BAPC) model over the next 30 years. R software (version 4.4.1) was employed for the statistical analyses and visualizations.

## 3 Results

### 3.1 Global level of IHD

**Figure 1:**
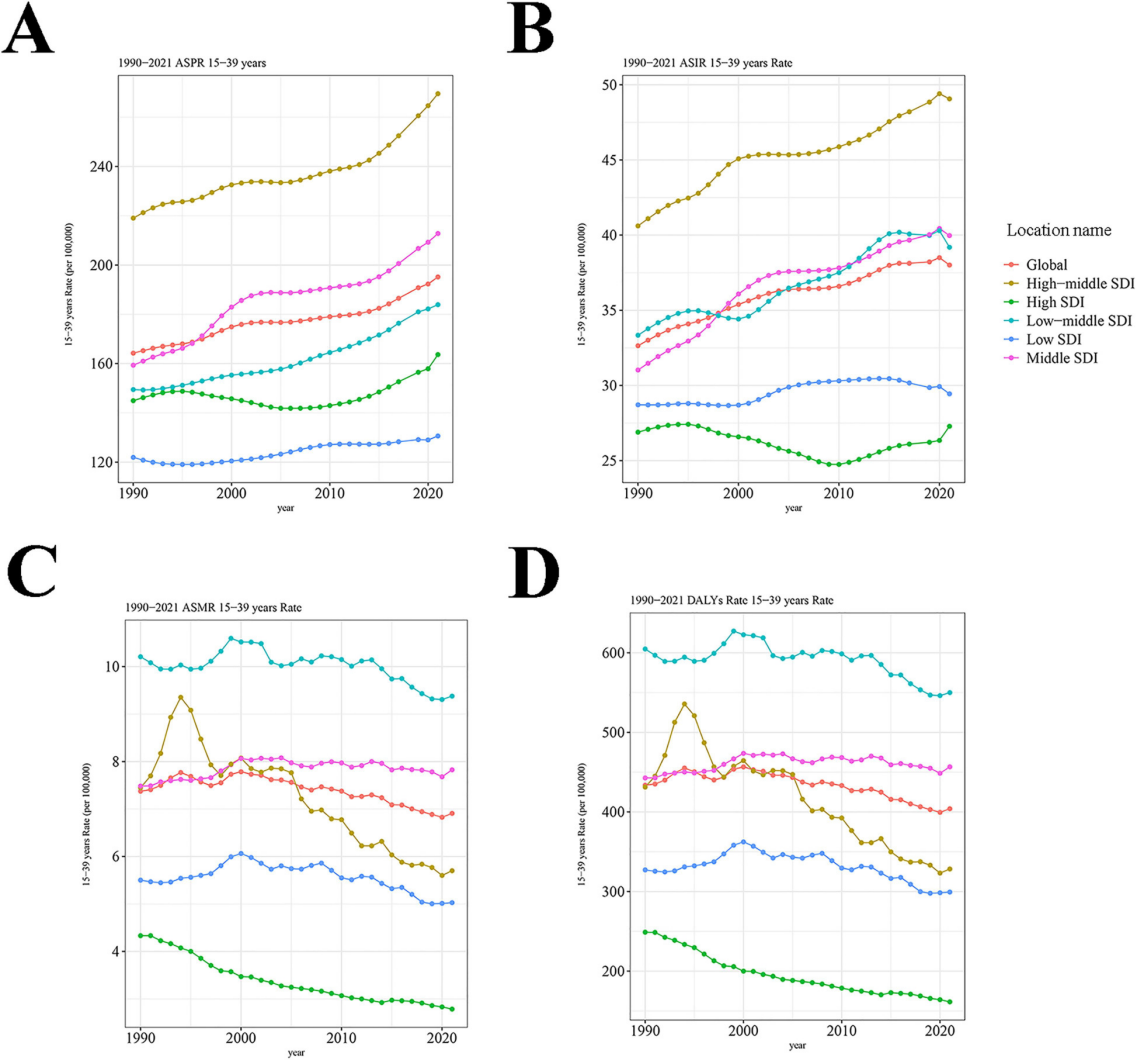
Trends in IHD prevalence, incidence, mortality and DALYs from 1990 to 2021. **(A)** The ASPR of IHD. **(B)** The ASIR of IHD. **(C)** The ASMR of IHD. **(D)** The DALYs of IHD. IHD, ischemic heart disease; DALYs, disability-adjusted life years; ASPR, age-standardized prevalence rate; ASIR, age-standardized incidence rate; ASMR, age-standardized mortality rate; SDI, socio-demographic index.

In 2021, the global burden of IHD-related prevalence remained massive, with a total of 5,805,593 cases (95% UI: 4,618,891–7,250,491). And the ASPR exhibited an upward trend from 164.242 per 100,000 persons (95% UI: 133.026–198.89) in 1990 to 195.157 per 100,000 persons (95% UI: 155.266–243.728) in 2021, with an EAPC of 0.46 (95% CI: 0.42–0.5) (Table 1 and Figure 1A). The global incidence of IHD in 2021 reached 1,130,725 cases (95% UI: 699,328–1,611,703), marking an increase from 1990. The upward trend of ASIR was observed over the study period, with an EAPC of 0.5 (95% CI: 0.46–0.54) (Table 1 and Figure 1B). In contrast to 1990, the global mortality of IHD was reduced in 2021, with an ASMR of 6.908 per 100,000 persons (95% UI: 6.485–7.308) and an EAPC of −0.33 (95% CI: −0.42 to −0.25) (Table 1 and Figure 1C). A similar tendency was found in DALYs, with an EAPC of −0.34 (95% CI: −0.42 to −0.25) (Table 1 and Figure 1D).

**Table 1:** Global and regional trends in IHD burden: prevalence, incidence, deaths and disability-adjusted life years (1990–2021).

Location name	1990		2021		EAPC (95% CI)
	Number	ASR (95% UI)	Number	ASR (95% UI)	
Prevalence					
Global	3,599,844 (2,915,660–4,359,274)	164.242 (133.026–198.89)	5,805,593 (4,618,891–7,250,491)	195.157 (155.266–243.728)	0.46 (0.42 to 0.5)
SDI regions					
High-middle SDI	991,290 (796,647–1,213,623)	219.051 (176.04–268.182)	1,186,714 (931,746–1,505,715)	269.544 (211.632–342)	0.51 (0.44 to 0.58)
High SDI	502,894 (410,455–605,909)	144.941 (118.299–174.631)	578,031 (460,406–708,874)	163.636 (130.338–200.677)	0.14 (0.02 to 0.27)
Low-middle SDI	677,547 (551,660–818,545)	149.437 (121.672–180.535)	1,475,824 (1,177,048–1,836,751)	183.905 (146.674–228.881)	0.66 (0.61 to 0.72)
Low SDI	224,712 (183,166–268,125)	121.921 (99.38–145.476)	586,496 (463,993–721,902)	130.605 (103.325–160.758)	0.31 (0.27 to 0.35)
Middle SDI	1,199,247 (959,440–1,480,081)	159.34 (127.478–196.654)	1,973,547 (1,548,563–2,505,101)	212.784 (166.963–270.095)	0.82 (0.74 to 0.91)
Incidence					
Global	715,468 (439,522–1,007,224)	32.643 (20.053–45.954)	1,130,725 (699,328–1,611,703)	38.01 (23.508–54.178)	0.5 (0.46 to 0.54)
SDI regions					
High-middle SDI	183,782 (112,813–257,748)	40.612 (24.929–56.956)	216,005 (132,862–306,370)	49.062 (30.177–69.587)	0.55 (0.5 to 0.6)
High SDI	93,303 (55,396–135,710)	26.891 (15.966–39.114)	96,378 (58,563–138,985)	27.284 (16.579–39.346)	−0.2 (−0.31 to −0.1)
Low-middle SDI	151,169 (92,506–214,405)	33.341 (20.403–47.288)	314,519 (195,166–447,758)	39.193 (24.32–55.796)	0.64 (0.57 to 0.7)
Low SDI	52,918 (32,230–75,603)	28.712 (17.487–41.02)	132,208 (80,738–188,271)	29.441 (17.979–41.925)	0.22 (0.16 to 0.27)
Middle SDI	233,540 (144,103–327,790)	31.03 (19.147–43.552)	370,736 (228,162–530,652)	39.972 (24.6–57.214)	0.81 (0.72 to 0.91)
Deaths					
Global	161,659 (152,907–170,934)	7.376 (6.976–7.799)	205,513 (192,905–217,407)	6.908 (6.485–7.308)	−0.33 (−0.42 to −0.25)
SDI regions					
High-middle SDI	33,703 (31,589–35,712)	7.448 (6.98–7.891)	25,097 (23,144–27,701)	5.7 (5.257–6.292)	−1.46 (−1.68 to −1.24)
High SDI	15,033 (14,651–15,434)	4.333 (4.223–4.448)	9,844 (8,870–11,090)	2.787 (2.511–3.14)	−1.4 (−1.52 to −1.28)
Low-middle SDI	46,283 (41,949–51,387)	10.208 (9.252–11.334)	75,254 (69,087–81,123)	9.378 (8.609–10.109)	−0.22 (−0.32 to −0.12)
Low SDI	10,141 (8,582–11,950)	5.502 (4.656–6.484)	22,578 (20,229–25,336)	5.028 (4.505–5.642)	−0.31 (−0.47 to −0.14)
Middle SDI	56,323 (53,372–59,144)	7.483 (7.091–7.858)	72,585 (67,247–78,040)	7.826 (7.25–8.414)	0.11 (0.03 to 0.18)
Disability-adjusted life years					
Global	9,503,087 (8,981,809–10,067,389)	433.575 (409.792–459.321)	12,018,306 (11,291,576–12,723,391)	404 (379.571–427.702)	−0.34 (−0.42 to −0.25)
SDI regions					
High-middle SDI	1,951,719 (1,824,009–2,068,539)	431.283 (403.063–457.098)	1,445,440 (1,333,557–1,591,354)	328.31 (302.897–361.452)	−1.43 (−1.64 to −1.22)
High SDI	863,527 (840,174–886,213)	248.88 (242.15–255.419)	570,050 (513,056–638,898)	161.377 (145.242–180.868)	−1.35 (−1.47 to −1.24)
Low-middle SDI	2,742,028 (2,485,785–3,044,705)	604.77 (548.254–671.527)	4,413,593 (4,055,356–4,751,956)	549.985 (505.345–592.15)	−0.26 (−0.36 to −0.16)
Low SDI	602,944 (508,484–710,635)	327.139 (275.887–385.568)	1,344,071 (1,205,343–1,506,721)	299.306 (268.414–335.526)	−0.32 (−0.49 to −0.16)
Middle SDI	3,332,688 (3,154,069–3,496,999)	442.804 (419.072–464.636)	4,236,155 (3,938,228–4,552,079)	456.733 (424.611–490.795)	0.07 (0 to 0.15)

IHD, ischemic heart disease; SDI, socio-demographic index; ASR, age-standardized rate; EAPC, estimated annual percentage change; UI, uncertainty interval; CI, confidence interval.

### 3.2 Regional level of IHD

The disease burden of IHD displayed obvious regional differences and was closely related to SDI levels. In 2021, the burdens of IHD-related prevalence and incidence were the highest in the high-middle SDI regions, with an ASPR of 269.544 per 100,000 persons (95% UI: 211.632–342) and an ASIR of 49.062 per 100,000 persons (95% UI: 30.177–69.587). From 1990 to 2021, middle SDI regions experienced the largest increases in both ASPR and ASIR, with an EAPC of 0.82 (95% CI: 0.74–0.91) and 0.81 (95% CI: 0.72–0.91), respectively (Table 1 and Figures 1A,B). Of note, the burdens of IHD-related deaths and DALYs were only elevated in the middle SDI regions, with an EAPC of 0.11 (95% CI: 0.03–0.18) and 0.07 (95% CI: 0–0.15), respectively. The most pronounced decreases in ASMR and the age-standardized DALY rate occurred in high-middle SDI regions, with an EAPC of −1.46 (95% CI: −1.68 to −1.24) and −1.43 (95% CI: −1.64 to −1.22), respectively (Table 1 and Figures 1C,D). These findings underscore the complex relationship between socio-demographic factors and IHD outcomes. Specifically, they indicate that while high-middle SDI regions have made notable progress in reducing the IHD burden, middle SDI regions face increasing challenges.

In addition, our findings indicated that the burdens of IHD-related prevalence, incidence, mortality, and DALYs were the highest in Asia over the study period, with the largest EAPC of 0.8 (95% CI: 0.73–0.87), 0.91 (95% CI: 0.83–0.98), 0.21 (95% CI: 0.08–0.34), and 0.16 (95% CI: 0.04–0.29), respectively (Supplementary Table 1). In 2021, Eastern Mediterranean region represented the highest burdens in IHD-related prevalence, incidence, mortality, and DALYs, with an ASPR of 281.097 per 100,000 persons (95% UI: 230.062–342.994), ASIR of 58.343 per 100,000 persons (95% UI: 36.455–83.352), ASMR of 12.452 per 100,000 persons (95% UI: 10.865–14.202), and age-standardized DALY rate of 737.118 per 100,000 persons (95% UI: 643.458–840.912), respectively (Supplementary Table 1). In terms of GBD regions, the sharpest declines in IHD prevalence and incidence were observed in high-income North America, with an EAPC of −0.98 (95% CI: −1.14 to −0.81) and −1.92 (95% CI: −2.17 to −1.67), respectively. Moreover, in 2021, Oceania had the largest burdens in IHD-related mortality and DALYs, with an ASMR of 12.122 per 100,000 persons (95% UI: 9.035–15.559) and age-standardized DALY rate of 694.445 per 100,000 persons (95% UI: 516.238–890.916), respectively (Supplementary Table 1). These different trends indicated the complex and diverse dynamics of IHD burden across regions.

### 3.3 National level of IHD

**Figure 2:**
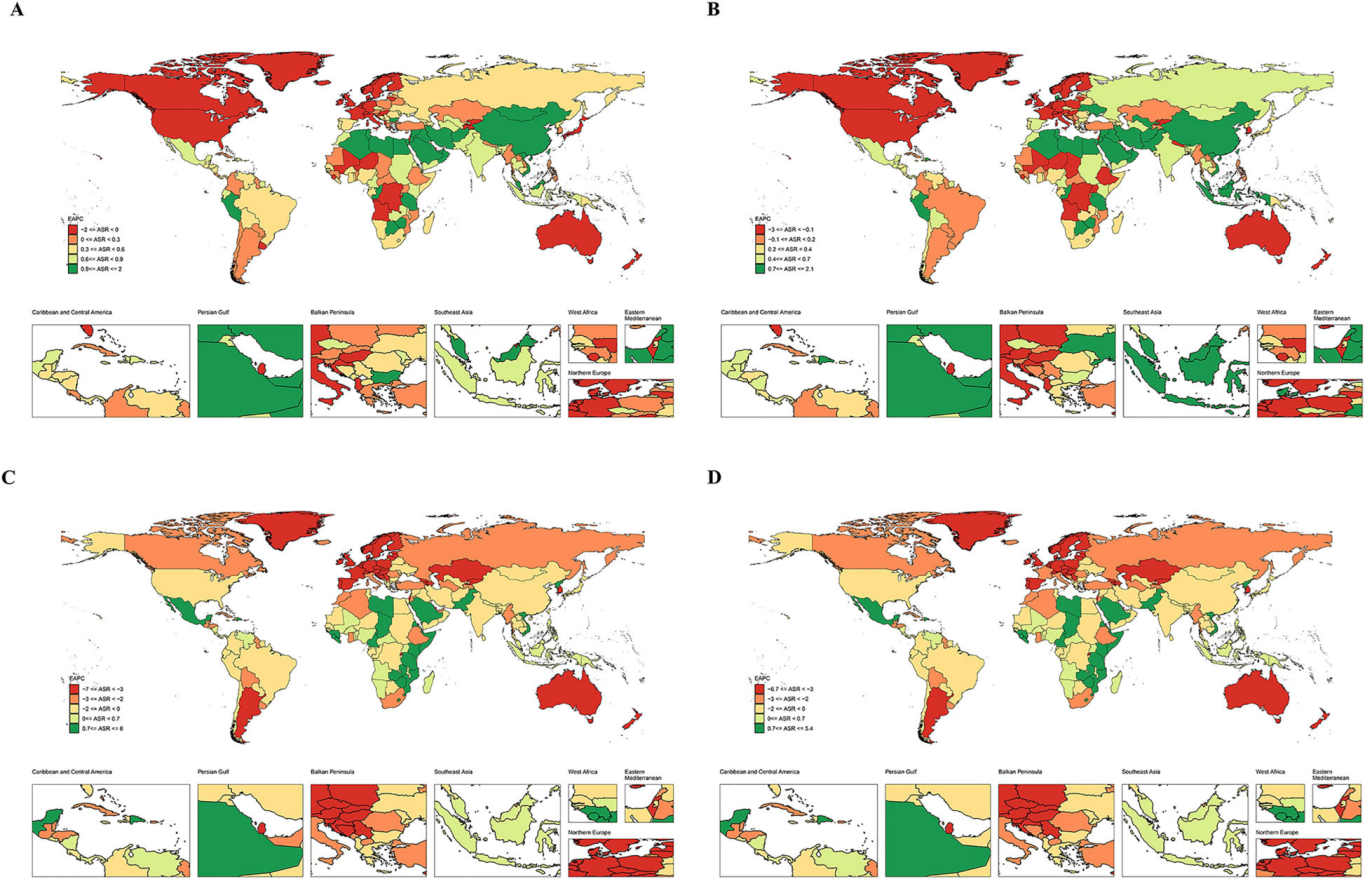
The EAPC map of IHD in 204 countries and territories. **(A)** Prevalence. **(B)** Incidence. **(C)** Deaths. **(D)** DALYs. EAPC, estimated annual percentage change; IHD, ischemic heart disease; DALYs, disability-adjusted life years.

The prevalence and incidence of IHD displayed national variation. The United Arab Emirates recorded the highest ASPR of 665.498 per 100,000 persons (95% UI: 554.772–793.996) and ASIR of 126.125 per 100,000 persons (95% UI: 71.883–194.315) in 2021 (Supplementary Table 2 and Figures 1A,B). From 1990 to 2021, the most pronounced decreases in ASPR and ASIR were observed in New Zealand, with an EAPC of −1.96 (95% CI: −2.25 to −1.66) and −2.83 (95% CI: −3.32 to −2.34), respectively (Supplementary Table 2 and Figures 2A,B). As for the deaths and DALYs of IHD, our data illustrated that Namibia had the highest ASMR of 42.583 per 100,000 persons (95% UI: 31.009–58.313) and age-standardized DALY rate of 2,430.916 per 100,000 persons (95% UI: 1,775.891–3,321.01) in 2021 (Supplementary Table 2 and Figures 1C,D). Notably, Lesotho showed the greatest increases in ASMR and age-standardized DALY rate, with the EAPC of 5.44 (95% CI: 4.75–6.13) and 5.32 (95% CI: 4.64–6), respectively (Supplementary Table 2 and Figures 2C,D). The significant variation in prevalence and incidence across countries underscores substantial disparities in IHD healthcare systems and prevention strategies. The ASMR and age-standardized DALY rate further highlighted these disparities.

### 3.4 Age and sex patterns

**Figure 3:**
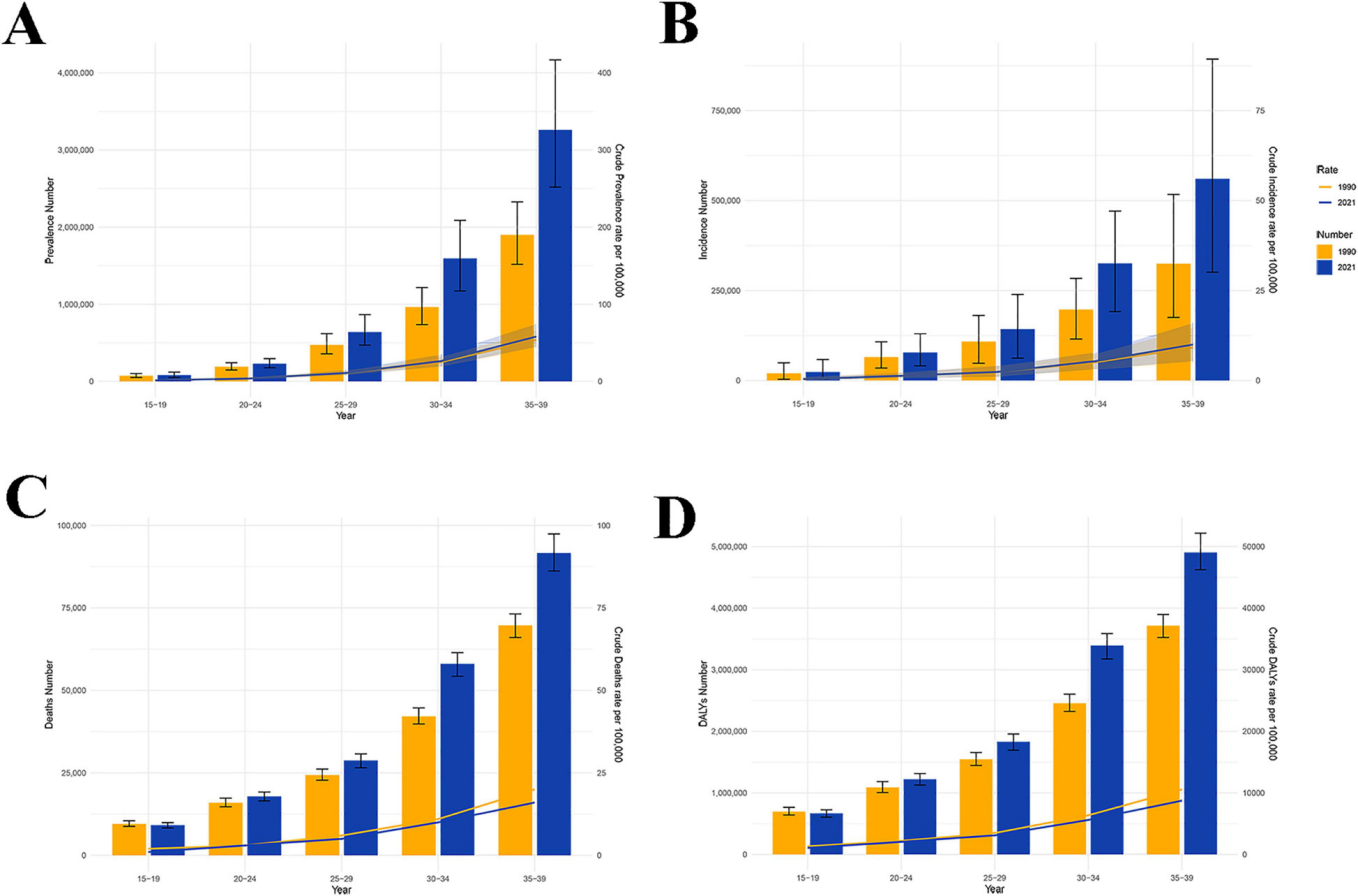
The age-specific numbers and rates of IHD. **(A)** Prevalence number and rate. **(B)** Incidence number and rate. **(C)** Death number and rate. **(D)** DALYs number and rate. IHD, ischemic heart disease; DALYs, disability-adjusted life years.

**Figure 4:**
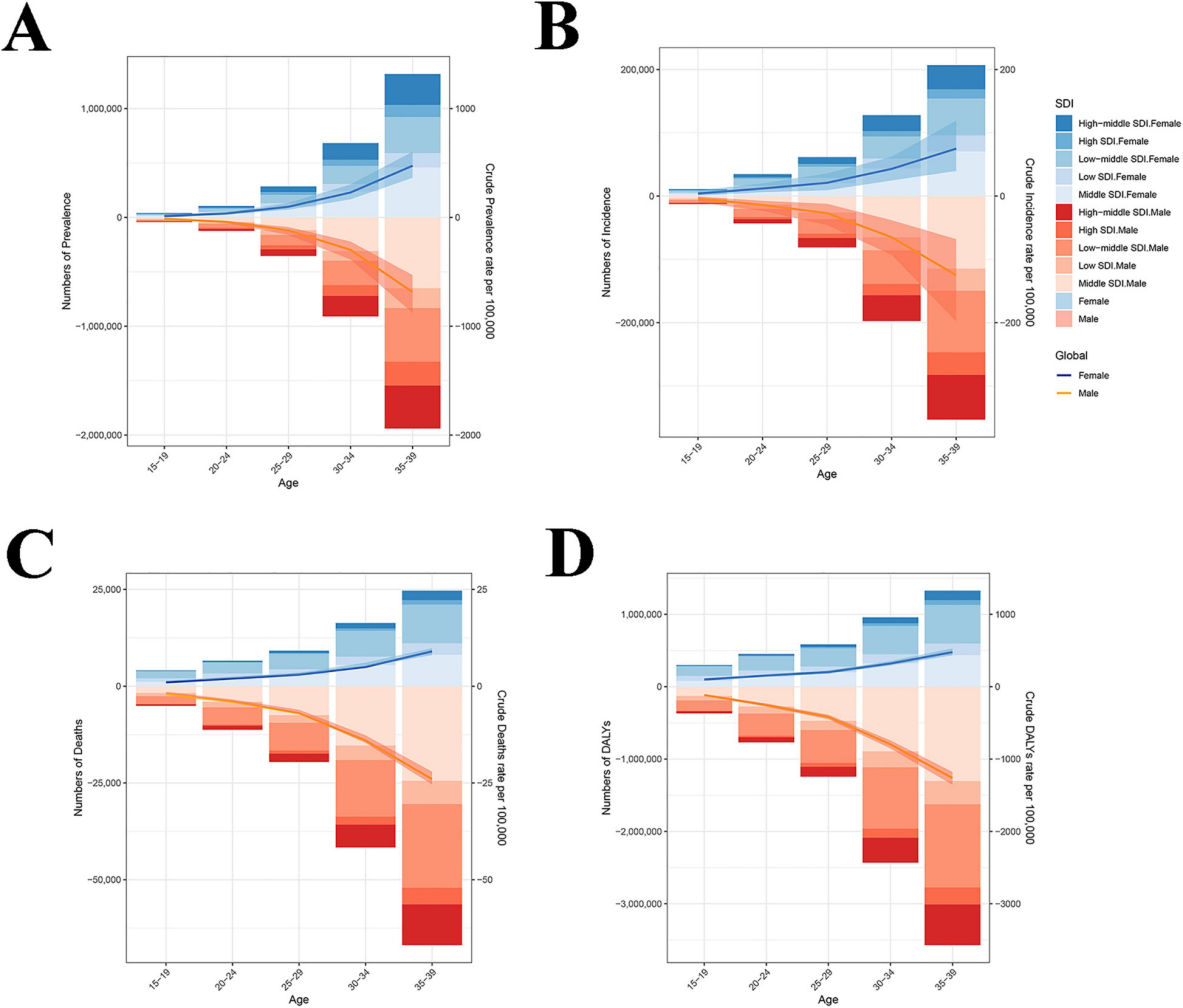
The sex-specific numbers and rates of IHD. **(A)** Prevalence. **(B)** Incidence. **(C)** Deaths. **(D)** DALYs. IHD, ischemic heart disease; SDI, socio-demographic index; DALYs, disability-adjusted life years.

In youths and young adults, the number of IHD-related prevalence, incidence, deaths and DALYs was elevated with age (Figure 3). In 1990 and 2021, the peaks of prevalence (Figure 3A), incidence (Figure 3B), deaths (Figure 3C) and DALYs (Figure 3D) were found in the 35–39 years age group. Globally, the number of IHD-related prevalence (1,941,642 cases), incidence (353,508 cases), deaths (66,953 cases), and DALYs (3,575,042 cases) peaked in the 35–39 years age group across males. Similar phenomena were observed across females (Figure 4). In the middle SDI regions, both males and females had the highest prevalence (650,541 and 459,665 cases) and incidence (114,384 and 70,565 cases) in the 35–39 years age group (Figures 4A,B). In males, the number of DN-related deaths (24,411 cases) and DALYs (1,302,985 cases) in the 35–39 years age group were the highest in the middle SDI regions, while in females, they peaked at 10,020 and 535,240 cases in the low-middle SDI regions (Figures 4C,D). These findings indicated that there was a greater overall burden in males than females, especially in the 35–39 years age group.

### 3.5 Risk factors for IHD

**Figure 5:**
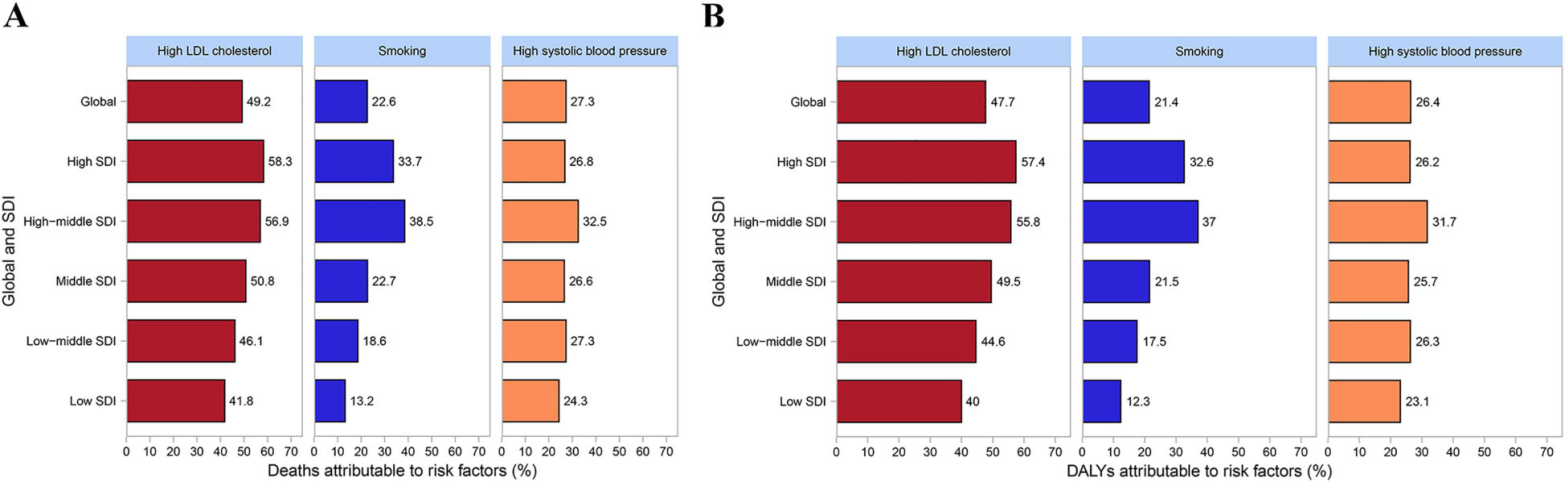
Risk factors for deaths and DALYs globally and in different SDI regions. **(A)** Risk factor for deaths. **(B)** Risk factor for DALYs. DALYs, disability-adjusted life years; SDI, socio-demographic index.

Subsequently, the risk factors for IHD were explored globally and in different SDI regions. Our data indicated that the major risk factors for IHD-related deaths and DALYs were high low-density lipoprotein cholesterol (LDL-C), smoking and high SBP, orderly (Figure 5). Globally, high LDL-C was the leading attributable risk factor to IHD deaths, with a share of up to 49.2%. The contributions of smoking and high SBP to IHD deaths were 22.6% and 27.3% globally. Among five SDI regions, high LDL-C had a maximum proportion (58.3%) in the high SDI regions. The proportions of smoking (38.5%) and high SBP (32.5%) were the highest in the high-middle SDI regions (Figure 5A). On the other hand, high LDL-C was the largest risk factor for IHD-related DALYs, accounting for 47.7% globally. Smoking (21.4%) and high SBP (26.4%) also made important contributions to DALYs globally. Moreover, the highest proportion of DALYs (57.4%) due to high LDL-C was found in the high SDI regions, while the lowest proportion (40%) was observed in the low SDI regions. And the highest proportions of smoking (37%) and high SBP (31.7%) were observed in the high-middle SDI regions (Figure 5B). These risk factors act complex ways to impact the global burden of IHD.

### 3.6 Future tendency of IHD

**Figure 6:**
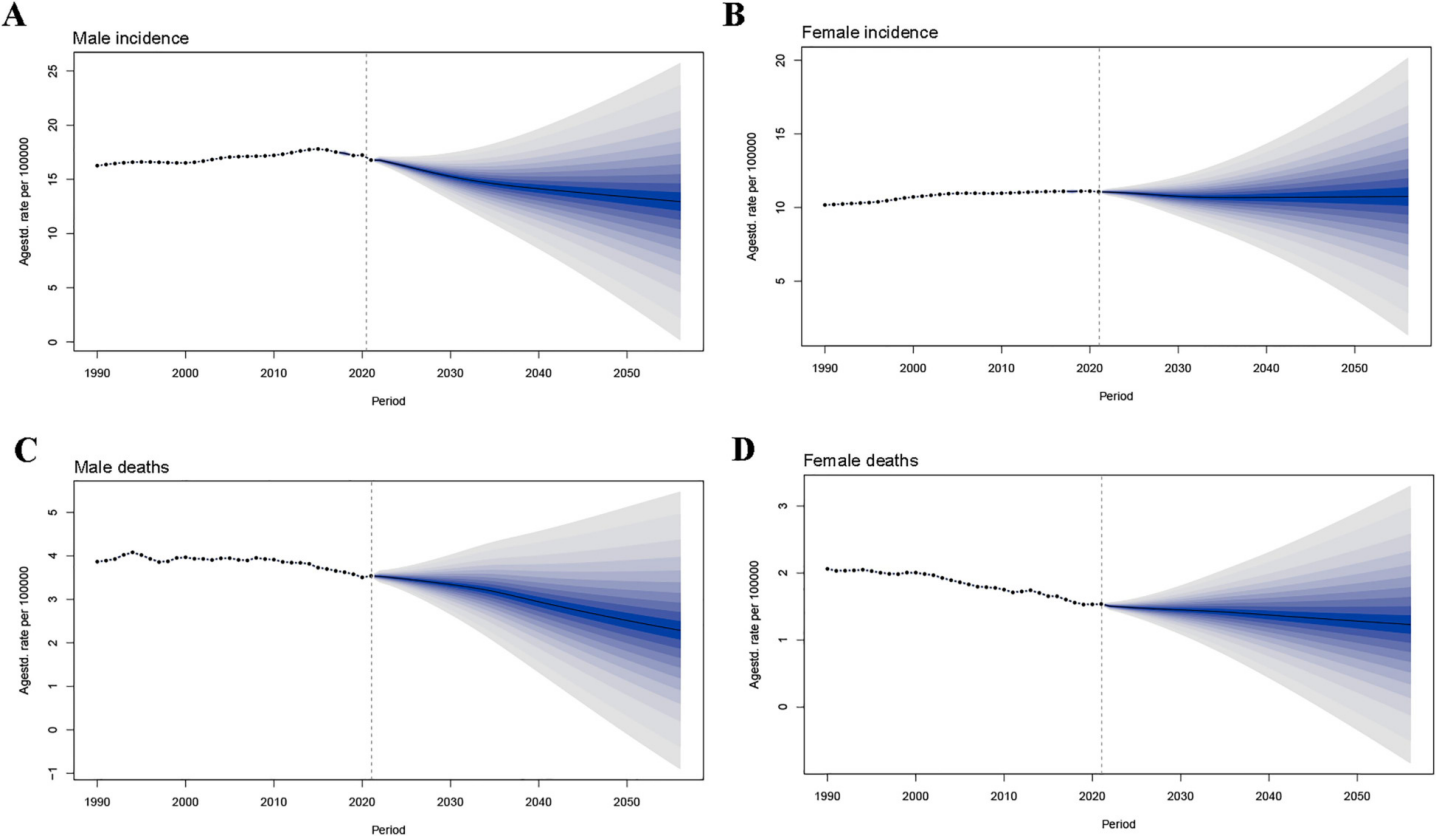
Future forecasts of global burden of IHD by 2050. **(A)** Male incidence. **(B)** Female incidence. **(C)** Male deaths. **(D)** Female deaths. IHD, ischemic heart disease.

Projections for the period 2021–2050 suggest a decline in the global burden of IHD, alongside falling incidence and death rates in both males and females (Figure 6). The global ASIR of IHD in males is predicted to decrease from 16.78 per 100,000 persons in 2021 to 13.38 per 100,000 persons in 2050 (Figure 6A). A similar trend was observed in females. Our model projects that the global ASIR of IHD will continue to decline from 11.07 per 100,000 persons in 2021 to 10.73 per 100,000 persons in 2050 (Figure 6B). As for the deaths, the global ASMR of IHD was reduced in males (from 3.54 per 100,000 persons to 2.52 per 100,000 persons) and females (from 1.54 per 100,000 persons to 1.28 per 100,000 persons), and the decrease was greater in males than females (Figures 6C,D). In general, the decreases of global burden in IHD-related incidence and deaths were larger in males than females.

## 4 Discussion

In recent years, IHD has become one of the major global public health challenges (1, 15). The GBD 2021 study includes data on the burden of IHD from countries around the world, which helps estimate the overall burden of IHD among youths and young adults. According to GBD 2021 data, our outcomes reveal that, in 2021, the number of youths and young adults suffering from IHD globally reached 5,805,593 cases, with 205,513 deaths. This represents a substantial portion of the global IHD burden, highlighting its considerable impact on public health. In present study, we further utilized GBD 2021 data to reveal the temporal trends and attributable factors of the global IHD burden in youths and young adults, and explore the projected changes over the next 30 years. These findings will contribute to the development of targeted prevention and control strategies for IHD, helping to reduce the disease burden.

In recent years, many GBD reports have focused on the global burden trends of IHD, including prevalence, incidence, deaths and DALYs, which help identify high-risk populations and regions (16–18). The GBD 2017 report shows that, in China, the age-standardized DALYs for stroke decreased by 33.1% per 100,000 population, with IHD increasing by 4.6% from 1990 to 2017 (17). Globally, in 2019, the total DALYs due to IHD reach 182 million, with 9.14 million deaths (16). Worldwide, the mortality for IHD has been decreasing, especially in some high-income countries (North America and Europe), where medical advancements, early screening, and the promotion of healthy lifestyles have contributed to a yearly reduction in IHD mortality (9). In contrast, low-income developing countries show different epidemiological trends for IHD compared to high-income developed nations (19, 20). In India, the incidence and mortality for IHD have been rising, and the burden of IHD is increasingly affecting relatively younger populations (19). This may be related to changes in lifestyle among younger groups in recent years, particularly the spread of Western diets, lack of exercise, and increased unhealthy habits of smoking and alcohol consumption. Data from GBD 2019 further shows that the prevalence and incidence of IHD among youths and young adults worldwide have increased in 2019 (7). According to the latest GBD 2021 data, our study indicates that the burdens of IHD-related deaths and DALYs in youths and young adults have decreased, yet the prevalence and incidence have increased. It is expected that by 2050, the incidence and mortality of IHD in youths and young adults will decrease, likely due to ongoing improvements in global public health and medical standards.

The global burden of IHD in youths and young adults varies significantly across regions with different SDI levels (7, 21). GBD 2019 study demonstrates that low and low-middle SDI regions face a higher burden of IHD compared to those regions with high and high-middle SDI (7). Our findings indicate that, from 1990 to 2021, the middle-SDI quintile experienced the largest increase in the overall IHD burden among youths and young adults, whereas the high-middle-SDI quintile recorded the greatest reductions in both deaths and DALYs. Therefore, each region should develop targeted IHD prevention and control strategies based on its socio-economic development level to effectively reduce the disease burden, especially in the middle SDI regions. Furthermore, the GBD 2019 study illustrates that the IHD burden in Oceania significantly exceed expectations based on its development level (22). Similarly, the GBD 2021 data in this study indicate that Oceania has the highest burdens in IHD-related deaths and DALYs. These results highlight the disparities between IHD burden and healthcare quality, underscoring the need for targeted health interventions to prevent and treat IHD in youths and young adults in the future.

Our findings indicate that, among youths and young adults, the incidence of ischaemic heart disease rises with age and is consistently higher in males than in females. Additionally, our data reveal that the burden of IHD is greatest in the 35–39 age group. In recent years, as urbanization has accelerated, unhealthy dietary patterns (high sugar, high fat, and high salt) have become more prevalent. Simultaneously, the increasing academic and work pressures faced by youths and young adults may have a profound impact on cardiovascular health (23–25). Furthermore, the relationship between psychological stress and heart health is gaining increasing attention (26, 27). Occupational stress, caregiving burden, and perceived social expectations have been associated with chronic psychological stress, which is linked to adverse cardiovascular outcomes, including hypertension and related cardiometabolic disorders. The 35–39 age group is a period of heightened life stress for many individuals, especially for men, where the accumulation of psychological pressure may further increase the risk of heart disease. The risk of IHD is generally higher in men than in women, possibly due to hormonal levels and biological differences (28). Before menopause, women benefit from the protective effect of estrogen, which helps maintain vascular health, lower blood cholesterol levels, and reduce the risk of IHD (29). Moreover, men may face a higher risk of cardiovascular diseases, including IHD, due to higher rates of smoking, drinking, and elevated lipid levels in their youth (30). Therefore, early identification and intervention, particularly health education and lifestyle changes within younger populations, are crucial for reducing the burden of IHD.

In this study, our data further indicates that the major risk factors for IHD-related deaths and DALYs in youths and young adults are high LDL-C, smoking, and high SBP. These findings are consistent with GBD 2019 study (7). A recent investigation focused on youths aged 15–49 years also demonstrated that high level of LDL-C, high SBP and smoking were leading risk factors for youths IHD (2). High LDL-C is considered one of the primary risk factors for IHD. In recent years, many researches have supported this view regarding the relationship between LDL-C and IHD (31–33). The mechanism by which high LDL-C triggers IHD mainly involves promoting endothelial dysfunction, inducing inflammatory responses, and increasing oxidative stress (34, 35). A recent study has shown that lowering LDL-C levels can effectively reduce the incidence and mortality of IHD (36). Smoking is another important modifiable risk factor for IHD. Smoking not only directly damages the cardiovascular system but also increases the risk of IHD through mechanisms such as elevated levels of carbon monoxide, free fatty acids, and platelet aggregation (37). Numerous epidemiological studies have shown a close association between smoking and the incidence of IHD (37, 38). The evidence indicates that smokers have a significantly higher risk of developing IHD compared to non-smokers (39). Additionally, high SBP is one of the major risk factors for IHD. High SBP induces mechanical stress on the vessel walls, leading to endothelial dysfunction and promoting the development and progression of IHD (40). Several studies have shown that antihypertensive treatment can effectively reduce the risk of IHD (40, 41). One clinical trial indicates that systematic antihypertensive treatment can reduce the risk of cardiovascular events in hypertensive patients by about 10% (42). Recently, as the global burden of IHD increases, especially in low- and middle-income countries, controlling these primary risk factors becomes particularly important. Future public health strategies should focus on these high-risk factors and implement targeted interventions to effectively reduce the global burden of IHD.

Our analysis of GBD 2021 data delineates the trajectory of IHD burden among youths and young adults from 1990 to 2021, a period largely preceding the COVID-19 pandemic. However, the global pandemic presents a potential paradigm shift for cardiovascular health. Emerging research indicates that SARS-CoV-2 infection is associated with both acute and long-term increases in cardiovascular risk (43). A recent study has indicated that myocardial injury had the strongest association for adverse COVID-19 outcomes (44). While the full impact of these sequelae on population-level IHD burden will require future GBD analyses to quantify, the widespread nature of the pandemic suggests a potential threat to the favorable trends. This underscores the critical need for enhanced cardiovascular monitoring and preventive strategies for young populations in the post-COVID-19 era and highlights the importance of future updates to the GBD study to track the evolving burden of IHD.

This study provides a comprehensive and high-quality assessment of the global burden of IHD among individuals aged 15–39 years. The main strength of this study lies in its analysis, which covers factors of age, gender, country, region, and socioeconomic status. Additionally, the study presents time trends for the EAPC from 1990 to 2021, rather than just total percentage changes, which enhances the accuracy of the findings. However, some limitations of the study should also be acknowledged. Firstly, although the GBD relies on predictive covariate values to estimate the IHD burden in different regions, the prevalence, incidence, mortality, and DALYs in low and middle SDI countries may be underestimated. For some regions, particularly low- and middle-income countries, data on IHD incidence and mortality may be sparse, incomplete, or derived from verbal autopsy, which can lead to underreporting or misclassification of IHD cases against other cardiovascular causes of death. Secondly, due to the asymptomatic and mild nature of IHD in youths and young adults, the IHD data in the GBD database may introduce bias in the causal distribution of IHD. Thirdly, the interactions between IHD comorbidities and risk factors for young people remain unknown, and future well-designed studies are needed. Fourthly, the use of EAPC to summarize trends has its own limitations. The EAPC provides a single average estimate of the annual change rate over the entire study period, assuming a constant exponential trend. Consequently, it may mask more complex, non-linear temporal patterns, such as periods of rapid decline followed by stabilization or even an increase in rates. For instance, significant changes in diagnostic criteria, treatment guidelines, or public health interventions during the study period could create inflection points that the EAPC model cannot capture. Finally, the predictive model used in this study is limited to forecasting the burden trend of IHD by gender, and the future development trends of IHD across different regions and age groups remain unclear. Despite these limitations, our research results provide valuable insights and guidance for the development of global public health policies related to IHD.

## 5 Conclusions

In conclusion, from 1990 to 2021, the burdens of IHD-related adverse outcomes in youths and young adults are lessened overall, while the prevalence and incidence have increased. The burdens of IHD-related incidence and deaths are expected to decline over the next 30 years. The greatest increase in IHD burden has been observed in the middle SDI regions. Moreover, IHD predominantly affects males compared to females, especially in the 35–39 years age group. High LDL-C, smoking, and high SBP remain the major risk factors for IHD-related deaths and DALYs. These findings will provide critical support for mitigating the burden of IHD in youths and young adults. Future research should focus on personalized treatment and more effective disease prevention strategies, particularly in the middle SDI regions, to alleviate the global public health burden of IHD.

## Data Availability

The original contributions presented in the study are included in the article/Supplementary Material, further inquiries can be directed to the corresponding author.

## Supplementary material

Supplementary Figure 1 The ASR map of IHD in 204 countries and territories. **(A)** Prevalence. **(B)** Incidence. **(C)** Deaths. **(D)** DALYs. ASR, age-standardized rate; IHD, ischemic heart disease; DALYs, disability-adjusted life years.
