# Supplementary material for: Global, regional, and national burden of ischemic heart disease in youths and young adults aged 15–39 years in 204 countries/territories, 1990–2021: a systematic analysis of global burden of disease study 2021
Source: Front Cardiovasc Med. 2025 Oct 14;12:1649408. doi: 10.3389/fcvm.2025.1649408 (PMC12559993; doi:10.3389/fcvm.2025.1649408)
Supplement: Supplementary file 1 [file Table1.docx]

**Supplementary Table 1 regional trends in IHD burden: prevalence, incidence, deaths and disability-adjusted life years (1990-2021).**

| **Location name** | **1990** | | | **2021** | | \| **EAPC (95% CI)** \| \| --- \| \| |
| --- | --- | --- | --- | --- | --- | --- | --- |
|  | **Number** | **ASR (95% UI)** | | **Number** | **ASR (95% UI)** |  |
| **Prevalence** |  |  | |  |  |  |
| Continents |  |  |  | |  |  |
| Africa | 312868 (262481-362876) | 130.101 (109.148-150.896) | 812322 (651852-979919) | | 146.464 (117.531-176.682) | 0.41 (0.37 to 0.44) |
| America | 436714 (354854-528546) | 148.902 (120.991-180.213) | 616368 (484758-765219) | | 161.345 (126.894-200.309) | 0.03 (-0.04 to 0.11) |
| Asia | 2146494 (1708855-2659813) | 159.019 (126.597-197.047) | 3717215 (2892499-4742277) | | 210.083 (163.473-268.016) | 0.8 (0.73 to 0.87) |
| Europe | 695988 (576242-823675) | 228.457 (189.15-270.369) | 651631 (525364-801543) | | 245.579 (197.993-302.077) | 0.13 (-0.01 to 0.27) |
| WHO regions |  |  |  | |  |  |
| African Region | 210946 (173495-249048) | 109.67 (90.199-129.479) | 566769 (445615-695394) | | 122.396 (96.233-150.173) | 0.36 (0.34 to 0.38) |
| Eastern Mediterranean Region | 297290 (252451-347383) | 207.008 (175.786-241.889) | 879496 (719818-1073159) | | 281.097 (230.062-342.994) | 1.08 (1 to 1.17) |
| European Region | 738473 (612661-872207) | 225.586 (187.153-266.438) | 725279 (586347-888599) | | 243.116 (196.546-297.862) | 0.12 (-0.02 to 0.27) |
| Region of the Americas | 436714 (354854-528546) | 148.902 (120.991-180.213) | 616368 (484758-765219) | | 161.345 (126.894-200.309) | 0.03 (-0.04 to 0.11) |
| South-East Asia Region | 774055 (616509-958809) | 146.576 (116.743-181.562) | 1596815 (1231619-2033253) | | 183.852 (141.804-234.102) | 0.72 (0.68 to 0.76) |
| Western Pacific Region | 1116301 (869360-1408074) | 160.78 (125.213-202.803) | 1394813 (1061381-1802497) | | 218.28 (166.1-282.08) | 0.76 (0.58 to 0.94) |
| Global burden of disease regions |  |  |  | |  |  |
| East Asia | 1013444 (783459-1279407) | 179.147 (138.492-226.161) | 1227307 (917082-1601566) | | 256.197 (191.438-334.323) | 0.91 (0.73 to 1.1) |
| Oceania | 3217 (2712-3764) | 121.12 (102.079-141.685) | 7615 (6186-9132) | | 135.145 (109.789-162.077) | 0.31 (0.27 to 0.35) |
| Southeast Asia | 204076 (164823-246769) | 103.59 (83.665-125.26) | 363740 (284244-455009) | | 131.16 (102.494-164.07) | 0.74 (0.72 to 0.76) |
| Central Sub-Saharan Africa | 19796 (16453-23097) | 95.344 (79.244-111.246) | 52555 (41484-63186) | | 97.152 (76.686-116.803) | -0.04 (-0.07 to -0.01) |
| Eastern Sub-Saharan Africa | 67095 (54940-79609) | 94.647 (77.501-112.3) | 184575 (143441-227265) | | 105.36 (81.88-129.728) | 0.41 (0.35 to 0.46) |
| Southern Sub-Saharan Africa | 28048 (22311-34331) | 129.76 (103.219-158.828) | 54898 (42516-69411) | | 161.294 (124.913-203.934) | 0.54 (0.39 to 0.68) |
| Western Sub-Saharan Africa | 77420 (63040-92905) | 108.167 (88.077-129.802) | 230578 (180949-284278) | | 120.59 (94.634-148.674) | 0.43 (0.39 to 0.46) |
| South Asia | 698259 (551542-872909) | 161.778 (127.785-202.242) | 1573492 (1212912-2025321) | | 198.942 (153.353-256.069) | 0.65 (0.59 to 0.71) |
| Andean Latin America | 19361 (16460-22279) | 125.203 (106.444-144.073) | 43795 (36433-52558) | | 161.728 (134.54-194.086) | 0.91 (0.83 to 0.99) |
| Caribbean | 29635 (25500-33902) | 199.368 (171.546-228.07) | 44105 (36226-52156) | | 242.297 (199.014-286.527) | 0.41 (0.28 to 0.53) |
| Central Latin America | 106872 (87895-126785) | 156.549 (128.752-185.719) | 189033 (152292-233903) | | 186.861 (150.542-231.215) | 0.51 (0.48 to 0.53) |
| Tropical Latin America | 97868 (76716-121114) | 152.175 (119.285-188.32) | 156554 (119465-197173) | | 177.279 (135.28-223.275) | 0.39 (0.33 to 0.45) |
| North Africa and Middle East | 300869 (261326-345150) | 224.815 (195.268-257.903) | 794615 (656508-956308) | | 312.513 (258.197-376.105) | 1.11 (1.02 to 1.21) |
| Central Asia | 56285 (48591-64650) | 197.814 (170.771-227.212) | 91054 (74824-108110) | | 243.544 (200.135-289.166) | 0.32 (0.12 to 0.53) |
| Central Europe | 143514 (121577-166892) | 306.336 (259.511-356.239) | 110619 (89211-132899) | | 315.875 (254.744-379.496) | 0.23 (0.04 to 0.42) |
| Eastern Europe | 318580 (248910-401369) | 371.442 (290.212-467.967) | 306237 (231227-400606) | | 462.78 (349.426-605.389) | 0.56 (0.25 to 0.86) |
| Australasia | 9053 (7580-10688) | 111.032 (92.964-131.077) | 10319 (8217-12615) | | 98.552 (78.472-120.477) | -0.86 (-1.02 to -0.7) |
| High-income Asia Pacific | 53059 (42563-65336) | 78.612 (63.061-96.8) | 40578 (31476-50707) | | 80.289 (62.281-100.332) | 0 (-0.09 to 0.09) |
| High-income North America | 170864 (133038-214562) | 150.786 (117.405-189.349) | 163482 (123020-211712) | | 132.713 (99.867-171.866) | -0.98 (-1.14 to -0.81) |
| Southern Latin America | 16628 (13829-19563) | 87.155 (72.482-102.535) | 23852 (19298-28930) | | 92.464 (74.812-112.149) | 0.1 (0.06 to 0.14) |
| Western Europe | 165899 (137706-197893) | 115.112 (95.55-137.312) | 136590 (108743-166680) | | 105.253 (83.795-128.44) | -0.68 (-0.79 to -0.56) |
| **Incidence** |  |  |  | |  |  |
| Continents |  |  |  | |  |  |
| Africa | 71244 (44007-100611) | 29.625 (18.3-41.837) | 175629 (107713-250805) | | 31.666 (19.421-45.221) | 0.26 (0.23 to 0.29) |
| America | 88748 (51730-130266) | 30.259 (17.638-44.415) | 110514 (66704-156847) | | 28.929 (17.461-41.057) | -0.39 (-0.47 to -0.3) |
| Asia | 424679 (261079-597893) | 31.462 (19.342-44.294) | 727896 (448690-1038825) | | 41.138 (25.358-58.711) | 0.91 (0.83 to 0.98) |
| Europe | 129329 (82360-179633) | 42.452 (27.034-58.964) | 115230 (71863-163887) | | 43.426 (27.083-61.764) | 0.05 (-0.04 to 0.14) |
| WHO regions |  |  |  | |  |  |
| African Region | 49372 (30009-70261) | 25.668 (15.602-36.528) | 125436 (76155-179293) | | 27.088 (16.446-38.719) | 0.19 (0.18 to 0.21) |
| Eastern Mediterranean Region | 64653 (40022-91407) | 45.019 (27.868-63.648) | 182544 (114060-260792) | | 58.343 (36.455-83.352) | 1.02 (0.93 to 1.12) |
| European Region | 137657 (87521-191461) | 42.051 (26.736-58.487) | 129253 (80298-183741) | | 43.326 (26.916-61.591) | 0.05 (-0.04 to 0.15) |
| Region of the Americas | 88748 (51730-130266) | 30.259 (17.638-44.415) | 110514 (66704-156847) | | 28.929 (17.461-41.057) | -0.39 (-0.47 to -0.3) |
| South-East Asia Region | 169901 (103544-241920) | 32.173 (19.607-45.81) | 333962 (205390-475700) | | 38.451 (23.648-54.77) | 0.7 (0.63 to 0.77) |
| Western Pacific Region | 200296 (123304-280543) | 28.848 (17.759-40.406) | 244403 (151635-349469) | | 38.248 (23.73-54.69) | 0.8 (0.58 to 1.02) |
| Global burden of disease regions |  |  |  | |  |  |
| East Asia | 185951 (115390-263094) | 32.871 (20.397-46.507) | 221526 (135322-317758) | | 46.243 (28.248-66.331) | 0.98 (0.76 to 1.2) |
| Oceania | 646 (387-914) | 24.321 (14.587-34.42) | 1523 (914-2176) | | 27.031 (16.213-38.621) | 0.3 (0.26 to 0.33) |
| Southeast Asia | 34465 (19981-49062) | 17.495 (10.142-24.904) | 55913 (32623-80507) | | 20.161 (11.763-29.03) | 0.56 (0.51 to 0.61) |
| Central Sub-Saharan Africa | 5090 (3081-7296) | 24.518 (14.838-35.141) | 13277 (7788-19235) | | 24.544 (14.396-35.556) | -0.08 (-0.13 to -0.03) |
| Eastern Sub-Saharan Africa | 15816 (9494-22494) | 22.311 (13.393-31.731) | 41043 (24592-58495) | | 23.428 (14.037-33.39) | 0.23 (0.2 to 0.26) |
| Southern Sub-Saharan Africa | 7130 (4327-10150) | 32.984 (20.019-46.957) | 13027 (7847-18687) | | 38.274 (23.054-54.905) | 0.35 (0.26 to 0.44) |
| Western Sub-Saharan Africa | 17514 (10643-25016) | 24.47 (14.87-34.952) | 49391 (29847-70207) | | 25.831 (15.61-36.718) | 0.26 (0.23 to 0.29) |
| South Asia | 160014 (98578-229100) | 37.073 (22.839-53.08) | 342500 (212275-486747) | | 43.304 (26.839-61.541) | 0.63 (0.56 to 0.71) |
| Andean Latin America | 3859 (2314-5533) | 24.958 (14.965-35.779) | 8469 (5094-12121) | | 31.275 (18.811-44.76) | 0.81 (0.74 to 0.87) |
| Caribbean | 6205 (3910-8757) | 41.745 (26.302-58.914) | 8966 (5516-12611) | | 49.256 (30.306-69.283) | 0.42 (0.34 to 0.51) |
| Central Latin America | 22428 (13813-31809) | 32.853 (20.234-46.595) | 38245 (23207-54567) | | 37.806 (22.941-53.94) | 0.45 (0.43 to 0.47) |
| Tropical Latin America | 17036 (10175-24444) | 26.489 (15.821-38.008) | 23187 (13796-33197) | | 26.257 (15.623-37.592) | -0.06 (-0.15 to 0.02) |
| North Africa and Middle East | 64552 (40068-91533) | 48.235 (29.94-68.396) | 161675 (100167-233553) | | 63.585 (39.395-91.854) | 0.97 (0.88 to 1.06) |
| Central Asia | 11142 (6813-15931) | 39.158 (23.945-55.988) | 17470 (10615-24951) | | 46.727 (28.391-66.737) | 0.27 (0.11 to 0.43) |
| Central Europe | 23981 (14521-34067) | 51.189 (30.995-72.717) | 15349 (9528-21874) | | 43.83 (27.206-62.462) | -0.46 (-0.64 to -0.29) |
| Eastern Europe | 63774 (40018-89248) | 74.356 (46.658-104.056) | 59357 (37492-82902) | | 89.7 (56.657-125.28) | 0.61 (0.43 to 0.79) |
| Australasia | 1810 (1095-2668) | 22.194 (13.424-32.721) | 1862 (1079-2900) | | 17.782 (10.305-27.698) | -1.1 (-1.28 to -0.92) |
| High-income Asia Pacific | 6647 (3581-10062) | 9.848 (5.305-14.908) | 5044 (2641-7772) | | 9.98 (5.226-15.379) | 0.04 (-0.04 to 0.12) |
| High-income North America | 37108 (19645-57502) | 32.747 (17.336-50.745) | 28288 (17073-40363) | | 22.964 (13.86-32.766) | -1.92 (-2.17 to -1.67) |
| Southern Latin America | 3016 (1717-4457) | 15.808 (9.001-23.361) | 4218 (2346-6250) | | 16.353 (9.096-24.229) | 0.12 (0.07 to 0.18) |
| Western Europe | 27284 (17753-37901) | 18.931 (12.318-26.298) | 20395 (11807-30851) | | 15.716 (9.098-23.773) | -0.91 (-1.01 to -0.82) |
| **Deaths** |  |  |  | |  |  |
| Continents |  |  |  | |  |  |
| Africa | 16968 (15575-18608) | 7.056 (6.477-7.738) | 28679 (24724-32678) | | 5.171 (4.458-5.892) | -0.92 (-0.96 to -0.88) |
| America | 14674 (14407-14926) | 5.003 (4.912-5.089) | 15462 (14655-16335) | | 4.047 (3.836-4.276) | -0.7 (-0.94 to -0.46) |
| Asia | 106266 (98399-115210) | 7.872 (7.29-8.535) | 150600 (140222-161793) | | 8.511 (7.925-9.144) | 0.21 (0.08 to 0.34) |
| Europe | 23492 (22946-23990) | 7.711 (7.532-7.875) | 10576 (9951-11300) | | 3.986 (3.75-4.259) | -2.81 (-3.12 to -2.5) |
| WHO regions |  |  |  | |  |  |
| African Region | 7047 (6267-7825) | 3.664 (3.258-4.068) | 14766 (12929-16721) | | 3.189 (2.792-3.611) | -0.55 (-0.66 to -0.43) |
| Eastern Mediterranean Region | 19678 (17836-21621) | 13.702 (12.42-15.055) | 38959 (33993-44437) | | 12.452 (10.865-14.202) | -0.2 (-0.27 to -0.13) |
| European Region | 26003 (25378-26623) | 7.943 (7.752-8.133) | 13125 (12338-13906) | | 4.399 (4.136-4.661) | -2.64 (-2.95 to -2.33) |
| Region of the Americas | 14674 (14407-14926) | 5.003 (4.912-5.089) | 15462 (14655-16335) | | 4.047 (3.836-4.276) | -0.7 (-0.94 to -0.46) |
| South-East Asia Region | 56562 (51162-62777) | 10.711 (9.688-11.888) | 84991 (77653-91612) | | 9.786 (8.941-10.548) | -0.26 (-0.48 to -0.05) |
| Western Pacific Region | 37189 (33417-40893) | 5.356 (4.813-5.89) | 37863 (33095-43260) | | 5.925 (5.179-6.77) | 0.15 (0.03 to 0.26) |
| Global burden of disease regions |  |  |  | |  |  |
| East Asia | 29934 (26262-33839) | 5.291 (4.642-5.982) | 27701 (23251-32970) | | 5.782 (4.854-6.882) | 0.04 (-0.12 to 0.2) |
| Oceania | 288 (209-381) | 10.838 (7.883-14.353) | 683 (509-877) | | 12.122 (9.035-15.559) | 0.33 (0.23 to 0.42) |
| Southeast Asia | 18108 (16281-20137) | 9.192 (8.264-10.221) | 26165 (22941-30259) | | 9.435 (8.272-10.911) | 0.09 (0.01 to 0.17) |
| Central Sub-Saharan Africa | 613 (451-816) | 2.953 (2.172-3.93) | 1504 (1112-1972) | | 2.781 (2.056-3.645) | -0.26 (-0.33 to -0.19) |
| Eastern Sub-Saharan Africa | 2384 (2067-2844) | 3.364 (2.916-4.012) | 6015 (4977-7233) | | 3.433 (2.841-4.129) | -0.1 (-0.19 to -0.02) |
| Southern Sub-Saharan Africa | 1014 (915-1134) | 4.691 (4.232-5.246) | 1172 (1036-1355) | | 3.443 (3.045-3.982) | -1.21 (-2.07 to -0.34) |
| Western Sub-Saharan Africa | 1815 (1428-2169) | 2.536 (1.995-3.031) | 5026 (3792-6116) | | 2.628 (1.983-3.199) | 0.21 (0.05 to 0.37) |
| South Asia | 47311 (41511-53074) | 10.961 (9.618-12.296) | 81951 (74020-88950) | | 10.361 (9.359-11.246) | -0.13 (-0.34 to 0.08) |
| Andean Latin America | 920 (816-1040) | 5.948 (5.278-6.723) | 1057 (879-1272) | | 3.904 (3.245-4.698) | -1.45 (-1.82 to -1.08) |
| Caribbean | 1016 (935-1108) | 6.838 (6.289-7.451) | 1143 (948-1357) | | 6.281 (5.21-7.458) | -0.06 (-0.37 to 0.24) |
| Central Latin America | 3400 (3314-3484) | 4.98 (4.854-5.104) | 5632 (5100-6159) | | 5.568 (5.041-6.088) | 0.5 (0.05 to 0.94) |
| Tropical Latin America | 3954 (3808-4096) | 6.148 (5.921-6.369) | 3891 (3726-4064) | | 4.406 (4.22-4.602) | -1.01 (-1.31 to -0.72) |
| North Africa and Middle East | 19345 (17736-21388) | 14.455 (13.253-15.982) | 26982 (23051-31703) | | 10.612 (9.066-12.468) | -0.9 (-1.01 to -0.79) |
| Central Asia | 3418 (3181-3655) | 12.014 (11.179-12.847) | 3103 (2728-3491) | | 8.299 (7.297-9.339) | -2.48 (-2.92 to -2.04) |
| Central Europe | 5053 (4933-5177) | 10.786 (10.53-11.051) | 1127 (1037-1222) | | 3.218 (2.961-3.49) | -4.02 (-4.37 to -3.66) |
| Eastern Europe | 10210 (9714-10634) | 11.904 (11.326-12.398) | 6228 (5672-6853) | | 9.412 (8.571-10.357) | -1.98 (-2.54 to -1.41) |
| Australasia | 266 (247-285) | 3.258 (3.028-3.495) | 137 (124-151) | | 1.305 (1.183-1.444) | -3.3 (-3.61 to -2.98) |
| High-income Asia Pacific | 1722 (1599-1859) | 2.551 (2.369-2.755) | 654 (619-696) | | 1.293 (1.224-1.377) | -2.36 (-2.69 to -2.04) |
| High-income North America | 4576 (4488-4666) | 4.038 (3.961-4.118) | 3278 (3083-3429) | | 2.661 (2.503-2.784) | -1.56 (-1.73 to -1.39) |
| Southern Latin America | 919 (858-980) | 4.815 (4.498-5.138) | 529 (494-563) | | 2.05 (1.914-2.184) | -2.53 (-2.95 to -2.1) |
| Western Europe | 5394 (5242-5557) | 3.742 (3.638-3.856) | 1535 (1483-1589) | | 1.183 (1.143-1.224) | -3.72 (-3.87 to -3.58) |
| **Disability-adjusted life years** |  |  |  | |  |  |
| Continents |  |  |  | |  |  |
| Africa | 1018076 (936058-1117382) | 423.35 (389.244-464.645) | 1715959 (1479641-1950373) | | 309.392 (266.783-351.657) | -0.92 (-0.96 to -0.88) |
| America | 855781 (839205-871433) | 291.786 (286.135-297.123) | 910027 (864897-960784) | | 238.215 (226.401-251.501) | -0.64 (-0.88 to -0.41) |
| Asia | 6280597 (5819087-6813272) | 465.286 (431.096-504.748) | 8777592 (8157781-9429687) | | 496.077 (461.048-532.931) | 0.16 (0.04 to 0.29) |
| Europe | 1333632 (1302920-1363812) | 437.761 (427.68-447.668) | 603331 (567765-643886) | | 227.377 (213.973-242.66) | -2.77 (-3.07 to -2.46) |
| WHO regions |  |  |  | |  |  |
| African Region | 420877 (375416-468100) | 218.812 (195.177-243.363) | 884371 (773904-1001175) | | 190.984 (167.128-216.208) | -0.54 (-0.65 to -0.42) |
| Eastern Mediterranean Region | 1180206 (1071323-1298067) | 821.799 (745.982-903.867) | 2306295 (2013253-2631048) | | 737.118 (643.458-840.912) | -0.24 (-0.31 to -0.17) |
| European Region | 1478900 (1442202-1512601) | 451.768 (440.558-462.063) | 750979 (706771-794621) | | 251.731 (236.912-266.36) | -2.59 (-2.9 to -2.29) |
| Region of the Americas | 855781 (839205-871433) | 291.786 (286.135-297.123) | 910027 (864897-960784) | | 238.215 (226.401-251.501) | -0.64 (-0.88 to -0.41) |
| South-East Asia Region | 3334372 (3011221-3696986) | 631.403 (570.21-700.068) | 4944158 (4520094-5339420) | | 569.254 (520.428-614.763) | -0.32 (-0.54 to -0.1) |
| Western Pacific Region | 2203221 (1977052-2415320) | 317.328 (284.753-347.876) | 2202146 (1933802-2508989) | | 344.622 (302.628-392.641) | 0.11 (0.01 to 0.22) |
| Global burden of disease regions |  |  |  | |  |  |
| East Asia | 1776115 (1558517-2000112) | 313.964 (275.499-353.56) | 1606662 (1352431-1906918) | | 335.386 (282.316-398.064) | 0 (-0.15 to 0.14) |
| Oceania | 16485 (11959-21847) | 620.583 (450.194-822.445) | 39128 (29087-50198) | | 694.445 (516.238-890.916) | 0.33 (0.24 to 0.42) |
| Southeast Asia | 1071961 (962058-1191457) | 544.13 (488.343-604.786) | 1530027 (1342019-1765087) | | 551.706 (483.913-636.466) | 0.05 (-0.02 to 0.13) |
| Central Sub-Saharan Africa | 36326 (26967-48008) | 174.962 (129.886-231.23) | 88918 (66223-115755) | | 164.37 (122.417-213.98) | -0.26 (-0.33 to -0.2) |
| Eastern Sub-Saharan Africa | 145462 (126240-173232) | 205.195 (178.08-244.367) | 366055 (303071-439811) | | 208.953 (173-251.055) | -0.12 (-0.21 to -0.03) |
| Southern Sub-Saharan Africa | 58406 (52826-65193) | 270.205 (244.393-301.606) | 67699 (60222-77994) | | 198.903 (176.937-229.152) | -1.18 (-2.03 to -0.32) |
| Western Sub-Saharan Africa | 107905 (85465-128851) | 150.759 (119.408-180.024) | 301351 (228014-365426) | | 157.603 (119.249-191.114) | 0.24 (0.08 to 0.4) |
| South Asia | 2785512 (2444556-3130370) | 645.367 (566.372-725.267) | 4776576 (4309909-5188738) | | 603.92 (544.917-656.031) | -0.18 (-0.39 to 0.03) |
| Andean Latin America | 56320 (49952-63706) | 364.211 (323.026-411.974) | 64582 (54219-76954) | | 238.491 (200.223-284.179) | -1.44 (-1.79 to -1.08) |
| Caribbean | 59800 (55059-65059) | 402.297 (370.404-437.674) | 66880 (55465-79256) | | 367.418 (304.706-435.409) | -0.07 (-0.38 to 0.24) |
| Central Latin America | 203351 (198305-208398) | 297.875 (290.483-305.268) | 334217 (303632-364299) | | 330.376 (300.143-360.113) | 0.49 (0.05 to 0.93) |
| Tropical Latin America | 228071 (219519-235901) | 354.627 (341.329-366.801) | 227467 (218609-237639) | | 257.579 (247.548-269.098) | -0.93 (-1.23 to -0.64) |
| North Africa and Middle East | 1160250 (1065101-1277540) | 866.961 (795.864-954.602) | 1598066 (1369168-1871696) | | 628.501 (538.478-736.117) | -0.94 (-1.04 to -0.84) |
| Central Asia | 197582 (183625-211127) | 694.399 (645.347-742.002) | 179324 (157832-201495) | | 479.644 (422.159-538.947) | -2.45 (-2.88 to -2.01) |
| Central Europe | 284415 (277754-291300) | 607.097 (592.879-621.792) | 65050 (59792-70299) | | 185.751 (170.738-200.74) | -3.92 (-4.27 to -3.58) |
| Eastern Europe | 572039 (544373-595274) | 666.957 (634.7-694.048) | 348055 (316609-382226) | | 525.975 (478.454-577.614) | -1.95 (-2.52 to -1.38) |
| Australasia | 15277 (14258-16326) | 187.365 (174.859-200.222) | 7905 (7173-8662) | | 75.491 (68.503-82.725) | -3.26 (-3.57 to -2.95) |
| High-income Asia Pacific | 101909 (94537-109775) | 150.988 (140.065-162.64) | 38808 (36806-41180) | | 76.787 (72.827-81.481) | -2.37 (-2.68 to -2.06) |
| High-income North America | 260797 (255465-266375) | 230.151 (225.445-235.073) | 189467 (178267-198282) | | 153.808 (144.716-160.964) | -1.49 (-1.65 to -1.33) |
| Southern Latin America | 53846 (50372-57360) | 282.225 (264.012-300.642) | 31446 (29363-33464) | | 121.903 (113.827-129.727) | -2.49 (-2.91 to -2.07) |
| Western Europe | 311256 (302503-320630) | 215.97 (209.897-222.474) | 90625 (87239-94222) | | 69.834 (67.224-72.606) | -3.65 (-3.78 to -3.51) |

IHD, ischemic heart disease; WHO, World Health Organization; ASR, age-standardized rate; EAPC, estimated annual percentage change; UI, uncertainty interval; CI, confidence interval.
