# Supplementary material for: Global, regional, and national burden of ischemic heart disease in youths and young adults aged 15–39 years in 204 countries/territories, 1990–2021: a systematic analysis of global burden of disease study 2021
Source: Front Cardiovasc Med. 2025 Oct 14;12:1649408. doi: 10.3389/fcvm.2025.1649408 (PMC12559993; doi:10.3389/fcvm.2025.1649408)
Supplement: Supplementary file 2 [file Table2.docx]

**Supplementary Table 2 The global disease burden of IHD in 204 countries and territories.**

| **Location name** | **1990** | | **2021** | | **EAPC (95% CI)** |
| --- | --- | --- | --- | --- | --- |
|  | **Number** | **ASR (95% UI)** | **Number** | **ASR (95% UI)** |  |
| **Prevalence** |  |  |  |  |  |
| Afghanistan | 5163 (4480-5889) | 163.942 (142.251-186.969) | 24569 (20084-29701) | 201.04 (164.336-243.03) | 1.7 (0.98 to 2.42) |
| Albania | 3282 (2811-3777) | 231.023 (197.91-265.877) | 2246 (1820-2704) | 236.928 (192.019-285.209) | -0.45 (-0.63 to -0.26) |
| Algeria | 21224 (18585-24178) | 210.045 (183.927-239.286) | 53066 (44121-63688) | 311.661 (259.126-374.043) | 1.16 (1.02 to 1.31) |
| American Samoa | 27 (23-31) | 134.339 (113.09-155.551) | 27 (22-33) | 155.691 (127.495-186.695) | 0.32 (0.18 to 0.45) |
| Andorra | 24 (20-30) | 97.864 (80.068-118.983) | 26 (21-32) | 103.443 (83.18-124.558) | 0 (-0.12 to 0.13) |
| Angola | 4030 (3347-4698) | 102.999 (85.539-120.062) | 12035 (9685-14652) | 98.919 (79.607-120.434) | -0.11 (-0.14 to -0.08) |
| Antigua and Barbuda | 56 (48-65) | 218.094 (186.63-251.925) | 91 (75-107) | 265.142 (216.716-311.26) | 0.47 (0.35 to 0.59) |
| Argentina | 11501 (9607-13502) | 94.155 (78.65-110.535) | 16358 (13175-19882) | 93.352 (75.187-113.462) | 0.08 (0 to 0.17) |
| Armenia | 3002 (2605-3446) | 208.868 (181.253-239.753) | 3057 (2520-3626) | 284.356 (234.371-337.266) | 0.63 (0.29 to 0.98) |
| Australia | 7010 (5928-8213) | 103.508 (87.53-121.27) | 8368 (6783-10191) | 96.519 (78.242-117.554) | -0.56 (-0.68 to -0.44) |
| Austria | 2867 (2338-3523) | 95.489 (77.884-117.35) | 3357 (2742-4131) | 118.935 (97.151-146.368) | 0.28 (0.15 to 0.4) |
| Azerbaijan | 6053 (5215-7031) | 190.444 (164.08-221.227) | 10859 (8844-12948) | 256.352 (208.797-305.662) | 0.36 (0.1 to 0.62) |
| Bahamas | 246 (211-285) | 208.42 (178.449-241.843) | 396 (325-473) | 256.235 (210.144-306.044) | 0.48 (0.31 to 0.64) |
| Bahrain | 926 (807-1060) | 361.225 (314.77-413.457) | 2615 (2147-3124) | 371.64 (305.097-444.017) | 0.11 (-0.07 to 0.3) |
| Bangladesh | 55672 (46743-65314) | 131.893 (110.738-154.736) | 119045 (97350-142422) | 172.996 (141.468-206.966) | 0.86 (0.78 to 0.94) |
| Barbados | 251 (215-290) | 230.106 (197.088-266.019) | 275 (225-326) | 278.117 (227.999-330.152) | 0.45 (0.31 to 0.58) |
| Belarus | 11137 (9600-12833) | 282.346 (243.386-325.331) | 9815 (8127-11724) | 334.569 (277.016-399.641) | 0.3 (0.07 to 0.52) |
| Belgium | 3903 (3263-4620) | 104.898 (87.692-124.18) | 3418 (2704-4204) | 97.587 (77.202-120.029) | -0.6 (-0.7 to -0.49) |
| Belize | 117 (100-134) | 159.3 (136.725-183.499) | 394 (321-464) | 208.614 (169.901-245.941) | 0.76 (0.7 to 0.82) |
| Benin | 1751 (1470-2059) | 102.856 (86.341-120.909) | 5815 (4652-7119) | 110.926 (88.735-135.786) | 0.25 (0.2 to 0.3) |
| Bermuda | 70 (60-81) | 273.249 (234.34-315.805) | 55 (46-65) | 314.353 (261.96-370.474) | 0.3 (0.21 to 0.39) |
| Bhutan | 348 (296-405) | 129.154 (109.911-150.174) | 641 (527-770) | 185.08 (152.077-222.234) | 1 (0.88 to 1.13) |
| Bolivia (Plurinational State of) | 3143 (2638-3626) | 127.465 (106.977-147.037) | 7309 (5942-8746) | 148.748 (120.911-177.985) | 0.54 (0.42 to 0.66) |
| Bosnia and Herzegovina | 4984 (4264-5793) | 262.443 (224.522-305.063) | 3192 (2580-3813) | 317.198 (256.407-378.968) | 0.38 (0.23 to 0.53) |
| Botswana | 508 (428-594) | 98.651 (83.076-115.457) | 1663 (1315-2001) | 156.088 (123.421-187.865) | 1.52 (1.4 to 1.63) |
| Brazil | 95554 (74789-118543) | 152.287 (119.193-188.925) | 151249 (115095-190876) | 177.418 (135.008-223.901) | 0.4 (0.34 to 0.46) |
| Brunei Darussalam | 97 (79-116) | 78.573 (64.156-94.005) | 164 (131-204) | 80.456 (63.988-99.868) | -0.33 (-0.49 to -0.17) |
| Bulgaria | 7580 (6512-8753) | 254.63 (218.763-294.023) | 6096 (4979-7319) | 320.917 (262.081-385.281) | 0.96 (0.85 to 1.07) |
| Burkina Faso | 2938 (2456-3461) | 92.153 (77.033-108.579) | 8828 (7089-10690) | 102.002 (81.911-123.525) | 0.31 (0.28 to 0.34) |
| Burundi | 1974 (1633-2319) | 95.216 (78.751-111.854) | 5464 (4429-6617) | 103.639 (83.998-125.513) | -0.03 (-0.29 to 0.22) |
| Cabo Verde | 127 (107-148) | 97.348 (82.141-113.405) | 407 (330-493) | 162.542 (131.512-196.633) | 1.24 (1.06 to 1.41) |
| Cambodia | 3101 (2574-3677) | 80.551 (66.845-95.5) | 7528 (6021-9128) | 103.926 (83.126-126.029) | 0.49 (0.27 to 0.72) |
| Cameroon | 3163 (2616-3752) | 83.178 (68.791-98.663) | 13341 (10781-16194) | 103.476 (83.622-125.601) | 0.67 (0.65 to 0.69) |
| Canada | 14073 (11893-16442) | 126.589 (106.987-147.906) | 13235 (10863-15993) | 111.575 (91.579-134.827) | -0.79 (-0.99 to -0.59) |
| Central African Republic | 915 (762-1063) | 87.882 (73.177-102.116) | 2067 (1630-2513) | 94.691 (74.671-115.121) | 0.13 (0.1 to 0.16) |
| Chad | 2271 (1903-2668) | 108.185 (90.661-127.1) | 6780 (5442-8205) | 107.734 (86.482-130.384) | 0.01 (-0.05 to 0.07) |
| Chile | 4191 (3409-5070) | 73.168 (59.518-88.528) | 6496 (5157-7976) | 91.808 (72.886-112.73) | 0.14 (-0.05 to 0.34) |
| China | 981891 (755846-1244419) | 179.132 (137.893-227.026) | 1189606 (883052-1559878) | 257.8 (191.367-338.042) | 0.92 (0.74 to 1.1) |
| Colombia | 20638 (17638-23588) | 146.848 (125.499-167.835) | 32825 (26937-39429) | 163.359 (134.056-196.222) | 0.16 (0.08 to 0.24) |
| Comoros | 171 (143-203) | 99.178 (82.809-117.668) | 399 (321-480) | 128.748 (103.55-154.838) | 0.87 (0.77 to 0.97) |
| Congo | 882 (735-1028) | 93.1 (77.633-108.517) | 2636 (2109-3129) | 118.983 (95.194-141.21) | 1 (0.92 to 1.08) |
| Cook Islands | 10 (8-11) | 127.219 (107.265-147.783) | 9 (7-11) | 154.438 (126.39-186.053) | 0.7 (0.55 to 0.84) |
| Costa Rica | 2437 (2082-2819) | 189.686 (162.097-219.417) | 4369 (3580-5197) | 229.574 (188.083-273.047) | 0.27 (0.13 to 0.42) |
| Côte d'Ivoire | 5098 (4282-5974) | 107.78 (90.518-126.301) | 3541 (2855-4306) | 283.714 (228.765-344.991) | -0.02 (-0.16 to 0.11) |
| Croatia | 4895 (4138-5720) | 269.779 (228.024-315.251) | 9294 (7608-11167) | 259.194 (212.192-311.434) | 0.22 (-0.15 to 0.58) |
| Cuba | 10203 (8748-11822) | 209.101 (179.277-242.27) | 519 (418-640) | 103.446 (83.298-127.635) | 0.2 (-0.05 to 0.45) |
| Cyprus | 254 (206-312) | 82.686 (67.054-101.557) | 10682 (8710-12750) | 361.742 (294.964-431.765) | 0.66 (0.49 to 0.84) |
| Czechia | 12889 (11081-14809) | 347.35 (298.638-399.087) | 19729 (15830-23730) | 196.141 (157.378-235.926) | 0.16 (0.07 to 0.25) |
| Democratic People's Republic of Korea | 13296 (11303-15445) | 159.409 (135.515-185.174) | 34254 (27043-41272) | 94.923 (74.94-114.371) | 0.33 (0.12 to 0.54) |
| Democratic Republic of the Congo | 13452 (11168-15725) | 93.903 (77.957-109.769) | 1419 (1133-1765) | 77.804 (62.128-96.786) | -0.13 (-0.18 to -0.08) |
| Denmark | 1613 (1275-2068) | 84.534 (66.842-108.37) | 809 (653-975) | 149.538 (120.706-180.369) | -0.49 (-0.72 to -0.27) |
| Djibouti | 179 (149-212) | 101.925 (84.784-121.083) | 61 (50-73) | 235.954 (194.483-280.967) | 1.29 (1.23 to 1.35) |
| Dominica | 55 (47-63) | 188.44 (160.72-217.259) | 10336 (8517-12316) | 227.311 (187.295-270.85) | 0.34 (0.14 to 0.54) |
| Dominican Republic | 5256 (4525-6043) | 171.097 (147.294-196.696) | 11654 (9614-14081) | 159.55 (131.617-192.772) | 0.84 (0.78 to 0.9) |
| Ecuador | 5243 (4436-6073) | 127.056 (107.511-147.172) | 118475 (99450-140417) | 280.668 (235.598-332.648) | 0.7 (0.67 to 0.74) |
| Egypt | 45758 (39771-51992) | 208.726 (181.417-237.164) | 3805 (3134-4529) | 146.799 (120.904-174.742) | 1.08 (0.94 to 1.22) |
| El Salvador | 2540 (2173-2932) | 121.585 (104.001-140.349) | 755 (606-913) | 108.606 (87.179-131.2) | 0.64 (0.57 to 0.7) |
| Equatorial Guinea | 145 (123-169) | 95.756 (81.233-111.695) | 2931 (2349-3554) | 104.664 (83.891-126.924) | 0.52 (0.39 to 0.66) |
| Eritrea | 1130 (941-1328) | 87.214 (72.649-102.442) | 1435 (1185-1718) | 362.825 (299.599-434.451) | 0.63 (0.57 to 0.69) |
| Estonia | 1869 (1624-2125) | 328.976 (285.954-374.053) | 635 (512-770) | 124.735 (100.518-151.164) | 0.39 (0.18 to 0.61) |
| Eswatini | 270 (224-320) | 89.485 (74.488-106.311) | 45234 (33533-58674) | 97.582 (72.339-126.575) | 1.03 (0.94 to 1.11) |
| Ethiopia | 17622 (13589-22220) | 96.455 (74.381-121.622) | 646 (528-769) | 181.103 (148.107-215.529) | 0.09 (-0.03 to 0.2) |
| Fiji | 501 (423-583) | 155.274 (131.191-180.741) | 1813 (1447-2229) | 108.816 (86.833-133.817) | 0.5 (0.43 to 0.58) |
| Finland | 2941 (2404-3552) | 162.028 (132.402-195.651) | 18438 (14737-22741) | 92.808 (74.181-114.471) | -1.43 (-1.78 to -1.07) |
| France | 23869 (19965-28246) | 108.492 (90.746-128.386) | 808 (649-979) | 107.783 (86.607-130.651) | -0.74 (-0.82 to -0.67) |
| Gabon | 372 (314-434) | 96.692 (81.755-112.834) | 1268 (1021-1533) | 126.752 (102.104-153.24) | 0.33 (0.3 to 0.36) |
| Gambia | 421 (353-497) | 111.674 (93.737-131.914) | 3251 (2681-3878) | 286.489 (236.319-341.755) | 0.34 (0.31 to 0.36) |
| Georgia | 5018 (4362-5720) | 235.717 (204.876-268.69) | 30744 (24708-37570) | 121.526 (97.666-148.506) | 0.31 (0.13 to 0.49) |
| Germany | 41817 (35266-49115) | 140.759 (118.708-165.322) | 19088 (15240-22998) | 133.465 (106.564-160.805) | -1.21 (-1.46 to -0.96) |
| Ghana | 6182 (5129-7197) | 107.694 (89.347-125.38) | 2574 (2056-3187) | 92.476 (73.838-114.483) | 0.58 (0.49 to 0.68) |
| Greece | 3225 (2589-3948) | 85.799 (68.863-105.023) | 22 (18-27) | 109.862 (89.011-134.649) | 0.11 (0.04 to 0.18) |
| Greenland | 28 (23-32) | 104.386 (87.953-121.162) | 93 (76-111) | 229.453 (187.637-273.932) | -0.82 (-1.2 to -0.43) |
| Grenada | 66 (57-76) | 197.553 (169.505-227.814) | 86 (70-103) | 154.61 (127.062-186.313) | 0.45 (0.36 to 0.54) |
| Guam | 79 (66-92) | 123.866 (104.346-144.577) | 8458 (6973-10188) | 124.253 (102.439-149.671) | 0.46 (0.28 to 0.64) |
| Guatemala | 3323 (2823-3891) | 112.503 (95.554-131.714) | 5541 (4425-6725) | 107.232 (85.643-130.15) | 0.53 (0.39 to 0.67) |
| Guinea | 2055 (1722-2389) | 100.049 (83.802-116.307) | 986 (788-1184) | 116.9 (93.409-140.362) | 0.15 (0.06 to 0.24) |
| Guinea-Bissau | 364 (304-426) | 98.093 (81.866-115.003) | 628 (516-751) | 202.194 (165.913-241.658) | 0.52 (0.37 to 0.66) |
| Guyana | 622 (531-714) | 182.664 (156.008-209.865) | 12443 (10083-14776) | 226.682 (183.689-269.182) | 0.13 (-0.07 to 0.33) |
| Haiti | 4589 (3873-5323) | 188.514 (159.085-218.652) | 6812 (5567-8104) | 154.975 (126.646-184.372) | 0.63 (0.44 to 0.81) |
| Honduras | 2406 (2036-2781) | 139.34 (117.907-161.065) | 8573 (6992-10281) | 311.589 (254.105-373.631) | 0.31 (0.23 to 0.4) |
| Hungary | 14489 (12465-16700) | 392.022 (337.251-451.86) | 118 (94-145) | 98.291 (78.597-120.797) | -0.42 (-0.61 to -0.22) |
| Iceland | 108 (89-129) | 104.086 (86.11-124.497) | 1219570 (918289-1594389) | 200.108 (150.674-261.609) | -0.51 (-0.64 to -0.39) |
| India | 559152 (434948-709328) | 163.961 (127.541-207.998) | 159879 (121453-205223) | 140.399 (106.655-180.219) | 0.62 (0.56 to 0.68) |
| Indonesia | 86671 (66853-110595) | 111.055 (85.661-141.708) | 129250 (96435-168951) | 372.445 (277.885-486.847) | 0.86 (0.79 to 0.92) |
| Iran (Islamic Republic of) | 47434 (38275-59729) | 218.408 (176.233-275.019) | 53540 (44061-63511) | 307.059 (252.697-364.24) | 1.65 (1.38 to 1.93) |
| Iraq | 16536 (14335-19139) | 230.138 (199.508-266.373) | 1482 (1185-1807) | 94.687 (75.717-115.466) | 1.16 (1.01 to 1.31) |
| Ireland | 1309 (1071-1575) | 95.459 (78.082-114.831) | 3078 (2447-3767) | 92.621 (73.635-113.362) | -0.11 (-0.19 to -0.03) |
| Israel | 1910 (1550-2339) | 99.948 (81.098-122.422) | 18452 (14091-23471) | 116.823 (89.212-148.6) | -0.19 (-0.26 to -0.11) |
| Italy | 25143 (19823-31390) | 117.777 (92.856-147.037) | 2991 (2454-3552) | 250.696 (205.689-297.701) | -0.15 (-0.31 to 0.02) |
| Jamaica | 1842 (1568-2143) | 187.439 (159.552-218.024) | 25933 (19735-32951) | 80.015 (60.891-101.67) | 0.66 (0.5 to 0.82) |
| Japan | 38177 (29584-47744) | 85.19 (66.014-106.536) | 16614 (13734-19890) | 309.489 (255.842-370.518) | -0.04 (-0.15 to 0.06) |
| Jordan | 3362 (2927-3833) | 218.754 (190.443-249.405) | 19744 (16049-23572) | 283.3 (230.282-338.228) | 1.13 (1.05 to 1.22) |
| Kazakhstan | 16095 (13857-18642) | 237.076 (204.109-274.599) | 27874 (21192-35465) | 128.737 (97.878-163.8) | 0.24 (0.04 to 0.45) |
| Kenya | 9425 (7303-11801) | 107.626 (83.386-134.752) | 75 (61-90) | 150.819 (122.397-180.15) | 0.64 (0.62 to 0.66) |
| Kiribati | 42 (35-49) | 138.549 (115.608-161.497) | 10406 (8786-12424) | 490.233 (413.9-585.304) | -0.07 (-0.23 to 0.09) |
| Kuwait | 3221 (2789-3716) | 381.082 (330.009-439.715) | 4808 (3917-5781) | 176.648 (143.944-212.424) | 0.67 (0.62 to 0.72) |
| Kyrgyzstan | 3322 (2865-3842) | 184.188 (158.857-213.013) | 3401 (2716-4134) | 106.02 (84.666-128.873) | -0.59 (-0.82 to -0.36) |
| Lao People's Democratic Republic | 1439 (1194-1699) | 93.144 (77.306-109.961) | 1433 (1156-1723) | 266.067 (214.626-319.882) | 0.22 (0.03 to 0.41) |
| Latvia | 2195 (1887-2540) | 230.094 (197.832-266.208) | 8373 (6923-9959) | 360.83 (298.348-429.191) | 0.14 (-0.07 to 0.35) |
| Lebanon | 2659 (2304-3055) | 230.702 (199.902-265.035) | 836 (669-1015) | 100.526 (80.378-121.962) | 1.31 (1.16 to 1.45) |
| Lesotho | 447 (374-527) | 82.811 (69.227-97.649) | 2932 (2371-3535) | 130.592 (105.607-157.452) | 0.71 (0.55 to 0.86) |
| Liberia | 1067 (899-1252) | 115.683 (97.456-135.751) | 10104 (8308-12136) | 336.743 (276.889-404.453) | 0.44 (0.19 to 0.69) |
| Libya | 3678 (3192-4223) | 218.92 (189.99-251.354) | 1856 (1497-2249) | 230.502 (185.914-279.38) | 1.78 (1.65 to 1.9) |
| Lithuania | 3046 (2603-3521) | 218.611 (186.854-252.706) | 213 (169-259) | 96.474 (76.628-117.5) | -0.22 (-0.35 to -0.09) |
| Luxembourg | 136 (111-166) | 92.355 (74.916-112.806) | 12889 (10214-15626) | 110.064 (87.22-133.43) | -0.38 (-0.53 to -0.22) |
| Madagascar | 4437 (3682-5246) | 97.953 (81.294-115.825) | 9111 (7331-11026) | 111.332 (89.582-134.741) | 0.33 (0.31 to 0.36) |
| Malawi | 3688 (3112-4351) | 98.715 (83.28-116.445) | 23492 (19217-28484) | 168.974 (138.226-204.882) | 0.44 (0.38 to 0.51) |
| Malaysia | 9244 (7767-10958) | 124.55 (104.646-147.65) | 459 (372-558) | 176.543 (143.17-214.468) | 0.99 (0.85 to 1.13) |
| Maldives | 79 (67-93) | 97.767 (81.984-114.444) | 8460 (6740-10267) | 94.986 (75.672-115.272) | 1.5 (1.11 to 1.88) |
| Mali | 2851 (2388-3319) | 95.478 (79.98-111.149) | 137 (109-170) | 102.601 (81.402-126.714) | -0.01 (-0.05 to 0.03) |
| Malta | 139 (113-171) | 100.947 (81.541-123.901) | 32 (26-39) | 135.465 (109.247-163.773) | -0.18 (-0.37 to 0.01) |
| Marshall Islands | 20 (16-23) | 113.903 (95.563-132.481) | 2232 (1805-2697) | 130.727 (105.727-157.993) | 0.75 (0.63 to 0.87) |
| Mauritania | 942 (796-1096) | 122.54 (103.521-142.6) | 634 (507-775) | 139.268 (111.448-170.254) | 0.15 (0.1 to 0.2) |
| Mauritius | 647 (535-767) | 130.249 (107.693-154.295) | 105660 (80245-137180) | 205.119 (155.781-266.309) | -0.01 (-0.11 to 0.09) |
| Mexico | 58162 (45608-72995) | 163.092 (127.89-204.686) | 55 (45-66) | 129.676 (105.129-156.073) | 0.72 (0.69 to 0.76) |
| Micronesia (Federated States of) | 50 (42-58) | 124.659 (105.348-144.653) | 9 (7-11) | 94.229 (76.28-114.826) | 0.19 (0.11 to 0.26) |
| Monaco | 9 (7-11) | 98.423 (79.269-120.435) | 3294 (2697-3990) | 261.011 (213.723-316.134) | -0.69 (-0.91 to -0.47) |
| Mongolia | 1610 (1366-1858) | 182.213 (154.605-210.284) | 715 (579-845) | 347.433 (281.342-410.877) | 0.92 (0.82 to 1.02) |
| Montenegro | 770 (656-884) | 306.755 (261.206-352.17) | 45447 (37715-54025) | 309.556 (256.887-367.98) | 0.35 (0.27 to 0.44) |
| Morocco | 25568 (22101-29329) | 245.928 (212.581-282.102) | 12724 (10203-15460) | 105.823 (84.855-128.582) | 0.65 (0.59 to 0.72) |
| Mozambique | 4946 (4138-5910) | 104.271 (87.242-124.605) | 22394 (17997-27434) | 99.608 (80.051-122.027) | 0.16 (0.08 to 0.24) |
| Myanmar | 15832 (13190-18647) | 92.224 (76.83-108.619) | 1255 (1008-1526) | 120.097 (96.414-146.056) | 0.24 (0.21 to 0.27) |
| Namibia | 585 (493-689) | 104.662 (88.192-123.244) | 7 (6-8) | 148.877 (121.37-180.837) | 0.39 (0.36 to 0.43) |
| Nauru | 6 (5-7) | 137.512 (115.36-161.128) | 19172 (15796-22966) | 142.992 (117.817-171.292) | 0.17 (0.14 to 0.21) |
| Nepal | 10306 (8799-11951) | 141.085 (120.447-163.604) | 5204 (4182-6297) | 98.546 (79.197-119.244) | 0.06 (0.02 to 0.1) |
| Netherlands | 7351 (6017-8788) | 121.911 (99.787-145.747) | 1951 (1465-2527) | 108.332 (81.342-140.295) | -1.48 (-1.81 to -1.15) |
| New Zealand | 2043 (1593-2596) | 147.915 (115.32-187.942) | 4711 (3872-5640) | 165.52 (136.03-198.169) | -1.96 (-2.25 to -1.66) |
| Nicaragua | 2027 (1738-2325) | 137.333 (117.731-157.538) | 8285 (6616-9960) | 92.909 (74.187-111.694) | 0.6 (0.49 to 0.71) |
| Niger | 2636 (2204-3080) | 94.707 (79.189-110.665) | 114183 (86924-146144) | 126.956 (96.648-162.493) | -0.24 (-0.31 to -0.17) |
| Nigeria | 38762 (30145-49111) | 113.526 (88.286-143.834) | 1 (1-1) | 154.568 (126.031-186.989) | 0.55 (0.47 to 0.62) |
| Niue | 1 (1-1) | 138.91 (116.857-162.099) | 2565 (2074-3075) | 335.439 (271.208-402.089) | 0.43 (0.35 to 0.51) |
| North Macedonia | 2265 (1936-2637) | 285.277 (243.82-332.034) | 29 (24-35) | 177.58 (145.083-212.445) | 0.4 (0.28 to 0.53) |
| Northern Mariana Islands | 39 (33-46) | 167.437 (141.071-195.842) | 1806 (1369-2300) | 101.779 (77.113-129.581) | 0.03 (-0.34 to 0.41) |
| Norway | 1843 (1440-2318) | 115.188 (90.025-144.92) | 9402 (7719-11148) | 406.239 (333.554-481.7) | -0.71 (-0.8 to -0.61) |
| Oman | 2072 (1779-2370) | 249.7 (214.459-285.688) | 215064 (167633-278933) | 217.442 (169.487-282.017) | 1.52 (1.17 to 1.87) |
| Pakistan | 72780 (57613-91459) | 178.362 (141.192-224.137) | 10 (9-13) | 177.991 (145.037-213.181) | 0.73 (0.69 to 0.77) |
| Palau | 9 (7-10) | 125.776 (104.124-146.33) | 5403 (4455-6400) | 247.442 (204.003-293.071) | 0.85 (0.66 to 1.05) |
| Palestine | 1584 (1384-1816) | 206.388 (180.322-236.594) | 2882 (2359-3472) | 174.621 (142.947-210.349) | 0.33 (0.24 to 0.42) |
| Panama | 1453 (1227-1680) | 143.661 (121.32-166.088) | 5557 (4501-6691) | 129.849 (105.166-156.355) | 0.59 (0.5 to 0.69) |
| Papua New Guinea | 1879 (1575-2223) | 113.514 (95.18-134.275) | 5305 (4290-6520) | 173.405 (140.205-213.118) | 0.39 (0.34 to 0.43) |
| Paraguay | 2315 (1932-2748) | 147.679 (123.25-175.305) | 24831 (20514-29688) | 167.09 (138.039-199.767) | 0.3 (0.17 to 0.43) |
| Peru | 10975 (9351-12658) | 123.713 (105.411-142.689) | 58175 (44504-74737) | 123.122 (94.188-158.173) | 1.15 (1.06 to 1.24) |
| Philippines | 27657 (21619-34892) | 106.705 (83.41-134.619) | 35419 (26930-45864) | 292.756 (222.59-379.086) | 0.28 (0.14 to 0.41) |
| Poland | 43645 (34527-54127) | 302.146 (239.021-374.709) | 2433 (1901-3078) | 82.438 (64.391-104.277) | 0.01 (-0.34 to 0.37) |
| Portugal | 2553 (2043-3156) | 67.424 (53.97-83.363) | 2816 (2325-3371) | 272.248 (224.83-325.9) | 0.54 (0.43 to 0.64) |
| Puerto Rico | 3322 (2877-3809) | 234.839 (203.367-269.252) | 7629 (6323-9068) | 461.675 (382.614-548.766) | 0.49 (0.46 to 0.52) |
| Qatar | 963 (830-1106) | 407.214 (351.154-467.668) | 15082 (12163-18081) | 134.518 (108.488-161.272) | -0.13 (-0.28 to 0.02) |
| Republic of Korea | 13199 (10830-15846) | 62.711 (51.455-75.286) | 12359 (9827-15371) | 77.235 (61.411-96.062) | 0.03 (-0.2 to 0.26) |
| Republic of Moldova | 4295 (3686-4961) | 246.421 (211.481-284.67) | 3862 (3121-4622) | 311.351 (251.603-372.624) | 0.69 (0.39 to 0.99) |
| Romania | 24431 (20959-27955) | 281.23 (241.267-321.801) | 3630 (2895-4408) | 100.848 (80.428-122.489) | 0.59 (0.45 to 0.73) |
| Russian Federation | 241294 (186717-307400) | 414.798 (320.977-528.437) | 16714 (13613-19914) | 310.137 (252.592-369.505) | 0.58 (0.28 to 0.89) |
| Rwanda | 2506 (2101-2957) | 91.442 (76.675-107.887) | 240090 (178562-316276) | 516.599 (384.209-680.525) | 0.22 (-0.08 to 0.53) |
| Saint Kitts and Nevis | 36 (31-42) | 207.218 (177.907-241.9) | 5752 (4604-6944) | 101.424 (81.185-122.431) | 0.46 (0.37 to 0.56) |
| Saint Lucia | 112 (96-130) | 199.114 (171.466-230.342) | 60 (49-72) | 266.052 (217.467-316.586) | 0.87 (0.84 to 0.9) |
| Saint Vincent and the Grenadines | 79 (68-92) | 172.245 (147.113-199.471) | 176 (145-208) | 266.38 (219.036-314.315) | 0.76 (0.67 to 0.85) |
| Samoa | 82 (69-96) | 123.151 (103.139-143.131) | 94 (76-112) | 226.753 (184.404-271.68) | 0.41 (0.23 to 0.59) |
| San Marino | 8 (6-10) | 83.777 (68.502-101.431) | 118 (97-144) | 147.332 (121.049-178.6) | 0.12 (-0.09 to 0.33) |
| Sao Tome and Principe | 43 (36-51) | 100.578 (84.654-117.904) | 8 (7-10) | 90.771 (72.987-111.128) | 1.16 (1.08 to 1.24) |
| Saudi Arabia | 15110 (13034-17318) | 227.382 (196.139-260.613) | 125 (100-150) | 137.111 (110.475-164.638) | 1.98 (1.89 to 2.08) |
| Senegal | 3340 (2785-3902) | 121.047 (100.921-141.404) | 78515 (64559-92874) | 423.897 (348.547-501.417) | 0.37 (0.32 to 0.41) |
| Serbia | 13707 (11867-15898) | 381.7 (330.463-442.694) | 8654 (7011-10403) | 134.213 (108.738-161.336) | 0.31 (0.21 to 0.41) |
| Seychelles | 32 (27-38) | 103.42 (86.018-121.903) | 12304 (10179-14576) | 415.087 (343.375-491.737) | 0.83 (0.75 to 0.91) |
| Sierra Leone | 1997 (1684-2335) | 125.005 (105.414-146.172) | 55 (45-68) | 144.039 (116.71-176.684) | -0.44 (-0.54 to -0.33) |
| Singapore | 1586 (1313-1875) | 105.07 (87.01-124.214) | 4460 (3602-5425) | 119.578 (96.564-145.458) | -0.04 (-0.29 to 0.21) |
| Slovakia | 5865 (5013-6809) | 286.506 (244.905-332.643) | 2122 (1768-2542) | 110.285 (91.916-132.149) | 0.24 (-0.01 to 0.5) |
| Slovenia | 2417 (2058-2818) | 315.368 (268.587-367.665) | 5086 (4158-6079) | 297.201 (242.969-355.241) | 0.25 (0.11 to 0.39) |
| Solomon Islands | 136 (113-158) | 105.785 (88.216-123.137) | 1873 (1541-2241) | 328.985 (270.69-393.53) | 0.91 (0.82 to 1) |
| Somalia | 3087 (2551-3653) | 106.472 (87.977-125.987) | 358 (285-431) | 130.788 (104.365-157.396) | 0.44 (0.05 to 0.84) |
| South Africa | 22361 (17411-28089) | 142.106 (110.644-178.502) | 8621 (6814-10371) | 103.979 (82.187-125.092) | 0.45 (0.27 to 0.63) |
| South Sudan | 2041 (1697-2396) | 88.427 (73.516-103.802) | 42677 (32461-55460) | 176.009 (133.875-228.728) | 0.72 (0.65 to 0.79) |
| Spain | 12809 (10669-15456) | 86.374 (71.946-104.225) | 11776 (9456-14442) | 94.844 (76.157-116.316) | 0.54 (0.35 to 0.72) |
| Sri Lanka | 8097 (6746-9610) | 109.504 (91.226-129.966) | 10242 (8264-12552) | 127.001 (102.477-155.641) | 0.55 (0.51 to 0.6) |
| Sudan | 16494 (14381-18831) | 216.441 (188.718-247.114) | 48805 (40385-58367) | 264.015 (218.466-315.742) | 0.66 (0.63 to 0.68) |
| Suriname | 317 (271-369) | 194.582 (166.663-226.384) | 557 (458-657) | 259.547 (213.223-306.205) | 0.66 (0.5 to 0.83) |
| Sweden | 2623 (2069-3257) | 89.357 (70.47-110.95) | 3047 (2324-3925) | 93.993 (71.696-121.08) | -0.36 (-0.62 to -0.11) |
| Switzerland | 2561 (2118-3095) | 97.196 (80.381-117.477) | 2507 (1989-3080) | 90.276 (71.643-110.936) | -0.77 (-0.94 to -0.61) |
| Syrian Arab Republic | 10092 (8781-11517) | 210.372 (183.033-240.056) | 12739 (10689-15263) | 250.466 (210.149-300.088) | 1.12 (0.94 to 1.31) |
| Taiwan (Province of China) | 18257 (15563-20990) | 197.877 (168.677-227.498) | 17972 (14634-21494) | 238.211 (193.957-284.89) | 0.6 (0.55 to 0.64) |
| Tajikistan | 3764 (3242-4341) | 177.978 (153.289-205.275) | 8409 (6732-10108) | 201.599 (161.399-242.33) | -0.01 (-0.23 to 0.21) |
| Thailand | 24309 (20074-28629) | 93.742 (77.412-110.399) | 25193 (20149-30825) | 118.835 (95.043-145.401) | 0.73 (0.63 to 0.83) |
| Timor-Leste | 290 (240-347) | 91.184 (75.384-109.084) | 491 (399-602) | 86.023 (69.921-105.301) | -0.37 (-0.41 to -0.33) |
| Togo | 1408 (1179-1647) | 102.673 (85.979-120.14) | 4109 (3269-4980) | 122.132 (97.16-148.004) | 0.53 (0.46 to 0.6) |
| Tokelau | 1 (1-1) | 111.396 (92.769-130.273) | 1 (1-1) | 139.166 (113.116-169.673) | 0.88 (0.76 to 0.99) |
| Tonga | 45 (38-52) | 120.73 (101.855-141.159) | 58 (47-70) | 148.141 (121.058-179.026) | 0.73 (0.61 to 0.86) |
| Trinidad and Tobago | 1305 (1118-1503) | 260.394 (222.995-299.877) | 1788 (1477-2108) | 359.19 (296.759-423.556) | 0.81 (0.61 to 1.02) |
| Tunisia | 7158 (6237-8201) | 208.093 (181.317-238.411) | 13293 (11095-15862) | 305.456 (254.956-364.488) | 1.14 (1.03 to 1.25) |
| Turkey | 58237 (50275-66537) | 243.477 (210.189-278.178) | 85805 (70603-101691) | 269.321 (221.608-319.186) | 0.72 (0.62 to 0.82) |
| Turkmenistan | 2698 (2323-3103) | 175.77 (151.344-202.164) | 4920 (3996-5931) | 236.544 (192.146-285.148) | 0.03 (-0.04 to 0.09) |
| Tuvalu | 5 (4-5) | 126.28 (106.139-147.034) | 7 (5-8) | 136.328 (109.317-164.433) | 0.81 (0.58 to 1.04) |
| Uganda | 5404 (4502-6364) | 84.256 (70.193-99.209) | 15055 (12007-18249) | 87.534 (69.813-106.107) | 0.01 (-0.05 to 0.08) |
| Ukraine | 54745 (42684-69077) | 288.214 (224.718-363.67) | 47746 (35584-63015) | 346.365 (258.137-457.13) | 0.56 (0.28 to 0.84) |
| United Arab Emirates | 3727 (3225-4283) | 389.926 (337.427-448.041) | 26736 (22287-31898) | 665.498 (554.772-793.996) | 1.58 (1.02 to 2.15) |
| United Kingdom | 26745 (21142-33173) | 127.969 (101.158-158.723) | 23892 (18460-30011) | 109.841 (84.869-137.971) | -1.14 (-1.37 to -0.91) |
| United Republic of Tanzania | 8087 (6738-9476) | 83.469 (69.542-97.807) | 26341 (20968-31905) | 112.889 (89.861-136.733) | 1.19 (1.12 to 1.26) |
| United States of America | 156760 (120070-198273) | 153.431 (117.521-194.062) | 150222 (111576-196151) | 134.97 (100.248-176.236) | -0.99 (-1.15 to -0.83) |
| United States Virgin Islands | 87 (75-101) | 220.257 (188.543-254.11) | 63 (52-74) | 270.897 (222.826-318.733) | 0.57 (0.52 to 0.62) |
| Uruguay | 936 (775-1114) | 82.397 (68.191-98.087) | 997 (806-1216) | 83.326 (67.365-101.666) | -0.11 (-0.2 to -0.01) |
| Uzbekistan | 14724 (12504-17121) | 171.536 (145.673-199.461) | 32712 (26874-38806) | 238.129 (195.627-282.488) | 0.85 (0.68 to 1.01) |
| Vanuatu | 83 (69-96) | 141.116 (117.924-163.217) | 197 (160-238) | 157.878 (127.999-191.295) | 0.28 (0.24 to 0.31) |
| Venezuela (Bolivarian Republic of) | 13885 (11966-15973) | 173.368 (149.4-199.438) | 19511 (16069-23101) | 208.389 (171.633-246.736) | 0.4 (0.27 to 0.52) |
| Viet Nam | 26380 (22076-31411) | 92.503 (77.41-110.145) | 51290 (41177-62446) | 133.603 (107.259-162.662) | 1.04 (0.94 to 1.14) |
| Yemen | 9737 (8460-11141) | 211.821 (184.029-242.367) | 33089 (27256-39384) | 240.475 (198.089-286.23) | 0.65 (0.43 to 0.88) |
| Zambia | 2349 (1940-2790) | 77.401 (63.926-91.943) | 7583 (6022-9194) | 93.701 (74.41-113.61) | 0.63 (0.56 to 0.7) |
| Zimbabwe | 3877 (3250-4590) | 97.791 (81.966-115.762) | 7832 (6296-9423) | 123.58 (99.349-148.681) | 0.98 (0.89 to 1.08) |
| **Incidence** |  |  |  |  |  |
| Afghanistan | 1263 (785-1798) | 40.087 (24.933-57.095) | 5806 (3699-8185) | 47.506 (30.264-66.971) | 1.48 (0.8 to 2.16) |
| Albania | 556 (337-791) | 39.154 (23.715-55.709) | 361 (218-519) | 38.093 (23.008-54.748) | -0.44 (-0.61 to -0.27) |
| Algeria | 4498 (2815-6340) | 44.512 (27.855-62.741) | 10776 (6620-15732) | 63.288 (38.882-92.397) | 1.06 (0.92 to 1.19) |
| American Samoa | 5 (3-7) | 25.16 (14.955-35.77) | 5 (3-7) | 28.657 (17.099-41.976) | 0.26 (0.14 to 0.37) |
| Andorra | 4 (2-6) | 14.449 (8.006-22.23) | 3 (2-5) | 13.681 (7.354-21.178) | -0.23 (-0.34 to -0.11) |
| Angola | 1016 (621-1469) | 25.972 (15.875-37.544) | 2952 (1730-4250) | 24.266 (14.22-34.937) | -0.22 (-0.24 to -0.2) |
| Antigua and Barbuda | 11 (7-16) | 43.169 (26.364-61.271) | 17 (11-24) | 49.66 (30.554-70.348) | 0.4 (0.31 to 0.48) |
| Argentina | 2366 (1371-3481) | 19.37 (11.221-28.5) | 3337 (1851-5021) | 19.042 (10.564-28.657) | 0.02 (-0.08 to 0.12) |
| Armenia | 561 (340-804) | 39.055 (23.631-55.927) | 542 (332-778) | 50.428 (30.888-72.328) | 0.56 (0.28 to 0.85) |
| Australia | 1396 (840-2041) | 20.615 (12.409-30.139) | 1537 (855-2446) | 17.729 (9.859-28.214) | -0.6 (-0.78 to -0.42) |
| Austria | 430 (247-647) | 14.322 (8.23-21.563) | 423 (240-648) | 14.994 (8.51-22.953) | -0.22 (-0.38 to -0.05) |
| Azerbaijan | 1199 (722-1723) | 37.74 (22.723-54.211) | 2028 (1229-2917) | 47.885 (29.012-68.854) | 0.3 (0.08 to 0.51) |
| Bahamas | 52 (32-72) | 43.846 (26.986-61.167) | 78 (48-113) | 50.708 (31.16-73.153) | 0.4 (0.26 to 0.53) |
| Bahrain | 183 (112-263) | 71.543 (43.585-102.546) | 506 (311-738) | 71.943 (44.163-104.853) | 0.07 (-0.11 to 0.24) |
| Bangladesh | 11972 (7111-17459) | 28.363 (16.846-41.362) | 23502 (14111-34625) | 34.153 (20.506-50.317) | 0.63 (0.55 to 0.7) |
| Barbados | 50 (31-71) | 45.9 (28.84-64.884) | 52 (32-74) | 52.672 (32.87-74.78) | 0.37 (0.25 to 0.48) |
| Belarus | 2055 (1265-2896) | 52.105 (32.074-73.424) | 1719 (1032-2470) | 58.593 (35.181-84.193) | 0.21 (0.07 to 0.35) |
| Belgium | 754 (471-1066) | 20.268 (12.671-28.656) | 485 (267-748) | 13.836 (7.619-21.343) | -1.45 (-1.67 to -1.23) |
| Belize | 26 (16-37) | 35.465 (22.115-50.235) | 82 (52-116) | 43.333 (27.491-61.4) | 0.61 (0.56 to 0.66) |
| Benin | 396 (229-569) | 23.258 (13.457-33.422) | 1249 (742-1795) | 23.827 (14.152-34.236) | 0.11 (0.08 to 0.14) |
| Bermuda | 13 (8-19) | 51.079 (31.959-73.763) | 10 (6-14) | 55.992 (34.619-82.184) | 0.25 (0.18 to 0.32) |
| Bhutan | 76 (46-110) | 28.122 (16.988-40.9) | 128 (77-189) | 36.926 (22.352-54.399) | 0.76 (0.66 to 0.87) |
| Bolivia (Plurinational State of) | 644 (392-922) | 26.108 (15.916-37.38) | 1468 (872-2111) | 29.866 (17.748-42.95) | 0.51 (0.39 to 0.62) |
| Bosnia and Herzegovina | 800 (493-1143) | 42.107 (25.961-60.203) | 461 (288-670) | 45.842 (28.661-66.558) | 0.14 (0.06 to 0.23) |
| Botswana | 127 (76-185) | 24.709 (14.756-36.016) | 372 (219-549) | 34.896 (20.532-51.572) | 1.15 (1.07 to 1.23) |
| Brazil | 16695 (9974-23948) | 26.607 (15.897-38.167) | 22448 (13353-32108) | 26.333 (15.664-37.663) | -0.05 (-0.13 to 0.03) |
| Brunei Darussalam | 13 (7-20) | 10.522 (5.574-15.876) | 22 (12-33) | 10.756 (5.809-16.18) | -0.06 (-0.16 to 0.04) |
| Bulgaria | 1188 (725-1669) | 39.902 (24.364-56.074) | 876 (517-1249) | 46.117 (27.205-65.756) | 0.65 (0.58 to 0.72) |
| Burkina Faso | 675 (407-966) | 21.164 (12.761-30.307) | 1914 (1128-2757) | 22.11 (13.032-31.852) | 0.16 (0.13 to 0.2) |
| Burundi | 463 (273-671) | 22.356 (13.148-32.374) | 1240 (733-1799) | 23.518 (13.912-34.126) | -0.04 (-0.24 to 0.16) |
| Cabo Verde | 27 (16-38) | 20.941 (12.618-29.245) | 77 (45-113) | 30.815 (17.964-45.129) | 0.89 (0.74 to 1.05) |
| Cambodia | 565 (332-827) | 14.67 (8.624-21.48) | 1232 (710-1792) | 17.007 (9.797-24.738) | 0.33 (0.17 to 0.49) |
| Cameroon | 751 (440-1060) | 19.743 (11.57-27.88) | 2988 (1779-4259) | 23.174 (13.799-33.034) | 0.48 (0.43 to 0.53) |
| Canada | 2774 (1593-4216) | 24.952 (14.327-37.922) | 2497 (1435-3714) | 21.047 (12.095-31.31) | -0.9 (-1.08 to -0.72) |
| Central African Republic | 263 (159-379) | 25.243 (15.227-36.387) | 570 (343-830) | 26.093 (15.705-38.026) | 0.07 (0.03 to 0.1) |
| Chad | 509 (305-735) | 24.229 (14.518-35.01) | 1450 (888-2082) | 23.045 (14.119-33.083) | -0.11 (-0.14 to -0.08) |
| Chile | 502 (265-774) | 8.768 (4.626-13.507) | 723 (407-1092) | 10.215 (5.759-15.427) | 0.23 (0.08 to 0.38) |
| China | 179913 (111736-254408) | 32.822 (20.385-46.413) | 214550 (131074-307879) | 46.495 (28.405-66.721) | 0.98 (0.76 to 1.19) |
| Colombia | 4394 (2713-6316) | 31.262 (19.303-44.938) | 6817 (4114-9812) | 33.926 (20.473-48.829) | 0.13 (0.06 to 0.2) |
| Comoros | 36 (21-51) | 20.607 (12.279-29.614) | 78 (46-113) | 25.197 (14.816-36.41) | 0.68 (0.6 to 0.76) |
| Congo | 228 (137-332) | 24.074 (14.472-35.032) | 632 (381-914) | 28.539 (17.215-41.254) | 0.73 (0.66 to 0.8) |
| Cook Islands | 2 (1-3) | 23.549 (14.091-33.965) | 2 (1-2) | 27.546 (16.71-39.439) | 0.51 (0.39 to 0.64) |
| Costa Rica | 474 (288-674) | 36.927 (22.387-52.49) | 803 (497-1163) | 42.196 (26.1-61.104) | 0.23 (0.12 to 0.35) |
| Côte d'Ivoire | 1167 (701-1675) | 24.674 (14.817-35.416) | 444 (275-631) | 35.592 (22.029-50.559) | -0.1 (-0.21 to 0.01) |
| Croatia | 671 (401-968) | 36.969 (22.078-53.357) | 1782 (1100-2554) | 49.701 (30.684-71.226) | 0.17 (-0.13 to 0.48) |
| Cuba | 2040 (1272-2896) | 41.803 (26.059-59.345) | 48 (25-80) | 9.657 (4.935-15.973) | -0.1 (-0.41 to 0.2) |
| Cyprus | 27 (15-43) | 8.826 (4.833-13.879) | 1694 (1079-2412) | 57.352 (36.536-81.689) | 0.52 (0.41 to 0.62) |
| Czechia | 2035 (1311-2818) | 54.832 (35.332-75.944) | 3823 (2277-5526) | 38.01 (22.637-54.944) | 0.44 (0.24 to 0.64) |
| Democratic People's Republic of Korea | 2673 (1644-3846) | 32.047 (19.704-46.115) | 8755 (5105-12930) | 24.261 (14.146-35.832) | 0.31 (0.14 to 0.47) |
| Democratic Republic of the Congo | 3455 (2060-4989) | 24.12 (14.377-34.825) | 172 (92-267) | 9.428 (5.06-14.636) | -0.12 (-0.19 to -0.06) |
| Denmark | 114 (79-156) | 5.967 (4.159-8.182) | 158 (97-231) | 29.162 (17.855-42.645) | 2.01 (1.37 to 2.65) |
| Djibouti | 38 (22-54) | 21.732 (12.81-31.002) | 12 (8-17) | 47.495 (30.344-66.705) | 1.04 (1 to 1.08) |
| Dominica | 12 (7-16) | 40.078 (25.153-56.349) | 2094 (1309-2959) | 46.054 (28.775-65.063) | 0.28 (0.13 to 0.43) |
| Dominican Republic | 1128 (710-1596) | 36.733 (23.112-51.953) | 2299 (1385-3314) | 31.471 (18.962-45.364) | 0.73 (0.69 to 0.77) |
| Ecuador | 1064 (636-1514) | 25.795 (15.404-36.683) | 24177 (14959-34662) | 57.276 (35.438-82.115) | 0.66 (0.65 to 0.68) |
| Egypt | 9908 (6169-14219) | 45.194 (28.142-64.859) | 816 (488-1169) | 31.467 (18.825-45.087) | 0.9 (0.73 to 1.06) |
| El Salvador | 548 (337-780) | 26.248 (16.128-37.351) | 178 (108-255) | 25.604 (15.457-36.686) | 0.63 (0.55 to 0.7) |
| Equatorial Guinea | 37 (23-55) | 24.821 (14.927-36.39) | 653 (386-944) | 23.332 (13.782-33.712) | 0.14 (0.01 to 0.28) |
| Eritrea | 270 (163-382) | 20.831 (12.563-29.48) | 280 (171-404) | 70.762 (43.285-102.138) | 0.47 (0.42 to 0.52) |
| Estonia | 369 (270-479) | 64.946 (47.598-84.334) | 148 (89-216) | 29.143 (17.42-42.488) | 0.51 (0.15 to 0.86) |
| Eswatini | 69 (41-99) | 22.95 (13.506-32.994) | 10413 (6128-14945) | 22.464 (13.219-32.241) | 0.77 (0.7 to 0.85) |
| Ethiopia | 4280 (2598-6126) | 23.425 (14.222-33.533) | 117 (69-171) | 32.687 (19.359-47.915) | -0.11 (-0.18 to -0.03) |
| Fiji | 93 (56-134) | 28.819 (17.422-41.439) | 269 (153-414) | 16.175 (9.167-24.828) | 0.43 (0.36 to 0.49) |
| Finland | 641 (436-899) | 35.327 (24.036-49.498) | 2847 (1658-4507) | 14.331 (8.343-22.688) | -2.19 (-2.99 to -1.38) |
| France | 4373 (2983-6042) | 19.878 (13.561-27.463) | 190 (113-273) | 25.378 (15.06-36.372) | -1.32 (-1.46 to -1.18) |
| Gabon | 91 (54-133) | 23.557 (14.117-34.54) | 263 (159-376) | 26.292 (15.882-37.562) | 0.21 (0.18 to 0.25) |
| Gambia | 93 (56-135) | 24.545 (14.914-35.743) | 595 (362-864) | 52.416 (31.926-76.109) | 0.21 (0.18 to 0.24) |
| Georgia | 940 (572-1356) | 44.141 (26.849-63.683) | 4768 (2778-7231) | 18.848 (10.979-28.582) | 0.21 (0.05 to 0.36) |
| Germany | 7714 (5465-10293) | 25.967 (18.394-34.645) | 4016 (2369-5763) | 28.082 (16.562-40.293) | -1.5 (-1.66 to -1.34) |
| Ghana | 1428 (858-2082) | 24.878 (14.943-36.276) | 282 (152-445) | 10.14 (5.447-15.983) | 0.38 (0.35 to 0.42) |
| Greece | 379 (204-583) | 10.084 (5.425-15.517) | 5 (3-7) | 22.842 (12.996-33.656) | 0.31 (0.16 to 0.46) |
| Greenland | 7 (4-10) | 24.658 (14.037-36.669) | 18 (12-26) | 45.614 (29.126-64.269) | -1.08 (-1.43 to -0.72) |
| Grenada | 14 (9-20) | 41.564 (26.457-58.671) | 16 (9-23) | 28.504 (16.817-40.758) | 0.38 (0.29 to 0.47) |
| Guam | 15 (9-21) | 23.532 (13.496-33.786) | 1948 (1172-2772) | 28.623 (17.223-40.721) | 0.34 (0.19 to 0.49) |
| Guatemala | 733 (441-1040) | 24.805 (14.924-35.224) | 1233 (728-1766) | 23.866 (14.091-34.185) | 0.68 (0.55 to 0.81) |
| Guinea | 479 (272-699) | 23.314 (13.256-34.046) | 223 (133-319) | 26.429 (15.776-37.809) | 0.07 (0.01 to 0.13) |
| Guinea-Bissau | 88 (54-127) | 23.827 (14.513-34.203) | 137 (85-192) | 44.035 (27.319-61.65) | 0.37 (0.27 to 0.47) |
| Guyana | 144 (91-203) | 42.428 (26.592-59.721) | 2766 (1701-3961) | 50.382 (30.98-72.162) | 0.04 (-0.13 to 0.21) |
| Haiti | 1081 (669-1529) | 44.422 (27.477-62.813) | 1441 (869-2073) | 32.793 (19.765-47.159) | 0.55 (0.39 to 0.71) |
| Honduras | 528 (324-766) | 30.611 (18.742-44.383) | 1210 (739-1709) | 43.964 (26.841-62.106) | 0.26 (0.2 to 0.32) |
| Hungary | 2075 (1291-2969) | 56.136 (34.923-80.338) | 18 (10-28) | 14.847 (8.351-23.266) | -0.56 (-0.68 to -0.44) |
| Iceland | 19 (11-28) | 18.191 (10.938-27.219) | 267596 (165268-379568) | 43.907 (27.117-62.28) | -0.78 (-0.92 to -0.64) |
| India | 129594 (79612-185897) | 38.001 (23.345-54.511) | 25585 (14963-36631) | 22.468 (13.14-32.168) | 0.57 (0.5 to 0.65) |
| Indonesia | 14924 (8707-21358) | 19.122 (11.156-27.367) | 26476 (16249-38625) | 76.292 (46.824-111.301) | 0.73 (0.63 to 0.82) |
| Iran (Islamic Republic of) | 10555 (6583-14967) | 48.598 (30.311-68.914) | 10778 (6641-15279) | 61.815 (38.087-87.629) | 1.45 (1.17 to 1.73) |
| Iraq | 3584 (2245-5127) | 49.874 (31.24-71.36) | 194 (106-305) | 12.411 (6.785-19.496) | 0.91 (0.76 to 1.07) |
| Ireland | 186 (105-291) | 13.575 (7.648-21.235) | 370 (208-574) | 11.123 (6.252-17.286) | -0.24 (-0.3 to -0.18) |
| Israel | 239 (136-364) | 12.525 (7.104-19.053) | 2853 (1649-4246) | 18.061 (10.441-26.883) | -0.23 (-0.32 to -0.14) |
| Italy | 4131 (2331-6225) | 19.35 (10.92-29.161) | 573 (352-806) | 48.019 (29.464-67.565) | -0.29 (-0.43 to -0.14) |
| Jamaica | 374 (237-524) | 38.015 (24.125-53.335) | 3324 (1727-5037) | 10.256 (5.328-15.541) | 0.57 (0.45 to 0.69) |
| Japan | 4527 (2418-6865) | 10.101 (5.396-15.318) | 3233 (2020-4618) | 60.231 (37.633-86.016) | 0.28 (0.17 to 0.4) |
| Jordan | 679 (421-971) | 44.204 (27.408-63.163) | 3688 (2269-5230) | 52.915 (32.552-75.041) | 1.03 (0.92 to 1.14) |
| Kazakhstan | 3161 (1956-4470) | 46.561 (28.811-65.835) | 5728 (3476-8170) | 26.453 (16.056-37.732) | 0 (-0.16 to 0.17) |
| Kenya | 2048 (1246-2906) | 23.385 (14.226-33.181) | 15 (9-22) | 30.862 (18.287-44.542) | 0.49 (0.45 to 0.52) |
| Kiribati | 9 (5-13) | 28.656 (17.198-41.032) | 1877 (1147-2719) | 88.423 (54.044-128.1) | -0.02 (-0.14 to 0.09) |
| Kuwait | 612 (378-892) | 72.446 (44.732-105.538) | 1012 (605-1442) | 37.2 (22.213-52.972) | 0.57 (0.51 to 0.62) |
| Kyrgyzstan | 677 (409-955) | 37.52 (22.67-52.922) | 561 (317-817) | 17.479 (9.882-25.474) | -0.44 (-0.64 to -0.25) |
| Lao People's Democratic Republic | 260 (152-376) | 16.843 (9.852-24.359) | 219 (132-314) | 40.662 (24.572-58.246) | 0.02 (-0.11 to 0.16) |
| Latvia | 358 (212-508) | 37.482 (22.222-53.294) | 1623 (1002-2359) | 69.968 (43.201-101.647) | -0.01 (-0.21 to 0.2) |
| Lebanon | 547 (337-789) | 47.437 (29.245-68.467) | 220 (128-320) | 26.388 (15.429-38.475) | 1.18 (1.06 to 1.3) |
| Lesotho | 126 (74-182) | 23.285 (13.678-33.743) | 619 (374-889) | 27.549 (16.647-39.58) | 0.5 (0.39 to 0.62) |
| Liberia | 238 (142-345) | 25.77 (15.395-37.378) | 2019 (1241-2907) | 67.283 (41.375-96.89) | 0.28 (0.09 to 0.47) |
| Libya | 752 (470-1072) | 44.738 (27.964-63.802) | 308 (180-438) | 38.2 (22.412-54.364) | 1.74 (1.62 to 1.86) |
| Lithuania | 560 (362-761) | 40.207 (25.956-54.633) | 22 (11-35) | 10.03 (5.212-15.883) | -0.35 (-0.5 to -0.2) |
| Luxembourg | 16 (9-25) | 10.619 (5.84-16.767) | 2735 (1639-3884) | 23.358 (13.992-33.166) | -0.7 (-0.98 to -0.41) |
| Madagascar | 985 (582-1419) | 21.74 (12.847-31.32) | 1985 (1196-2843) | 24.256 (14.619-34.746) | 0.22 (0.18 to 0.27) |
| Malawi | 861 (517-1230) | 23.048 (13.844-32.922) | 3104 (1843-4493) | 22.324 (13.26-32.317) | 0.26 (0.2 to 0.33) |
| Malaysia | 1366 (788-1980) | 18.398 (10.611-26.676) | 62 (36-92) | 23.85 (13.792-35.507) | 0.85 (0.72 to 0.98) |
| Maldives | 12 (7-18) | 14.973 (8.611-21.778) | 1958 (1199-2802) | 21.987 (13.465-31.458) | 1.22 (0.87 to 1.57) |
| Mali | 689 (406-978) | 23.082 (13.603-32.769) | 14 (7-22) | 10.371 (5.532-16.484) | -0.12 (-0.15 to -0.1) |
| Malta | 15 (9-25) | 11.161 (6.25-17.777) | 7 (4-10) | 28.025 (16.712-40.631) | -0.08 (-0.35 to 0.18) |
| Marshall Islands | 4 (3-6) | 23.851 (14.666-34.071) | 443 (271-638) | 25.962 (15.897-37.383) | 0.67 (0.56 to 0.78) |
| Mauritania | 198 (118-286) | 25.801 (15.401-37.157) | 87 (49-127) | 19.037 (10.864-27.805) | 0.01 (-0.02 to 0.04) |
| Mauritius | 94 (54-136) | 18.895 (10.86-27.393) | 21076 (12912-30132) | 40.915 (25.066-58.496) | -0.13 (-0.22 to -0.04) |
| Mexico | 12155 (7502-17170) | 34.085 (21.037-48.147) | 11 (7-17) | 26.797 (15.978-39.073) | 0.64 (0.59 to 0.68) |
| Micronesia (Federated States of) | 10 (6-15) | 25.867 (15.624-37.036) | 1 (1-2) | 12.277 (6.796-19.516) | 0.18 (0.11 to 0.25) |
| Monaco | 1 (1-2) | 14.543 (8.212-22.715) | 646 (395-935) | 51.214 (31.258-74.093) | -0.93 (-1.14 to -0.71) |
| Mongolia | 346 (211-501) | 39.157 (23.883-56.744) | 100 (63-142) | 48.759 (30.444-69.191) | 0.68 (0.6 to 0.76) |
| Montenegro | 117 (71-166) | 46.738 (28.46-66.219) | 8912 (5521-12720) | 60.7 (37.606-86.642) | 0.15 (0.09 to 0.21) |
| Morocco | 5228 (3245-7483) | 50.282 (31.214-71.978) | 2877 (1729-4059) | 23.925 (14.383-33.762) | 0.59 (0.53 to 0.64) |
| Mozambique | 1185 (708-1711) | 24.983 (14.925-36.069) | 3830 (2229-5568) | 17.035 (9.913-24.767) | -0.02 (-0.09 to 0.04) |
| Myanmar | 2980 (1732-4210) | 17.358 (10.089-24.522) | 286 (172-415) | 27.363 (16.494-39.679) | -0.03 (-0.09 to 0.03) |
| Namibia | 140 (85-202) | 24.998 (15.111-36.139) | 1 (1-2) | 29.453 (17.516-43.023) | 0.24 (0.21 to 0.27) |
| Nauru | 1 (1-2) | 27.398 (16.333-39.172) | 4045 (2439-5883) | 30.171 (18.19-43.88) | 0.18 (0.15 to 0.21) |
| Nepal | 2273 (1371-3277) | 31.116 (18.772-44.859) | 780 (447-1219) | 14.77 (8.462-23.087) | -0.13 (-0.18 to -0.08) |
| Netherlands | 1116 (661-1699) | 18.51 (10.964-28.176) | 325 (211-471) | 18.034 (11.696-26.128) | -2.02 (-2.61 to -1.42) |
| New Zealand | 414 (235-631) | 29.933 (17.01-45.688) | 952 (575-1362) | 33.438 (20.197-47.838) | -2.83 (-3.32 to -2.34) |
| Nicaragua | 419 (256-603) | 28.418 (17.342-40.847) | 1877 (1115-2684) | 21.044 (12.501-30.1) | 0.56 (0.46 to 0.65) |
| Niger | 617 (359-876) | 22.168 (12.9-31.483) | 24248 (14673-34554) | 26.96 (16.315-38.419) | -0.27 (-0.33 to -0.2) |
| Nigeria | 8667 (5276-12342) | 25.383 (15.453-36.146) | 0 (0-0) | 28.563 (17.325-41.139) | 0.35 (0.29 to 0.42) |
| Niue | 0 (0-0) | 26.043 (15.815-36.992) | 365 (226-522) | 47.689 (29.496-68.229) | 0.33 (0.26 to 0.41) |
| North Macedonia | 348 (216-489) | 43.884 (27.158-61.567) | 5 (3-7) | 31.09 (18.532-44.928) | 0.23 (0.14 to 0.32) |
| Northern Mariana Islands | 7 (4-10) | 29.302 (17.304-42.918) | 277 (153-416) | 15.63 (8.647-23.413) | -0.01 (-0.34 to 0.32) |
| Norway | 299 (169-448) | 18.674 (10.555-27.974) | 1870 (1126-2731) | 80.812 (48.663-118.022) | -0.79 (-0.88 to -0.71) |
| Oman | 447 (270-647) | 53.855 (32.563-78.039) | 47229 (29812-66302) | 47.751 (30.142-67.035) | 1.32 (1 to 1.65) |
| Pakistan | 16099 (9977-22681) | 39.453 (24.451-55.583) | 2 (1-3) | 33.778 (19.96-49.433) | 0.91 (0.79 to 1.02) |
| Palau | 2 (1-2) | 24.725 (14.456-35.523) | 1080 (659-1554) | 49.477 (30.158-71.149) | 0.74 (0.57 to 0.91) |
| Palestine | 329 (203-465) | 42.916 (26.458-60.651) | 571 (345-834) | 34.576 (20.91-50.502) | 0.24 (0.15 to 0.33) |
| Panama | 305 (186-439) | 30.139 (18.356-43.398) | 1126 (673-1615) | 26.309 (15.728-37.744) | 0.48 (0.39 to 0.57) |
| Papua New Guinea | 386 (233-547) | 23.303 (14.056-33.047) | 739 (442-1062) | 24.145 (14.463-34.726) | 0.33 (0.29 to 0.37) |
| Paraguay | 341 (203-493) | 21.778 (12.954-31.435) | 4703 (2815-6804) | 31.645 (18.942-45.782) | 0.2 (0.1 to 0.3) |
| Peru | 2151 (1286-3091) | 24.249 (14.496-34.847) | 8528 (4955-12288) | 18.049 (10.487-26.005) | 0.99 (0.9 to 1.07) |
| Philippines | 4471 (2596-6369) | 17.249 (10.017-24.573) | 4232 (2598-6018) | 34.977 (21.476-49.74) | 0.14 (0.04 to 0.24) |
| Poland | 8571 (4886-12373) | 59.335 (33.825-85.654) | 245 (133-395) | 8.31 (4.513-13.393) | -1.69 (-2.07 to -1.3) |
| Portugal | 299 (162-474) | 7.89 (4.291-12.507) | 529 (329-760) | 51.19 (31.84-73.513) | 0.33 (0.23 to 0.42) |
| Puerto Rico | 656 (416-928) | 46.399 (29.389-65.579) | 1466 (881-2154) | 88.728 (53.337-130.324) | 0.39 (0.36 to 0.43) |
| Qatar | 187 (115-273) | 78.927 (48.574-115.524) | 3210 (1940-4675) | 28.627 (17.299-41.696) | -0.12 (-0.26 to 0.02) |
| Republic of Korea | 1851 (979-2798) | 8.796 (4.652-13.294) | 1381 (715-2184) | 8.633 (4.471-13.652) | -0.69 (-0.95 to -0.44) |
| Republic of Moldova | 800 (489-1145) | 45.919 (28.078-65.698) | 698 (414-1016) | 56.257 (33.372-81.911) | 0.69 (0.47 to 0.91) |
| Romania | 3740 (2329-5272) | 43.055 (26.806-60.689) | 781 (460-1125) | 21.702 (12.785-31.257) | 0.21 (0.15 to 0.27) |
| Russian Federation | 49083 (30916-68753) | 84.377 (53.146-118.19) | 2472 (1516-3528) | 45.871 (28.13-65.46) | 0.56 (0.4 to 0.72) |
| Rwanda | 582 (344-845) | 21.249 (12.567-30.817) | 46935 (29401-65712) | 100.989 (63.262-141.392) | 0.07 (-0.16 to 0.31) |
| Saint Kitts and Nevis | 7 (5-11) | 43.25 (27.064-61.163) | 1272 (719-1852) | 22.43 (12.686-32.66) | 0.37 (0.29 to 0.45) |
| Saint Lucia | 23 (15-33) | 41.444 (26.361-58.367) | 12 (7-17) | 52.015 (32.668-74.148) | 0.76 (0.75 to 0.78) |
| Saint Vincent and the Grenadines | 17 (11-24) | 37.819 (23.919-53.143) | 34 (21-49) | 52.089 (32.172-73.619) | 0.63 (0.56 to 0.71) |
| Samoa | 16 (10-23) | 23.885 (14.639-33.638) | 19 (12-27) | 46.16 (28.39-66.429) | 0.32 (0.16 to 0.48) |
| San Marino | 1 (1-2) | 12.455 (7.047-19.198) | 22 (13-32) | 27.775 (16.711-39.806) | -0.16 (-0.34 to 0.02) |
| Sao Tome and Principe | 9 (6-13) | 21.307 (12.991-30.49) | 1 (1-2) | 11.792 (6.442-18.666) | 0.85 (0.8 to 0.91) |
| Saudi Arabia | 3268 (2044-4664) | 49.185 (30.761-70.184) | 24 (14-35) | 26.518 (15.788-38.392) | 1.8 (1.72 to 1.87) |
| Senegal | 714 (436-1017) | 25.861 (15.803-36.842) | 15547 (9634-23037) | 83.938 (52.013-124.376) | 0.21 (0.15 to 0.27) |
| Serbia | 2265 (1508-3140) | 63.073 (41.982-87.429) | 1752 (1063-2513) | 27.179 (16.486-38.979) | 0.05 (-0.05 to 0.16) |
| Seychelles | 5 (3-7) | 16.22 (9.33-23.59) | 1944 (1218-2700) | 65.595 (41.078-91.093) | 0.67 (0.6 to 0.73) |
| Sierra Leone | 445 (264-644) | 27.846 (16.535-40.31) | 8 (5-12) | 20.726 (11.773-30.793) | -0.45 (-0.53 to -0.38) |
| Singapore | 256 (143-375) | 16.968 (9.472-24.871) | 959 (582-1366) | 25.703 (15.609-36.612) | 0 (-0.29 to 0.3) |
| Slovakia | 884 (548-1263) | 43.184 (26.778-61.693) | 316 (177-476) | 16.439 (9.181-24.746) | 0.08 (-0.13 to 0.3) |
| Slovenia | 348 (219-485) | 45.388 (28.544-63.299) | 711 (426-1005) | 41.53 (24.882-58.706) | 0.01 (-0.18 to 0.2) |
| Solomon Islands | 29 (18-42) | 22.992 (13.93-32.472) | 256 (157-363) | 44.907 (27.507-63.664) | 0.82 (0.71 to 0.93) |
| Somalia | 725 (437-1047) | 25.016 (15.086-36.097) | 75 (45-107) | 27.431 (16.337-39.125) | 0.36 (0.06 to 0.66) |
| South Africa | 5710 (3518-8086) | 36.285 (22.354-51.388) | 1979 (1205-2847) | 23.867 (14.53-34.336) | 0.26 (0.14 to 0.38) |
| South Sudan | 460 (273-660) | 19.923 (11.831-28.608) | 10154 (6117-14579) | 41.876 (25.227-60.126) | 0.62 (0.61 to 0.62) |
| Spain | 2119 (1294-3085) | 14.286 (8.729-20.8) | 1754 (963-2687) | 14.13 (7.758-21.642) | 0.41 (0.12 to 0.71) |
| Sri Lanka | 1282 (756-1872) | 17.331 (10.22-25.31) | 1462 (856-2108) | 18.133 (10.612-26.137) | 0.28 (0.22 to 0.35) |
| Sudan | 3718 (2322-5330) | 48.785 (30.472-69.936) | 10265 (6410-14414) | 55.532 (34.673-77.977) | 0.49 (0.45 to 0.54) |
| Suriname | 68 (43-97) | 41.636 (26.34-59.275) | 110 (69-155) | 51.107 (31.927-72.418) | 0.54 (0.39 to 0.68) |
| Sweden | 376 (303-458) | 12.81 (10.315-15.606) | 526 (280-809) | 16.23 (8.653-24.955) | -1.06 (-1.68 to -0.44) |
| Switzerland | 423 (269-602) | 16.059 (10.191-22.853) | 334 (180-523) | 12.017 (6.489-18.843) | -1.06 (-1.16 to -0.96) |
| Syrian Arab Republic | 2024 (1289-2880) | 42.19 (26.87-60.024) | 2468 (1530-3621) | 48.52 (30.076-71.196) | 1.05 (0.83 to 1.28) |
| Taiwan (Province of China) | 3365 (2038-4850) | 36.469 (22.093-52.565) | 3153 (1925-4537) | 41.784 (25.52-60.136) | 0.48 (0.44 to 0.51) |
| Tajikistan | 748 (463-1074) | 35.36 (21.872-50.785) | 1703 (1038-2438) | 40.819 (24.877-58.455) | 0.09 (-0.09 to 0.28) |
| Thailand | 4066 (2338-5851) | 15.681 (9.015-22.564) | 3849 (2218-5733) | 18.157 (10.465-27.045) | 0.5 (0.39 to 0.6) |
| Timor-Leste | 49 (29-72) | 15.537 (8.959-22.773) | 79 (45-113) | 13.825 (7.926-19.845) | -0.5 (-0.53 to -0.47) |
| Togo | 325 (197-461) | 23.68 (14.345-33.599) | 888 (523-1291) | 26.397 (15.531-38.383) | 0.36 (0.29 to 0.44) |
| Tokelau | 0 (0-0) | 22.165 (12.652-32.179) | 0 (0-0) | 26.966 (16.214-38.788) | 0.72 (0.63 to 0.8) |
| Tonga | 8 (5-12) | 22.532 (13.752-31.804) | 10 (6-15) | 26.81 (16.073-38.089) | 0.59 (0.48 to 0.71) |
| Trinidad and Tobago | 260 (161-370) | 51.847 (32.197-73.87) | 324 (202-459) | 65.163 (40.548-92.252) | 0.66 (0.48 to 0.84) |
| Tunisia | 1503 (922-2146) | 43.704 (26.816-62.395) | 2684 (1647-3941) | 61.665 (37.841-90.551) | 1.08 (0.98 to 1.18) |
| Turkey | 12228 (7581-17433) | 51.124 (31.694-72.883) | 17570 (10795-25560) | 55.147 (33.883-80.226) | 0.58 (0.49 to 0.66) |
| Turkmenistan | 560 (344-800) | 36.462 (22.398-52.107) | 964 (589-1381) | 46.339 (28.342-66.417) | 0.04 (-0.01 to 0.09) |
| Tuvalu | 1 (1-1) | 25.688 (15.06-37.341) | 1 (1-2) | 27.569 (16.837-39.764) | 1.12 (0.61 to 1.63) |
| Uganda | 1309 (785-1845) | 20.407 (12.232-28.76) | 3483 (2102-4984) | 20.254 (12.222-28.981) | -0.11 (-0.18 to -0.05) |
| Ukraine | 10549 (6526-14829) | 55.535 (34.358-78.07) | 9199 (5714-13155) | 66.735 (41.448-95.427) | 0.89 (0.62 to 1.15) |
| United Arab Emirates | 764 (466-1130) | 79.941 (48.787-118.185) | 5067 (2888-7806) | 126.125 (71.883-194.315) | 1.49 (0.99 to 1.99) |
| United Kingdom | 3584 (1941-5516) | 17.148 (9.285-26.391) | 3689 (2114-5548) | 16.958 (9.718-25.507) | -0.63 (-0.86 to -0.41) |
| United Republic of Tanzania | 1960 (1164-2824) | 20.228 (12.013-29.149) | 5815 (3529-8430) | 24.922 (15.125-36.129) | 0.89 (0.83 to 0.96) |
| United States of America | 34326 (18142-53437) | 33.597 (17.756-52.302) | 25786 (15716-36634) | 23.168 (14.121-32.915) | -2.02 (-2.27 to -1.76) |
| United States Virgin Islands | 18 (11-25) | 44.347 (27.805-63.44) | 12 (7-17) | 51.706 (32.185-73.83) | 0.5 (0.47 to 0.53) |
| Uruguay | 148 (81-219) | 13.002 (7.168-19.272) | 159 (86-240) | 13.272 (7.203-20.086) | 0.01 (-0.03 to 0.06) |
| Uzbekistan | 2950 (1809-4202) | 34.37 (21.079-48.952) | 6291 (3833-9102) | 45.797 (27.905-66.261) | 0.82 (0.69 to 0.95) |
| Vanuatu | 16 (10-24) | 28.011 (17.003-40.259) | 38 (23-55) | 30.805 (18.429-44.412) | 0.27 (0.24 to 0.3) |
| Venezuela (Bolivarian Republic of) | 2871 (1761-4062) | 35.846 (21.992-50.721) | 3821 (2340-5592) | 40.812 (24.998-59.727) | 0.33 (0.23 to 0.43) |
| Viet Nam | 4342 (2527-6281) | 15.224 (8.861-22.025) | 7448 (4285-11129) | 19.401 (11.162-28.988) | 0.76 (0.7 to 0.83) |
| Yemen | 2241 (1382-3225) | 48.744 (30.065-70.146) | 7324 (4557-10516) | 53.225 (33.12-76.427) | 0.59 (0.38 to 0.81) |
| Zambia | 603 (359-868) | 19.869 (11.83-28.618) | 1810 (1085-2575) | 22.365 (13.408-31.823) | 0.36 (0.34 to 0.38) |
| Zimbabwe | 958 (576-1363) | 24.167 (14.541-34.378) | 1848 (1089-2682) | 29.155 (17.186-42.323) | 0.83 (0.74 to 0.93) |
| **Deaths** |  |  |  |  |  |
| Afghanistan | 470 (321-646) | 14.938 (10.192-20.52) | 1559 (1120-2180) | 12.754 (9.161-17.837) | -0.02 (-0.55 to 0.51) |
| Albania | 61 (52-71) | 4.31 (3.682-5.032) | 32 (26-40) | 3.393 (2.746-4.221) | -1.29 (-1.69 to -0.89) |
| Algeria | 1239 (982-1564) | 12.258 (9.722-15.477) | 1290 (977-1656) | 7.578 (5.74-9.725) | -2.12 (-2.44 to -1.8) |
| American Samoa | 2 (2-3) | 10.084 (7.965-12.778) | 2 (2-3) | 13.437 (9.881-17.243) | 1.08 (1 to 1.15) |
| Andorra | 1 (0-1) | 2.222 (1.616-3.045) | 0 (0-0) | 1.033 (0.666-1.412) | -2.23 (-2.54 to -1.92) |
| Angola | 115 (79-153) | 2.927 (2.029-3.909) | 359 (246-497) | 2.949 (2.023-4.084) | 0.25 (0.07 to 0.43) |
| Antigua and Barbuda | 1 (1-1) | 4.244 (3.821-4.743) | 0 (0-0) | 0.999 (0.82-1.217) | -3.74 (-4.26 to -3.21) |
| Argentina | 735 (678-792) | 6.02 (5.551-6.486) | 373 (340-409) | 2.131 (1.938-2.331) | -3.1 (-3.63 to -2.56) |
| Armenia | 139 (121-155) | 9.646 (8.399-10.775) | 67 (58-76) | 6.222 (5.393-7.076) | -3.05 (-3.59 to -2.51) |
| Australia | 212 (194-231) | 3.128 (2.87-3.411) | 112 (100-126) | 1.29 (1.155-1.457) | -3.09 (-3.47 to -2.72) |
| Austria | 100 (92-109) | 3.325 (3.072-3.618) | 29 (26-31) | 1.019 (0.931-1.113) | -4.72 (-5.11 to -4.33) |
| Azerbaijan | 448 (383-520) | 14.084 (12.06-16.351) | 346 (275-428) | 8.165 (6.485-10.093) | -3.41 (-3.88 to -2.93) |
| Bahamas | 7 (7-8) | 6.206 (5.538-6.901) | 5 (4-7) | 3.435 (2.655-4.43) | -2.61 (-2.87 to -2.35) |
| Bahrain | 32 (28-36) | 12.398 (10.883-14.02) | 46 (39-55) | 6.582 (5.475-7.789) | -2.72 (-3.18 to -2.25) |
| Bangladesh | 3217 (2576-4046) | 7.621 (6.103-9.586) | 4413 (3221-5748) | 6.413 (4.681-8.353) | -0.5 (-0.84 to -0.16) |
| Barbados | 4 (4-5) | 3.728 (3.34-4.151) | 2 (1-2) | 1.547 (1.162-2.006) | -3.26 (-3.61 to -2.91) |
| Belarus | 452 (401-500) | 11.459 (10.178-12.684) | 313 (254-378) | 10.677 (8.648-12.877) | -1.8 (-2.33 to -1.27) |
| Belgium | 146 (134-158) | 3.926 (3.609-4.242) | 29 (26-32) | 0.833 (0.752-0.922) | -4.94 (-5.11 to -4.77) |
| Belize | 3 (2-3) | 3.713 (3.33-4.106) | 4 (4-5) | 2.157 (1.881-2.449) | -2.13 (-2.55 to -1.7) |
| Benin | 29 (22-38) | 1.68 (1.271-2.233) | 99 (71-131) | 1.882 (1.36-2.498) | 0.4 (0.1 to 0.71) |
| Bermuda | 1 (1-2) | 5.649 (5.114-6.282) | 0 (0-0) | 1.588 (1.247-2.004) | -4.52 (-4.79 to -4.24) |
| Bhutan | 17 (10-24) | 6.261 (3.83-8.737) | 21 (14-31) | 6.198 (3.945-9.027) | -0.41 (-0.56 to -0.26) |
| Bolivia (Plurinational State of) | 191 (144-259) | 7.74 (5.822-10.504) | 176 (121-244) | 3.586 (2.47-4.958) | -2.83 (-3.28 to -2.38) |
| Bosnia and Herzegovina | 127 (105-148) | 6.667 (5.534-7.78) | 31 (22-41) | 3.108 (2.157-4.095) | -3.08 (-3.51 to -2.64) |
| Botswana | 12 (6-18) | 2.303 (1.196-3.587) | 24 (13-36) | 2.282 (1.217-3.401) | -0.2 (-0.52 to 0.12) |
| Brazil | 3895 (3749-4037) | 6.207 (5.975-6.434) | 3807 (3648-3976) | 4.465 (4.279-4.664) | -1 (-1.3 to -0.7) |
| Brunei Darussalam | 14 (12-17) | 11.384 (9.525-13.391) | 15 (13-18) | 7.444 (6.289-8.72) | -2.04 (-2.61 to -1.47) |
| Bulgaria | 394 (362-425) | 13.226 (12.159-14.267) | 200 (169-236) | 10.546 (8.882-12.412) | -1.23 (-1.45 to -1) |
| Burkina Faso | 48 (34-65) | 1.497 (1.062-2.036) | 150 (102-214) | 1.734 (1.174-2.472) | 0.59 (0.4 to 0.77) |
| Burundi | 107 (78-149) | 5.185 (3.754-7.17) | 239 (174-318) | 4.528 (3.296-6.029) | -0.93 (-1.18 to -0.68) |
| Cabo Verde | 4 (3-5) | 3.248 (2.546-4.009) | 8 (5-11) | 3.045 (2.059-4.222) | -0.41 (-0.85 to 0.04) |
| Cambodia | 261 (210-319) | 6.791 (5.442-8.293) | 404 (284-589) | 5.58 (3.916-8.133) | -1.11 (-1.31 to -0.92) |
| Cameroon | 70 (50-96) | 1.846 (1.311-2.53) | 379 (253-553) | 2.94 (1.961-4.291) | 1.78 (1.16 to 2.41) |
| Canada | 336 (303-374) | 3.022 (2.722-3.366) | 177 (156-201) | 1.494 (1.311-1.695) | -2.71 (-2.98 to -2.44) |
| Central African Republic | 40 (27-59) | 3.857 (2.57-5.651) | 92 (54-142) | 4.212 (2.486-6.494) | 0.08 (-0.08 to 0.24) |
| Chad | 41 (29-56) | 1.936 (1.387-2.681) | 160 (109-217) | 2.536 (1.74-3.445) | 1.01 (0.76 to 1.26) |
| Chile | 130 (120-142) | 2.272 (2.089-2.48) | 132 (118-145) | 1.863 (1.671-2.043) | -0.48 (-0.6 to -0.35) |
| China | 29032 (25398-32762) | 5.297 (4.634-5.977) | 26397 (21936-31510) | 5.721 (4.754-6.829) | 0 (-0.16 to 0.16) |
| Colombia | 762 (712-816) | 5.422 (5.068-5.806) | 555 (468-644) | 2.762 (2.331-3.205) | -1.84 (-2.25 to -1.42) |
| Comoros | 6 (2-9) | 3.444 (1.445-5.023) | 12 (9-16) | 3.821 (2.783-5.043) | -0.36 (-0.94 to 0.23) |
| Congo | 44 (28-62) | 4.643 (2.991-6.512) | 100 (66-147) | 4.519 (2.989-6.636) | -0.33 (-0.58 to -0.08) |
| Cook Islands | 1 (1-1) | 9.201 (6.778-13.124) | 0 (0-1) | 7.325 (5.148-10.249) | -0.22 (-0.52 to 0.08) |
| Costa Rica | 48 (44-51) | 3.717 (3.425-4.009) | 67 (59-74) | 3.498 (3.097-3.911) | -0.47 (-1.11 to 0.17) |
| Côte d'Ivoire | 142 (98-191) | 2.997 (2.081-4.048) | 24 (21-28) | 1.959 (1.691-2.25) | -4.02 (-4.26 to -3.78) |
| Croatia | 128 (117-139) | 7.063 (6.459-7.633) | 108 (92-123) | 3.013 (2.574-3.437) | -2.79 (-3.03 to -2.55) |
| Cuba | 290 (270-310) | 5.936 (5.538-6.354) | 8 (7-10) | 1.654 (1.331-2.02) | -3.02 (-3.61 to -2.43) |
| Cyprus | 9 (8-11) | 3.072 (2.568-3.626) | 65 (57-75) | 2.217 (1.919-2.553) | -3.35 (-3.8 to -2.89) |
| Czechia | 278 (253-306) | 7.503 (6.821-8.235) | 1152 (808-1725) | 11.449 (8.029-17.155) | 1.02 (0.69 to 1.34) |
| Democratic People's Republic of Korea | 656 (453-925) | 7.87 (5.432-11.086) | 910 (620-1252) | 2.522 (1.717-3.469) | 0.77 (0.52 to 1.01) |
| Democratic Republic of the Congo | 397 (268-566) | 2.773 (1.869-3.953) | 16 (14-17) | 0.86 (0.79-0.944) | -0.42 (-0.58 to -0.27) |
| Denmark | 74 (68-80) | 3.88 (3.567-4.205) | 23 (14-33) | 4.248 (2.667-6.052) | -5 (-5.34 to -4.66) |
| Djibouti | 4 (3-6) | 2.561 (1.625-3.624) | 1 (0-1) | 2.005 (1.479-2.695) | 1.58 (1.22 to 1.94) |
| Dominica | 1 (1-1) | 2.143 (1.774-2.572) | 438 (344-552) | 9.638 (7.559-12.138) | -0.6 (-0.87 to -0.32) |
| Dominican Republic | 259 (224-298) | 8.439 (7.297-9.689) | 352 (285-429) | 4.824 (3.904-5.878) | 1.16 (0.79 to 1.53) |
| Ecuador | 251 (236-268) | 6.093 (5.716-6.485) | 9162 (7611-10784) | 21.704 (18.03-25.547) | -1.16 (-1.61 to -0.7) |
| Egypt | 6265 (5513-7061) | 28.576 (25.148-32.21) | 141 (110-177) | 5.426 (4.228-6.812) | -0.51 (-0.82 to -0.19) |
| El Salvador | 169 (152-190) | 8.095 (7.273-9.089) | 23 (13-36) | 3.291 (1.915-5.161) | -1.09 (-1.56 to -0.62) |
| Equatorial Guinea | 6 (4-8) | 3.771 (2.477-5.553) | 146 (95-208) | 5.221 (3.404-7.442) | -0.69 (-0.97 to -0.4) |
| Eritrea | 48 (34-66) | 3.707 (2.595-5.114) | 6 (5-7) | 1.534 (1.279-1.809) | 1.12 (0.96 to 1.28) |
| Estonia | 56 (49-65) | 9.921 (8.545-11.441) | 19 (12-29) | 3.746 (2.273-5.681) | -6.67 (-7.16 to -6.17) |
| Eswatini | 5 (3-7) | 1.646 (0.949-2.373) | 1293 (999-1600) | 2.79 (2.156-3.451) | 3.3 (2.49 to 4.11) |
| Ethiopia | 893 (706-1254) | 4.887 (3.867-6.866) | 64 (50-80) | 18.024 (13.889-22.43) | -2.19 (-2.39 to -1.98) |
| Fiji | 63 (53-77) | 19.692 (16.423-23.812) | 25 (23-28) | 1.52 (1.352-1.702) | -0.3 (-0.58 to -0.02) |
| Finland | 115 (104-125) | 6.317 (5.742-6.883) | 174 (154-195) | 0.875 (0.777-0.983) | -4.4 (-4.63 to -4.16) |
| France | 511 (463-564) | 2.323 (2.106-2.562) | 21 (13-31) | 2.76 (1.716-4.138) | -3.14 (-3.3 to -2.98) |
| Gabon | 11 (8-16) | 2.969 (2.063-4.077) | 38 (28-54) | 3.826 (2.77-5.361) | -0.41 (-0.59 to -0.22) |
| Gambia | 10 (7-14) | 2.649 (1.85-3.656) | 77 (70-84) | 6.795 (6.126-7.435) | 0.98 (0.68 to 1.29) |
| Georgia | 327 (308-347) | 15.341 (14.459-16.279) | 296 (268-331) | 1.17 (1.058-1.307) | -4.91 (-5.89 to -3.92) |
| Germany | 1526 (1406-1648) | 5.136 (4.733-5.548) | 419 (302-588) | 2.928 (2.112-4.112) | -4.83 (-5.09 to -4.58) |
| Ghana | 270 (206-345) | 4.705 (3.591-6.006) | 100 (93-108) | 3.587 (3.334-3.873) | -2.04 (-2.43 to -1.66) |
| Greece | 203 (191-216) | 5.408 (5.093-5.738) | 0 (0-0) | 1.825 (1.23-2.388) | -0.86 (-1.44 to -0.28) |
| Greenland | 1 (1-2) | 5.549 (4.269-7.191) | 1 (1-1) | 2.655 (2.212-3.183) | -4.71 (-5.17 to -4.25) |
| Grenada | 2 (2-3) | 6.993 (6.264-7.856) | 8 (7-9) | 13.861 (12.278-15.662) | -3.5 (-4.05 to -2.94) |
| Guam | 5 (4-6) | 8.178 (6.309-9.598) | 365 (317-418) | 5.367 (4.656-6.147) | 1.96 (1.64 to 2.27) |
| Guatemala | 304 (287-322) | 10.275 (9.7-10.902) | 162 (114-222) | 3.143 (2.215-4.293) | -2.35 (-3.1 to -1.6) |
| Guinea | 47 (35-62) | 2.271 (1.684-3.024) | 46 (32-64) | 5.452 (3.843-7.57) | 1.44 (1.3 to 1.57) |
| Guinea-Bissau | 17 (13-24) | 4.706 (3.405-6.574) | 15 (11-19) | 4.787 (3.644-6.115) | 0.59 (0.54 to 0.64) |
| Guyana | 28 (24-32) | 8.337 (6.995-9.49) | 436 (303-598) | 7.948 (5.517-10.89) | -2.09 (-2.46 to -1.72) |
| Haiti | 238 (180-308) | 9.775 (7.397-12.663) | 113 (59-174) | 2.574 (1.338-3.96) | -0.32 (-0.62 to -0.03) |
| Honduras | 77 (63-93) | 4.469 (3.624-5.411) | 83 (74-93) | 3.026 (2.676-3.382) | -2.38 (-2.74 to -2.01) |
| Hungary | 540 (504-579) | 14.624 (13.624-15.665) | 2 (2-2) | 1.735 (1.561-1.934) | -5.43 (-5.95 to -4.91) |
| Iceland | 5 (4-5) | 4.54 (4.175-4.934) | 62295 (56687-68167) | 10.221 (9.301-11.185) | -2.67 (-3.14 to -2.2) |
| India | 39860 (35117-44699) | 11.688 (10.297-13.107) | 12508 (10151-16245) | 10.984 (8.914-14.266) | -0.39 (-0.66 to -0.12) |
| Indonesia | 7678 (6568-8901) | 9.838 (8.416-11.405) | 2526 (2388-2687) | 7.28 (6.881-7.742) | 0.58 (0.41 to 0.75) |
| Iran (Islamic Republic of) | 2053 (1841-2241) | 9.455 (8.474-10.32) | 860 (658-1224) | 4.934 (3.773-7.018) | -0.85 (-0.96 to -0.74) |
| Iraq | 558 (446-699) | 7.769 (6.214-9.729) | 19 (17-22) | 1.208 (1.063-1.386) | -1.32 (-1.68 to -0.95) |
| Ireland | 55 (51-59) | 3.981 (3.691-4.3) | 20 (19-22) | 0.615 (0.561-0.676) | -3.64 (-3.91 to -3.38) |
| Israel | 68 (63-74) | 3.564 (3.309-3.857) | 186 (178-195) | 1.177 (1.126-1.232) | -5.73 (-6.01 to -5.46) |
| Italy | 633 (616-650) | 2.963 (2.885-3.045) | 16 (12-22) | 1.382 (1.02-1.873) | -2.75 (-3.02 to -2.47) |
| Jamaica | 17 (15-19) | 1.716 (1.496-1.964) | 409 (398-421) | 1.261 (1.227-1.298) | -1.8 (-2.57 to -1.03) |
| Japan | 1000 (975-1023) | 2.232 (2.176-2.283) | 224 (179-273) | 4.176 (3.337-5.094) | -1.47 (-2.01 to -0.93) |
| Jordan | 124 (105-146) | 8.064 (6.836-9.469) | 262 (199-332) | 3.757 (2.858-4.767) | -2.64 (-2.88 to -2.41) |
| Kazakhstan | 867 (741-994) | 12.774 (10.91-14.638) | 490 (367-648) | 2.262 (1.693-2.992) | -5.55 (-6.64 to -4.45) |
| Kenya | 115 (88-142) | 1.311 (1.006-1.622) | 10 (7-13) | 20.674 (14.683-26.406) | 2.22 (1.69 to 2.75) |
| Kiribati | 4 (3-6) | 14.701 (11.008-18.863) | 205 (173-247) | 9.656 (8.168-11.622) | 0.74 (0.55 to 0.93) |
| Kuwait | 94 (87-101) | 11.106 (10.273-11.972) | 180 (149-213) | 6.612 (5.49-7.837) | -0.67 (-1.1 to -0.23) |
| Kyrgyzstan | 205 (177-234) | 11.358 (9.829-12.947) | 416 (293-585) | 12.982 (9.128-18.224) | -3.81 (-4.42 to -3.19) |
| Lao People's Democratic Republic | 277 (202-366) | 17.912 (13.078-23.72) | 21 (18-26) | 3.974 (3.265-4.888) | -1.33 (-1.46 to -1.2) |
| Latvia | 107 (97-118) | 11.261 (10.162-12.41) | 103 (86-123) | 4.458 (3.694-5.311) | -4.88 (-5.51 to -4.24) |
| Lebanon | 123 (94-158) | 10.658 (8.165-13.732) | 21 (13-31) | 2.504 (1.547-3.743) | -2.39 (-3.04 to -1.74) |
| Lesotho | 4 (2-6) | 0.749 (0.45-1.139) | 78 (56-108) | 3.487 (2.508-4.819) | 5.44 (4.75 to 6.13) |
| Liberia | 24 (18-32) | 2.616 (1.946-3.5) | 440 (335-570) | 14.664 (11.15-18.988) | 1.34 (1.04 to 1.63) |
| Libya | 191 (147-237) | 11.368 (8.735-14.133) | 30 (26-33) | 3.666 (3.202-4.143) | 1.47 (1.17 to 1.77) |
| Lithuania | 157 (141-173) | 11.283 (10.115-12.42) | 1 (1-2) | 0.653 (0.57-0.758) | -3.55 (-4.17 to -2.92) |
| Luxembourg | 6 (6-7) | 4.154 (3.801-4.565) | 697 (471-954) | 5.948 (4.019-8.144) | -6.86 (-7.26 to -6.46) |
| Madagascar | 231 (189-282) | 5.095 (4.176-6.234) | 363 (271-468) | 4.435 (3.306-5.713) | 0.24 (0.14 to 0.34) |
| Malawi | 131 (102-163) | 3.514 (2.719-4.35) | 1017 (880-1162) | 7.313 (6.329-8.361) | 0.66 (0.33 to 0.99) |
| Malaysia | 507 (436-577) | 6.826 (5.872-7.773) | 17 (13-21) | 6.394 (4.884-8.083) | 0.12 (-0.2 to 0.45) |
| Maldives | 9 (7-12) | 11.051 (8.704-14.333) | 203 (151-282) | 2.284 (1.696-3.161) | -2.16 (-2.64 to -1.68) |
| Mali | 70 (49-97) | 2.329 (1.646-3.263) | 2 (2-3) | 1.695 (1.494-1.91) | 0.11 (-0.01 to 0.23) |
| Malta | 6 (6-7) | 4.444 (4.113-4.758) | 6 (4-8) | 25.442 (18.852-32.704) | -2.78 (-3.14 to -2.41) |
| Marshall Islands | 3 (2-3) | 15.392 (12.428-19.529) | 36 (22-54) | 2.12 (1.275-3.139) | 1.83 (1.7 to 1.96) |
| Mauritania | 24 (17-32) | 3.126 (2.216-4.222) | 49 (45-53) | 10.817 (9.844-11.724) | -1.41 (-1.52 to -1.3) |
| Mauritius | 68 (62-73) | 13.633 (12.554-14.601) | 3472 (3160-3814) | 6.74 (6.135-7.404) | 0.58 (0.23 to 0.92) |
| Mexico | 1332 (1300-1362) | 3.736 (3.646-3.818) | 9 (7-12) | 22.172 (16.074-29.198) | 2.19 (1.75 to 2.64) |
| Micronesia (Federated States of) | 8 (5-11) | 19.219 (13.394-26.337) | 0 (0-0) | 1.961 (1.182-2.954) | 0.67 (0.59 to 0.74) |
| Monaco | 0 (0-0) | 3.472 (2.674-4.431) | 84 (64-111) | 6.672 (5.053-8.78) | -2.17 (-2.49 to -1.85) |
| Mongolia | 60 (46-80) | 6.842 (5.159-9.044) | 8 (6-10) | 4.061 (3.129-5.065) | -0.83 (-1.3 to -0.35) |
| Montenegro | 19 (16-22) | 7.558 (6.341-8.847) | 1288 (908-2010) | 8.775 (6.186-13.688) | -2.24 (-2.6 to -1.89) |
| Morocco | 1648 (1296-2095) | 15.848 (12.465-20.151) | 216 (136-321) | 1.793 (1.131-2.67) | -2.23 (-2.45 to -2.02) |
| Mozambique | 42 (32-54) | 0.888 (0.67-1.14) | 1898 (1441-2547) | 8.442 (6.409-11.328) | 3.14 (2.8 to 3.48) |
| Myanmar | 2791 (1957-3944) | 16.255 (11.402-22.971) | 25 (14-41) | 2.423 (1.324-3.902) | -2.26 (-2.52 to -1.99) |
| Namibia | 10 (6-14) | 1.82 (1.102-2.489) | 2 (1-3) | 42.583 (31.009-58.313) | 0.56 (-0.01 to 1.14) |
| Nauru | 1 (1-2) | 35.877 (26.432-48.441) | 983 (687-1372) | 7.333 (5.124-10.235) | 0.55 (0.23 to 0.87) |
| Nepal | 649 (458-885) | 8.89 (6.264-12.112) | 35 (32-38) | 0.662 (0.598-0.726) | -0.48 (-0.62 to -0.33) |
| Netherlands | 205 (190-219) | 3.403 (3.154-3.637) | 25 (22-27) | 1.376 (1.243-1.513) | -6 (-6.57 to -5.42) |
| New Zealand | 54 (50-58) | 3.892 (3.628-4.165) | 105 (86-130) | 3.686 (3.028-4.551) | -4.19 (-4.5 to -3.88) |
| Nicaragua | 56 (49-64) | 3.789 (3.315-4.362) | 113 (68-165) | 1.268 (0.767-1.856) | 0.21 (-0.11 to 0.53) |
| Niger | 36 (22-58) | 1.309 (0.789-2.082) | 2266 (1477-3063) | 2.519 (1.642-3.406) | -0.06 (-0.18 to 0.06) |
| Nigeria | 801 (581-1083) | 2.346 (1.703-3.172) | 0 (0-0) | 16.818 (12.833-24.439) | 0.38 (0.2 to 0.57) |
| Niue | 0 (0-0) | 15.078 (10.376-22.448) | 25 (19-32) | 3.288 (2.485-4.232) | -0.12 (-0.35 to 0.11) |
| North Macedonia | 58 (49-69) | 7.335 (6.145-8.734) | 2 (1-2) | 9.22 (7.137-12.342) | -2.84 (-3.14 to -2.53) |
| Northern Mariana Islands | 2 (1-3) | 8.781 (5.785-12.228) | 9 (9-10) | 0.521 (0.493-0.549) | 0.15 (-0.22 to 0.52) |
| Norway | 57 (55-59) | 3.543 (3.43-3.671) | 132 (107-167) | 5.703 (4.609-7.235) | -5.94 (-6.37 to -5.51) |
| Oman | 74 (55-98) | 8.946 (6.681-11.854) | 14238 (11256-18160) | 14.395 (11.38-18.361) | -0.86 (-1.11 to -0.6) |
| Pakistan | 3568 (2572-4415) | 8.744 (6.303-10.82) | 2 (1-2) | 28.77 (22.546-36.265) | 1.51 (1.29 to 1.72) |
| Palau | 1 (1-2) | 18.107 (13.357-24.307) | 115 (96-135) | 5.252 (4.416-6.191) | 1.68 (1.52 to 1.84) |
| Palestine | 64 (49-83) | 8.297 (6.411-10.866) | 46 (38-55) | 2.786 (2.279-3.347) | -1.62 (-1.93 to -1.32) |
| Panama | 28 (26-30) | 2.733 (2.531-2.968) | 451 (290-634) | 10.528 (6.786-14.819) | 0.07 (-0.29 to 0.44) |
| Papua New Guinea | 140 (84-217) | 8.466 (5.088-13.081) | 85 (65-105) | 2.763 (2.114-3.438) | 0.63 (0.45 to 0.81) |
| Paraguay | 59 (48-70) | 3.772 (3.075-4.448) | 529 (407-681) | 3.556 (2.738-4.585) | -0.98 (-1.14 to -0.81) |
| Peru | 478 (400-569) | 5.383 (4.514-6.412) | 6912 (5895-7966) | 14.628 (12.477-16.86) | -1.16 (-1.63 to -0.7) |
| Philippines | 4014 (3698-4369) | 15.488 (14.267-16.855) | 220 (203-239) | 1.822 (1.679-1.977) | 0.12 (-0.08 to 0.32) |
| Poland | 2078 (2041-2118) | 14.384 (14.128-14.662) | 45 (40-50) | 1.509 (1.363-1.7) | -6.58 (-7.34 to -5.82) |
| Portugal | 144 (130-158) | 3.792 (3.446-4.178) | 28 (23-32) | 2.694 (2.258-3.136) | -3.75 (-4.39 to -3.11) |
| Puerto Rico | 69 (63-74) | 4.844 (4.448-5.247) | 58 (44-74) | 3.51 (2.659-4.469) | -2.69 (-3.26 to -2.11) |
| Qatar | 22 (18-27) | 9.204 (7.463-11.249) | 424 (291-605) | 3.784 (2.594-5.394) | -3.95 (-4.43 to -3.48) |
| Republic of Korea | 613 (488-749) | 2.912 (2.317-3.559) | 176 (144-217) | 1.101 (0.902-1.358) | -4.25 (-4.59 to -3.9) |
| Republic of Moldova | 161 (149-172) | 9.217 (8.529-9.876) | 108 (97-120) | 8.725 (7.809-9.676) | -0.32 (-0.72 to 0.07) |
| Romania | 777 (709-854) | 8.94 (8.16-9.828) | 137 (92-198) | 3.795 (2.562-5.503) | -2.39 (-2.7 to -2.08) |
| Russian Federation | 7684 (7434-7876) | 13.21 (12.779-13.539) | 276 (243-313) | 5.124 (4.516-5.816) | -2.54 (-3.18 to -1.89) |
| Rwanda | 136 (97-190) | 4.953 (3.555-6.943) | 4008 (3747-4294) | 8.623 (8.062-9.239) | -3.4 (-3.91 to -2.88) |
| Saint Kitts and Nevis | 1 (1-1) | 7.407 (6.75-8.188) | 144 (97-218) | 2.542 (1.704-3.846) | -6.44 (-7.25 to -5.62) |
| Saint Lucia | 2 (2-2) | 3.278 (2.975-3.613) | 0 (0-0) | 1.396 (0.91-2.046) | -3.25 (-3.57 to -2.94) |
| Saint Vincent and the Grenadines | 3 (3-3) | 6.375 (5.785-6.95) | 1 (1-1) | 1.427 (1.187-1.719) | -2.96 (-3.22 to -2.7) |
| Samoa | 6 (4-8) | 8.26 (5.642-11.612) | 1 (1-2) | 3.152 (2.692-3.747) | 1.77 (1.63 to 1.91) |
| San Marino | 0 (0-0) | 1.36 (1.101-1.684) | 11 (8-15) | 13.918 (9.662-18.852) | -2.81 (-3.1 to -2.52) |
| Sao Tome and Principe | 1 (1-1) | 2.114 (1.399-2.882) | 0 (0-0) | 0.483 (0.272-0.733) | 0.96 (0.46 to 1.47) |
| Saudi Arabia | 626 (465-827) | 9.424 (6.992-12.439) | 3 (2-4) | 2.891 (1.761-4.398) | 2.2 (1.84 to 2.55) |
| Senegal | 93 (69-119) | 3.372 (2.485-4.316) | 2793 (1937-3904) | 15.078 (10.456-21.076) | -0.36 (-0.56 to -0.16) |
| Serbia | 281 (244-316) | 7.817 (6.802-8.807) | 185 (136-253) | 2.873 (2.102-3.921) | -3.51 (-3.63 to -3.39) |
| Seychelles | 3 (3-4) | 10.059 (8.792-11.518) | 81 (64-98) | 2.74 (2.162-3.304) | -1.47 (-1.81 to -1.13) |
| Sierra Leone | 50 (34-68) | 3.118 (2.128-4.254) | 2 (2-3) | 6.431 (5.504-7.479) | 1.18 (0.99 to 1.38) |
| Singapore | 94 (89-100) | 6.233 (5.869-6.622) | 147 (102-206) | 3.942 (2.732-5.525) | -3.16 (-3.44 to -2.89) |
| Slovakia | 203 (177-231) | 9.931 (8.624-11.305) | 54 (50-57) | 2.784 (2.596-2.973) | -3.07 (-3.33 to -2.8) |
| Slovenia | 28 (25-31) | 3.665 (3.318-4.054) | 58 (47-71) | 3.409 (2.772-4.155) | -5.44 (-5.78 to -5.1) |
| Solomon Islands | 18 (10-25) | 13.944 (7.592-19.526) | 4 (3-5) | 0.714 (0.596-0.855) | 0.98 (0.88 to 1.08) |
| Somalia | 83 (56-124) | 2.874 (1.94-4.28) | 49 (36-65) | 18.02 (13.021-23.776) | 0.72 (0.56 to 0.88) |
| South Africa | 943 (843-1065) | 5.99 (5.357-6.766) | 265 (174-389) | 3.198 (2.102-4.688) | -2.23 (-3.2 to -1.26) |
| South Sudan | 58 (40-83) | 2.534 (1.749-3.615) | 874 (774-990) | 3.605 (3.194-4.081) | 1.21 (0.73 to 1.69) |
| Spain | 556 (516-599) | 3.746 (3.478-4.037) | 188 (169-208) | 1.515 (1.365-1.674) | -3.17 (-3.35 to -3) |
| Sri Lanka | 562 (487-658) | 7.597 (6.59-8.893) | 467 (330-631) | 5.789 (4.092-7.82) | -1.19 (-1.54 to -0.84) |
| Sudan | 1472 (1061-1985) | 19.317 (13.922-26.044) | 2439 (1431-3570) | 13.193 (7.739-19.313) | -1.19 (-1.31 to -1.07) |
| Suriname | 12 (9-15) | 7.542 (5.585-8.91) | 11 (8-13) | 4.893 (3.773-6.234) | -1.75 (-2.14 to -1.35) |
| Sweden | 68 (64-74) | 2.333 (2.177-2.512) | 14 (12-16) | 0.436 (0.38-0.498) | -5.15 (-5.42 to -4.89) |
| Switzerland | 99 (91-108) | 3.761 (3.452-4.1) | 18 (16-20) | 0.645 (0.574-0.724) | -6.35 (-6.66 to -6.04) |
| Syrian Arab Republic | 1357 (1100-1642) | 28.287 (22.929-34.218) | 786 (591-1044) | 15.457 (11.623-20.53) | -1.75 (-2.06 to -1.43) |
| Taiwan (Province of China) | 245 (226-265) | 2.657 (2.445-2.87) | 152 (136-168) | 2.011 (1.803-2.23) | -0.22 (-0.51 to 0.07) |
| Tajikistan | 211 (174-249) | 9.98 (8.212-11.786) | 266 (201-335) | 6.371 (4.83-8.019) | -2.99 (-3.61 to -2.36) |
| Thailand | 1100 (804-1368) | 4.241 (3.1-5.276) | 1193 (915-1546) | 5.625 (4.314-7.292) | -0.74 (-1.63 to 0.16) |
| Timor-Leste | 23 (17-31) | 7.283 (5.245-9.739) | 45 (30-64) | 7.801 (5.265-11.136) | 0.24 (-0.32 to 0.8) |
| Togo | 39 (30-50) | 2.852 (2.2-3.653) | 110 (74-155) | 3.257 (2.196-4.597) | 0.32 (0.06 to 0.59) |
| Tokelau | 0 (0-0) | 11.717 (7.773-17.037) | 0 (0-0) | 16.562 (13.58-21.284) | 0.79 (0.61 to 0.98) |
| Tonga | 2 (2-3) | 5.659 (4.402-7.075) | 3 (2-4) | 8.03 (5.858-11.541) | 1.33 (1.11 to 1.55) |
| Trinidad and Tobago | 40 (37-43) | 7.977 (7.379-8.578) | 35 (27-45) | 7.037 (5.453-8.976) | -0.9 (-1.51 to -0.29) |
| Tunisia | 258 (197-323) | 7.513 (5.729-9.4) | 296 (205-410) | 6.808 (4.717-9.42) | -0.66 (-0.78 to -0.54) |
| Turkey | 2074 (1704-2508) | 8.67 (7.126-10.487) | 1234 (968-1518) | 3.872 (3.038-4.766) | -2.23 (-3.08 to -1.38) |
| Turkmenistan | 190 (168-212) | 12.396 (10.927-13.787) | 215 (164-285) | 10.346 (7.885-13.687) | 0.53 (0.45 to 0.61) |
| Tuvalu | 1 (0-1) | 18.617 (13.695-23.966) | 1 (1-1) | 22.252 (16.79-28.118) | -2.94 (-3.3 to -2.59) |
| Uganda | 143 (97-197) | 2.225 (1.511-3.071) | 549 (384-757) | 3.19 (2.231-4.403) | 0.15 (-0.45 to 0.75) |
| Ukraine | 1592 (1364-1867) | 8.383 (7.18-9.83) | 1742 (1269-2318) | 12.639 (9.204-16.815) | -0.04 (-0.53 to 0.46) |
| United Arab Emirates | 72 (53-96) | 7.503 (5.555-10.083) | 166 (116-218) | 4.143 (2.878-5.424) | -2.04 (-2.34 to -1.73) |
| United Kingdom | 803 (793-813) | 3.842 (3.796-3.888) | 316 (309-325) | 1.453 (1.419-1.493) | -3.09 (-3.26 to -2.91) |
| United Republic of Tanzania | 312 (233-405) | 3.221 (2.405-4.18) | 1157 (800-1559) | 4.958 (3.428-6.681) | 1.36 (1.16 to 1.56) |
| United States of America | 4238 (4159-4332) | 4.148 (4.071-4.24) | 3100 (2909-3255) | 2.786 (2.613-2.924) | -1.5 (-1.67 to -1.33) |
| United States Virgin Islands | 3 (3-4) | 8.722 (6.856-10.906) | 2 (1-2) | 6.91 (4.416-9.495) | -0.97 (-1.33 to -0.61) |
| Uruguay | 53 (49-57) | 4.67 (4.303-5.032) | 24 (22-26) | 1.979 (1.801-2.195) | -2.58 (-3.04 to -2.11) |
| Uzbekistan | 972 (863-1077) | 11.32 (10.06-12.55) | 1606 (1340-1873) | 11.691 (9.754-13.634) | -0.17 (-0.48 to 0.14) |
| Vanuatu | 12 (8-16) | 20.469 (14.11-26.927) | 31 (23-39) | 25.044 (18.388-31.524) | 0.48 (0.4 to 0.56) |
| Venezuela (Bolivarian Republic of) | 624 (585-670) | 7.792 (7.304-8.372) | 769 (590-995) | 8.216 (6.307-10.631) | 0.01 (-0.48 to 0.51) |
| Viet Nam | 789 (559-1028) | 2.768 (1.96-3.604) | 1201 (856-1676) | 3.129 (2.229-4.366) | 0.72 (0.36 to 1.09) |
| Yemen | 519 (305-805) | 11.299 (6.629-17.507) | 1233 (783-1871) | 8.963 (5.693-13.6) | -0.71 (-0.84 to -0.58) |
| Zambia | 73 (55-94) | 2.391 (1.825-3.082) | 280 (184-396) | 3.462 (2.278-4.89) | 1.19 (0.97 to 1.42) |
| Zimbabwe | 40 (31-51) | 1.021 (0.793-1.289) | 208 (138-296) | 3.286 (2.176-4.663) | 5.09 (3.99 to 6.21) |
| **Disability-adjusted life yearsss** |  |  |  |  |  |
| Afghanistan | 28175 (19111-38983) | 894.587 (606.794-1237.735) | 93786 (67783-129751) | 767.415 (554.643-1061.706) | -0.14 (-0.61 to 0.32) |
| Albania | 3674 (3143-4284) | 258.645 (221.253-301.6) | 1871 (1524-2302) | 197.307 (160.734-242.853) | -1.35 (-1.75 to -0.96) |
| Algeria | 74097 (58649-93081) | 733.31 (580.427-921.196) | 75240 (57706-95942) | 441.886 (338.91-563.471) | -2.2 (-2.51 to -1.88) |
| American Samoa | 116 (92-147) | 575.747 (456.72-726.58) | 133 (98-170) | 760.203 (562.721-972.653) | 1.04 (0.97 to 1.11) |
| Andorra | 32 (24-44) | 129.598 (95.99-175.443) | 16 (11-21) | 61.36 (41.815-82.401) | -2.21 (-2.5 to -1.92) |
| Angola | 6742 (4744-8926) | 172.325 (121.255-228.136) | 21253 (14602-29258) | 174.693 (120.019-240.489) | 0.27 (0.09 to 0.45) |
| Antigua and Barbuda | 64 (58-71) | 248.087 (224.353-276.439) | 22 (18-26) | 62.996 (51.768-74.966) | -3.6 (-4.08 to -3.12) |
| Argentina | 42943 (39692-46304) | 351.558 (324.937-379.068) | 22191 (20281-24131) | 126.645 (115.741-137.714) | -3.05 (-3.58 to -2.52) |
| Armenia | 7958 (6913-8894) | 553.696 (481.03-618.866) | 3813 (3308-4320) | 354.666 (307.714-401.861) | -3.01 (-3.54 to -2.47) |
| Australia | 12201 (11194-13246) | 180.162 (165.287-195.593) | 6473 (5786-7241) | 74.663 (66.742-83.526) | -3.06 (-3.43 to -2.7) |
| Austria | 5797 (5367-6250) | 193.114 (178.767-208.184) | 1735 (1580-1899) | 61.477 (55.992-67.281) | -4.57 (-4.94 to -4.2) |
| Azerbaijan | 26142 (22413-30323) | 822.572 (705.235-954.126) | 20003 (15945-24702) | 472.235 (376.431-583.149) | -3.41 (-3.9 to -2.93) |
| Bahamas | 426 (381-472) | 360.638 (322.467-399.581) | 307 (239-391) | 198.372 (154.652-252.766) | -2.6 (-2.85 to -2.35) |
| Bahrain | 1891 (1674-2126) | 737.622 (652.886-829.495) | 2749 (2305-3228) | 390.67 (327.664-458.818) | -2.66 (-3.09 to -2.23) |
| Bangladesh | 190292 (152814-237459) | 450.821 (362.032-562.565) | 259029 (188883-337097) | 376.419 (274.483-489.866) | -0.51 (-0.85 to -0.16) |
| Barbados | 237 (214-262) | 217.114 (196.19-239.799) | 92 (71-117) | 92.786 (71.425-118.864) | -3.19 (-3.53 to -2.84) |
| Belarus | 25456 (22692-28193) | 645.356 (575.29-714.741) | 17402 (14087-20985) | 593.19 (480.195-715.308) | -1.82 (-2.35 to -1.29) |
| Belgium | 8460 (7821-9110) | 227.373 (210.197-244.844) | 1762 (1599-1945) | 50.306 (45.649-55.529) | -4.78 (-4.95 to -4.62) |
| Belize | 164 (147-182) | 224.401 (201.281-248.167) | 241 (212-273) | 127.814 (112.188-144.632) | -2.19 (-2.59 to -1.78) |
| Benin | 1707 (1306-2247) | 100.233 (76.685-131.962) | 5943 (4320-7857) | 113.362 (82.402-149.866) | 0.44 (0.14 to 0.73) |
| Bermuda | 85 (77-94) | 330.299 (300.898-364.7) | 17 (13-21) | 95.507 (76.719-119.81) | -4.39 (-4.66 to -4.12) |
| Bhutan | 1009 (618-1417) | 374.498 (229.327-525.975) | 1266 (809-1837) | 365.373 (233.503-530.012) | -0.44 (-0.59 to -0.28) |
| Bolivia (Plurinational State of) | 11635 (8834-15564) | 471.824 (358.228-631.118) | 10760 (7459-14832) | 218.969 (151.799-301.841) | -2.78 (-3.2 to -2.36) |
| Bosnia and Herzegovina | 7271 (6042-8449) | 382.841 (318.158-444.909) | 1832 (1278-2382) | 182.026 (126.959-236.703) | -2.99 (-3.4 to -2.57) |
| Botswana | 695 (363-1070) | 135.076 (70.456-207.769) | 1415 (773-2087) | 132.842 (72.57-195.847) | -0.2 (-0.51 to 0.12) |
| Brazil | 224578 (216175-232303) | 357.917 (344.525-370.228) | 222416 (213605-232583) | 260.898 (250.563-272.825) | -0.92 (-1.22 to -0.62) |
| Brunei Darussalam | 838 (700-984) | 679.799 (567.98-798.197) | 862 (731-1008) | 422.416 (358.505-494.012) | -2.17 (-2.73 to -1.61) |
| Bulgaria | 22388 (20611-24135) | 752.074 (692.35-810.727) | 11383 (9632-13405) | 599.243 (507.072-705.662) | -1.23 (-1.45 to -1.01) |
| Burkina Faso | 2847 (2045-3847) | 89.289 (64.143-120.678) | 9016 (6247-12660) | 104.18 (72.177-146.279) | 0.62 (0.44 to 0.79) |
| Burundi | 6522 (4782-8909) | 314.605 (230.681-429.744) | 14493 (10535-19001) | 274.888 (199.816-360.388) | -0.9 (-1.11 to -0.68) |
| Cabo Verde | 252 (199-311) | 192.952 (152.498-237.777) | 453 (315-624) | 180.707 (125.522-248.81) | -0.4 (-0.84 to 0.03) |
| Cambodia | 15515 (12489-18968) | 402.948 (324.362-492.642) | 23727 (16745-34386) | 327.58 (231.179-474.741) | -1.12 (-1.3 to -0.94) |
| Cameroon | 4192 (2985-5740) | 110.23 (78.485-150.936) | 22601 (15207-32620) | 175.297 (117.952-253.012) | 1.78 (1.16 to 2.41) |
| Canada | 19659 (17788-21694) | 176.845 (160.011-195.151) | 10491 (9294-11801) | 88.445 (78.357-99.491) | -2.63 (-2.88 to -2.37) |
| Central African Republic | 2372 (1609-3445) | 227.737 (154.534-330.851) | 5403 (3261-8216) | 247.477 (149.346-376.303) | 0.07 (-0.08 to 0.23) |
| Chad | 2428 (1776-3312) | 115.648 (84.615-157.807) | 9672 (6723-13084) | 153.701 (106.842-207.92) | 1.05 (0.8 to 1.3) |
| Chile | 7804 (7217-8476) | 136.255 (126.017-147.995) | 7846 (7132-8608) | 110.884 (100.793-121.655) | -0.5 (-0.62 to -0.38) |
| China | 1722218 (1508657-1940453) | 314.194 (275.233-354.008) | 1530640 (1279874-1822726) | 331.706 (277.362-395.004) | -0.04 (-0.19 to 0.11) |
| Colombia | 45505 (42682-48576) | 323.78 (303.698-345.631) | 33451 (28572-38589) | 166.473 (142.192-192.04) | -1.79 (-2.2 to -1.38) |
| Comoros | 364 (152-528) | 210.567 (87.788-305.372) | 715 (521-937) | 230.879 (168.294-302.403) | -0.43 (-1.03 to 0.19) |
| Congo | 2576 (1674-3601) | 271.952 (176.685-380.095) | 5838 (3917-8452) | 263.507 (176.81-381.457) | -0.36 (-0.61 to -0.11) |
| Cook Islands | 41 (30-58) | 525.138 (389.548-747.235) | 25 (17-34) | 417.184 (296.202-580.031) | -0.26 (-0.55 to 0.04) |
| Costa Rica | 2835 (2624-3056) | 220.664 (204.296-237.922) | 3953 (3525-4392) | 207.694 (185.18-230.756) | -0.45 (-1.06 to 0.17) |
| Côte d'Ivoire | 8463 (5959-11324) | 178.914 (125.975-239.4) | 1438 (1244-1645) | 115.169 (99.655-131.787) | -3.94 (-4.17 to -3.71) |
| Croatia | 7318 (6716-7896) | 403.3 (370.139-435.183) | 6326 (5437-7170) | 176.422 (151.633-199.956) | -2.79 (-3.03 to -2.55) |
| Cuba | 17005 (15888-18139) | 348.487 (325.589-371.733) | 483 (388-586) | 96.225 (77.261-116.776) | -3 (-3.57 to -2.42) |
| Cyprus | 545 (459-637) | 177.201 (149.399-206.955) | 3879 (3371-4426) | 131.358 (114.141-149.868) | -3.25 (-3.68 to -2.83) |
| Czechia | 15814 (14386-17345) | 426.184 (387.698-467.426) | 67114 (47510-100221) | 667.249 (472.344-996.392) | 0.99 (0.67 to 1.32) |
| Democratic People's Republic of Korea | 39256 (26945-55299) | 470.653 (323.048-662.995) | 53844 (37128-73652) | 149.211 (102.888-204.101) | 0.71 (0.49 to 0.94) |
| Democratic Republic of the Congo | 23634 (16162-33431) | 164.983 (112.824-233.372) | 952 (875-1039) | 52.209 (47.977-56.989) | -0.43 (-0.58 to -0.28) |
| Denmark | 4266 (3950-4603) | 223.586 (207.014-241.266) | 1358 (862-1926) | 251.178 (159.434-356.099) | -4.84 (-5.16 to -4.52) |
| Djibouti | 271 (175-381) | 154.597 (100.069-217.341) | 31 (23-41) | 118.428 (88.795-156.713) | 1.51 (1.15 to 1.88) |
| Dominica | 38 (32-45) | 128.508 (107.917-153.036) | 25818 (20364-32634) | 567.765 (447.836-717.654) | -0.62 (-0.9 to -0.34) |
| Dominican Republic | 15454 (13378-17669) | 503.063 (435.476-575.151) | 21378 (17392-25923) | 292.666 (238.101-354.889) | 1.14 (0.76 to 1.52) |
| Ecuador | 15294 (14373-16308) | 370.637 (348.32-395.22) | 549700 (457348-644723) | 1302.241 (1083.461-1527.353) | -1.16 (-1.61 to -0.7) |
| Egypt | 379432 (335480-430741) | 1730.792 (1530.305-1964.839) | 8388 (6637-10451) | 323.618 (256.052-403.222) | -0.52 (-0.82 to -0.21) |
| El Salvador | 10293 (9282-11556) | 492.628 (444.271-553.109) | 1364 (806-2138) | 196.092 (115.891-307.306) | -1.15 (-1.63 to -0.68) |
| Equatorial Guinea | 334 (222-486) | 221.431 (147.337-321.824) | 8694 (5775-12342) | 310.518 (206.239-440.796) | -0.65 (-0.91 to -0.38) |
| Eritrea | 2885 (2039-3955) | 222.619 (157.306-305.178) | 355 (299-415) | 89.711 (75.675-105.025) | 1.08 (0.92 to 1.25) |
| Estonia | 3164 (2738-3645) | 557.119 (482.076-641.768) | 1108 (679-1659) | 217.61 (133.312-325.799) | -6.52 (-7.01 to -6.04) |
| Eswatini | 295 (173-420) | 97.899 (57.439-139.233) | 79281 (61755-97841) | 171.032 (133.222-211.069) | 3.22 (2.41 to 4.03) |
| Ethiopia | 54381 (43148-76389) | 297.66 (236.17-418.12) | 3654 (2818-4547) | 1024.371 (789.851-1274.761) | -2.17 (-2.36 to -1.97) |
| Fiji | 3626 (3025-4362) | 1124.399 (938.19-1352.875) | 1514 (1351-1682) | 90.865 (81.116-100.955) | -0.3 (-0.57 to -0.03) |
| Finland | 6551 (5991-7115) | 360.829 (330.03-391.9) | 10443 (9427-11627) | 52.563 (47.452-58.527) | -4.24 (-4.47 to -4.01) |
| France | 29504 (26903-32232) | 134.106 (122.281-146.504) | 1215 (770-1807) | 162.109 (102.678-241.124) | -2.95 (-3.1 to -2.81) |
| Gabon | 667 (469-905) | 173.457 (121.895-235.203) | 2302 (1672-3205) | 230.123 (167.162-320.476) | -0.39 (-0.57 to -0.2) |
| Gambia | 599 (420-822) | 158.92 (111.432-217.895) | 4350 (3914-4756) | 383.381 (344.957-419.118) | 1 (0.7 to 1.3) |
| Georgia | 18593 (17527-19760) | 873.36 (823.265-928.172) | 17480 (15915-19455) | 69.096 (62.909-76.901) | -4.93 (-5.9 to -3.94) |
| Germany | 87529 (80802-94077) | 294.626 (271.983-316.669) | 24758 (18118-34675) | 173.111 (126.686-242.45) | -4.7 (-4.94 to -4.46) |
| Ghana | 15870 (12160-20120) | 276.476 (211.835-350.518) | 5662 (5254-6109) | 203.373 (188.732-219.438) | -2 (-2.38 to -1.62) |
| Greece | 11725 (11051-12410) | 311.896 (293.969-330.125) | 21 (15-28) | 104.889 (71.849-136.213) | -0.94 (-1.52 to -0.36) |
| Greenland | 84 (64-109) | 317.339 (243.024-410.938) | 63 (52-74) | 154.686 (129.787-184.247) | -4.64 (-5.08 to -4.2) |
| Grenada | 136 (122-152) | 408.126 (365.944-457.123) | 438 (389-496) | 790.64 (701.618-894.961) | -3.44 (-3.98 to -2.9) |
| Guam | 300 (233-350) | 472.586 (366.942-551.657) | 22140 (19244-25330) | 325.261 (282.719-372.13) | 1.92 (1.62 to 2.23) |
| Guatemala | 18440 (17393-19563) | 624.263 (588.821-662.269) | 9730 (6876-13300) | 188.316 (133.078-257.4) | -2.36 (-3.1 to -1.62) |
| Guinea | 2763 (2088-3649) | 134.513 (101.616-177.615) | 2723 (1930-3710) | 322.661 (228.69-439.648) | 1.48 (1.35 to 1.62) |
| Guinea-Bissau | 1038 (761-1431) | 279.931 (205.175-385.989) | 868 (664-1102) | 279.207 (213.572-354.704) | 0.58 (0.53 to 0.62) |
| Guyana | 1667 (1401-1896) | 489.683 (411.548-556.932) | 25365 (17845-34468) | 462.082 (325.074-627.905) | -2.11 (-2.46 to -1.76) |
| Haiti | 13883 (10584-17901) | 570.329 (434.773-735.39) | 6705 (3602-10171) | 152.541 (81.934-231.378) | -0.33 (-0.61 to -0.04) |
| Honduras | 4553 (3734-5500) | 263.743 (216.274-318.6) | 4779 (4271-5310) | 173.682 (155.233-193) | -2.34 (-2.7 to -1.99) |
| Hungary | 30146 (28167-32220) | 815.667 (762.104-871.775) | 123 (111-137) | 102.75 (92.528-114.088) | -5.33 (-5.84 to -4.81) |
| Iceland | 277 (256-300) | 266.639 (246.687-289.103) | 3616712 (3294029-3953291) | 593.434 (540.488-648.66) | -2.68 (-3.12 to -2.25) |
| India | 2344412 (2066682-2628300) | 687.457 (606.017-770.702) | 729245 (593704-943716) | 640.394 (521.367-828.733) | -0.45 (-0.72 to -0.18) |
| Indonesia | 453690 (387404-526653) | 581.327 (496.392-674.816) | 148554 (140708-157871) | 428.07 (405.46-454.917) | 0.53 (0.37 to 0.7) |
| Iran (Islamic Republic of) | 124349 (111486-135337) | 572.555 (513.328-623.149) | 51388 (39502-72153) | 294.714 (226.546-413.806) | -0.93 (-1.02 to -0.84) |
| Iraq | 33286 (26826-41425) | 463.257 (373.358-576.535) | 1108 (986-1257) | 70.781 (63.002-80.322) | -1.33 (-1.68 to -0.97) |
| Ireland | 3161 (2943-3408) | 230.457 (214.559-248.426) | 1285 (1168-1410) | 38.66 (35.144-42.421) | -3.63 (-3.89 to -3.36) |
| Israel | 3991 (3715-4303) | 208.843 (194.385-225.191) | 11214 (10659-11820) | 71 (67.483-74.832) | -5.52 (-5.79 to -5.26) |
| Italy | 37070 (35937-38093) | 173.644 (168.335-178.436) | 987 (739-1326) | 82.722 (61.927-111.134) | -2.68 (-2.93 to -2.43) |
| Jamaica | 1018 (890-1158) | 103.537 (90.545-117.791) | 24283 (23458-25223) | 74.924 (72.377-77.825) | -1.79 (-2.51 to -1.06) |
| Japan | 58917 (57309-60535) | 131.468 (127.881-135.079) | 13444 (10839-16225) | 250.444 (201.919-302.244) | -1.49 (-2.01 to -0.97) |
| Jordan | 7527 (6428-8852) | 489.739 (418.251-575.927) | 14908 (11378-18821) | 213.91 (163.26-270.054) | -2.68 (-2.91 to -2.45) |
| Kazakhstan | 49373 (42317-56431) | 727.252 (623.318-831.219) | 29949 (22684-39372) | 138.323 (104.769-181.843) | -5.53 (-6.62 to -4.43) |
| Kenya | 7192 (5560-8793) | 82.118 (63.493-100.408) | 584 (416-743) | 1174.3 (836.547-1493.427) | 2.14 (1.63 to 2.66) |
| Kiribati | 256 (193-328) | 839.383 (632.41-1075.897) | 11912 (10067-14196) | 561.155 (474.256-668.744) | 0.75 (0.57 to 0.93) |
| Kuwait | 5609 (5199-6041) | 663.65 (615.192-714.791) | 10232 (8520-12109) | 375.947 (313.065-444.921) | -0.73 (-1.16 to -0.3) |
| Kyrgyzstan | 11906 (10268-13609) | 660.104 (569.276-754.547) | 24628 (17366-34683) | 767.747 (541.353-1081.18) | -3.85 (-4.45 to -3.25) |
| Lao People's Democratic Republic | 16390 (11998-21755) | 1060.806 (776.536-1408.052) | 1209 (994-1483) | 224.382 (184.548-275.361) | -1.31 (-1.42 to -1.19) |
| Latvia | 6051 (5471-6637) | 634.334 (573.521-695.732) | 6218 (5183-7334) | 267.978 (223.389-316.071) | -4.83 (-5.44 to -4.21) |
| Lebanon | 7149 (5516-9123) | 620.187 (478.506-791.429) | 1213 (766-1797) | 145.879 (92.066-216.063) | -2.26 (-2.89 to -1.63) |
| Lesotho | 243 (149-363) | 44.945 (27.615-67.23) | 4655 (3370-6405) | 207.352 (150.095-285.262) | 5.32 (4.64 to 6) |
| Liberia | 1433 (1072-1900) | 155.274 (116.164-205.904) | 25880 (19750-33346) | 862.527 (658.217-1111.333) | 1.37 (1.09 to 1.65) |
| Libya | 11638 (8967-14348) | 692.67 (533.685-853.931) | 1658 (1450-1864) | 205.996 (180.104-231.524) | 1.35 (1.05 to 1.65) |
| Lithuania | 8858 (7978-9749) | 635.741 (572.581-699.696) | 89 (78-102) | 40.374 (35.388-46.456) | -3.55 (-4.17 to -2.93) |
| Luxembourg | 354 (325-388) | 240.037 (220.354-262.863) | 41812 (28322-56919) | 357.035 (241.846-486.038) | -6.64 (-7.02 to -6.27) |
| Madagascar | 13897 (11429-16875) | 306.799 (252.309-372.538) | 22103 (16760-28195) | 270.098 (204.816-344.542) | 0.23 (0.13 to 0.33) |
| Malawi | 8114 (6286-9989) | 217.155 (168.224-267.34) | 58696 (50967-66640) | 422.195 (366.605-479.34) | 0.6 (0.27 to 0.93) |
| Malaysia | 29931 (25840-33962) | 403.28 (348.157-457.594) | 965 (749-1214) | 371.057 (287.729-466.477) | 0.06 (-0.27 to 0.38) |
| Maldives | 533 (422-688) | 655.671 (519.51-846.095) | 12390 (9264-17144) | 139.1 (104.007-192.47) | -2.17 (-2.62 to -1.71) |
| Mali | 4141 (2971-5739) | 138.708 (99.506-192.231) | 133 (118-149) | 99.497 (88.367-111.364) | 0.18 (0.07 to 0.3) |
| Malta | 358 (332-383) | 259.266 (240.281-277.704) | 340 (252-436) | 1434.818 (1063.777-1837.949) | -2.77 (-3.11 to -2.43) |
| Marshall Islands | 153 (124-195) | 892.032 (720.077-1135.462) | 2181 (1346-3176) | 127.79 (78.832-186.081) | 1.78 (1.65 to 1.91) |
| Mauritania | 1428 (1017-1907) | 185.752 (132.334-248.065) | 2851 (2599-3080) | 626.383 (570.902-676.593) | -1.36 (-1.47 to -1.25) |
| Mauritius | 3875 (3578-4145) | 779.506 (719.819-833.816) | 205962 (188304-226145) | 399.836 (365.557-439.018) | 0.65 (0.3 to 1.01) |
| Mexico | 79901 (77899-81775) | 224.051 (218.437-229.305) | 535 (388-702) | 1259.801 (914.735-1653.477) | 2.18 (1.74 to 2.62) |
| Micronesia (Federated States of) | 435 (304-595) | 1086.769 (757.568-1484.674) | 11 (7-16) | 112.985 (69.898-168.183) | 0.68 (0.61 to 0.75) |
| Monaco | 18 (14-23) | 197.94 (155.063-250.705) | 4773 (3639-6251) | 378.143 (288.316-495.302) | -2.14 (-2.44 to -1.83) |
| Mongolia | 3528 (2687-4641) | 399.202 (304.081-525.196) | 482 (376-598) | 234.291 (182.749-290.861) | -0.9 (-1.36 to -0.44) |
| Montenegro | 1089 (919-1276) | 433.99 (366.296-508.248) | 75805 (53978-117184) | 516.335 (367.661-798.178) | -2.22 (-2.57 to -1.86) |
| Morocco | 97901 (77032-125346) | 941.652 (740.924-1205.633) | 13012 (8476-19004) | 108.223 (70.491-158.058) | -2.26 (-2.48 to -2.04) |
| Mozambique | 2599 (1995-3292) | 54.794 (42.052-69.397) | 112533 (85039-150633) | 500.554 (378.26-670.025) | 3.02 (2.7 to 3.35) |
| Myanmar | 166602 (117049-234246) | 970.455 (681.812-1364.481) | 1472 (820-2346) | 140.849 (78.461-224.485) | -2.28 (-2.54 to -2.02) |
| Namibia | 603 (370-815) | 107.823 (66.1-145.675) | 113 (83-155) | 2430.916 (1775.891-3321.01) | 0.52 (-0.04 to 1.08) |
| Nauru | 82 (61-111) | 2033.487 (1503.875-2755.947) | 58448 (41453-81539) | 435.931 (309.175-608.153) | 0.57 (0.25 to 0.9) |
| Nepal | 38443 (27254-52640) | 526.25 (373.079-720.589) | 2140 (1951-2348) | 40.532 (36.942-44.465) | -0.46 (-0.61 to -0.31) |
| Netherlands | 11759 (10948-12557) | 195.019 (181.563-208.257) | 1432 (1297-1570) | 79.477 (71.996-87.165) | -5.79 (-6.32 to -5.25) |
| New Zealand | 3076 (2868-3281) | 222.675 (207.637-237.472) | 6227 (5152-7576) | 218.785 (181.011-266.171) | -4.14 (-4.45 to -3.83) |
| Nicaragua | 3382 (2978-3875) | 229.129 (201.74-262.554) | 6963 (4316-10099) | 78.08 (48.401-113.249) | 0.14 (-0.16 to 0.45) |
| Niger | 2209 (1377-3502) | 79.353 (49.478-125.831) | 136211 (89270-183395) | 151.449 (99.256-203.911) | -0.01 (-0.13 to 0.11) |
| Nigeria | 47596 (34753-63674) | 139.398 (101.784-186.485) | 6 (4-8) | 970.33 (746.439-1393.9) | 0.42 (0.23 to 0.61) |
| Niue | 7 (5-10) | 857.823 (590.995-1271.941) | 1451 (1109-1859) | 189.67 (145.02-243.071) | -0.1 (-0.32 to 0.13) |
| North Macedonia | 3331 (2802-3969) | 419.509 (352.79-499.776) | 86 (67-114) | 519.059 (405.361-688.66) | -2.81 (-3.11 to -2.5) |
| Northern Mariana Islands | 115 (76-160) | 492.441 (325.464-682.169) | 569 (535-609) | 32.072 (30.151-34.292) | 0.13 (-0.21 to 0.47) |
| Norway | 3250 (3146-3361) | 203.163 (196.669-210.113) | 7783 (6320-9740) | 336.29 (273.089-420.852) | -5.76 (-6.15 to -5.37) |
| Oman | 4371 (3277-5760) | 526.833 (394.953-694.226) | 841121 (668755-1074634) | 850.422 (676.151-1086.518) | -0.81 (-1.07 to -0.54) |
| Pakistan | 211356 (151774-262192) | 517.967 (371.951-642.549) | 95 (75-119) | 1615.616 (1270.925-2024.622) | 1.5 (1.28 to 1.72) |
| Palau | 72 (53-96) | 1026.688 (761.114-1370.058) | 6983 (5872-8164) | 319.792 (268.927-373.877) | 1.66 (1.5 to 1.82) |
| Palestine | 3891 (3009-5032) | 506.945 (392.119-655.613) | 2753 (2278-3279) | 166.798 (138.049-198.671) | -1.6 (-1.91 to -1.29) |
| Panama | 1674 (1559-1814) | 165.511 (154.14-179.282) | 25868 (16678-36462) | 604.451 (389.706-852.019) | 0.06 (-0.3 to 0.42) |
| Papua New Guinea | 8035 (4781-12396) | 485.458 (288.857-748.93) | 5052 (3936-6241) | 165.106 (128.652-203.997) | 0.63 (0.45 to 0.81) |
| Paraguay | 3493 (2871-4089) | 222.893 (183.156-260.891) | 32445 (25559-41666) | 218.319 (171.983-280.372) | -0.94 (-1.1 to -0.78) |
| Peru | 29391 (24714-34907) | 331.308 (278.584-393.48) | 406464 (347140-467854) | 860.237 (734.684-990.161) | -1.14 (-1.6 to -0.68) |
| Philippines | 237118 (218650-257801) | 914.841 (843.587-994.637) | 12872 (11846-13909) | 106.392 (97.912-114.963) | 0.13 (-0.08 to 0.34) |
| Poland | 115787 (113892-117928) | 801.577 (788.459-816.396) | 2593 (2356-2896) | 87.833 (79.808-98.116) | -6.39 (-7.14 to -5.64) |
| Portugal | 8280 (7561-9056) | 218.691 (199.694-239.194) | 1633 (1382-1888) | 157.863 (133.59-182.498) | -3.73 (-4.36 to -3.1) |
| Puerto Rico | 4004 (3695-4331) | 283.074 (261.202-306.168) | 3515 (2709-4368) | 212.736 (163.936-264.318) | -2.67 (-3.21 to -2.12) |
| Qatar | 1286 (1053-1551) | 543.675 (445.214-655.807) | 25129 (17297-35441) | 224.13 (154.272-316.104) | -3.82 (-4.27 to -3.36) |
| Republic of Korea | 36575 (29245-44190) | 173.766 (138.943-209.946) | 10547 (8677-12790) | 65.914 (54.229-79.928) | -4.21 (-4.54 to -3.87) |
| Republic of Moldova | 9178 (8511-9834) | 526.6 (488.336-564.266) | 6097 (5470-6760) | 491.605 (441.007-545.028) | -0.35 (-0.73 to 0.03) |
| Romania | 43985 (40299-48235) | 506.334 (463.894-555.253) | 8397 (5718-12129) | 233.309 (158.871-336.994) | -2.35 (-2.65 to -2.05) |
| Russian Federation | 429302 (415209-440516) | 737.994 (713.767-757.271) | 15797 (14011-17778) | 293.118 (259.983-329.871) | -2.51 (-3.16 to -1.86) |
| Rwanda | 8208 (5972-11367) | 299.506 (217.921-414.772) | 223570 (209197-239610) | 481.051 (450.127-515.566) | -3.35 (-3.84 to -2.86) |
| Saint Kitts and Nevis | 74 (67-81) | 425.317 (388.313-469.975) | 8738 (5905-13165) | 154.067 (104.124-232.122) | -6.23 (-7 to -5.45) |
| Saint Lucia | 108 (99-118) | 191.645 (175.238-209.853) | 19 (13-27) | 83.831 (57.655-119.757) | -3.15 (-3.46 to -2.84) |
| Saint Vincent and the Grenadines | 172 (157-187) | 374.71 (342.077-408.135) | 57 (48-68) | 86.163 (72.302-102.594) | -2.98 (-3.22 to -2.73) |
| Samoa | 319 (218-446) | 476.304 (325.657-665.754) | 76 (65-90) | 182.814 (157.434-216.836) | 1.75 (1.62 to 1.89) |
| San Marino | 8 (6-9) | 80.167 (65.69-98.646) | 640 (446-868) | 797.038 (554.775-1080.195) | -2.69 (-2.95 to -2.43) |
| Sao Tome and Principe | 54 (36-73) | 124.633 (83.058-169.263) | 3 (2-4) | 30.41 (18.56-44.376) | 1 (0.48 to 1.52) |
| Saudi Arabia | 37028 (27500-48801) | 557.208 (413.833-734.37) | 158 (98-239) | 174.339 (107.935-263.111) | 2.06 (1.71 to 2.41) |
| Senegal | 5593 (4133-7111) | 202.699 (149.792-257.694) | 158724 (111547-220268) | 856.94 (602.234-1189.206) | -0.35 (-0.55 to -0.16) |
| Serbia | 15949 (14013-17898) | 444.12 (390.202-498.396) | 11168 (8216-15215) | 173.208 (127.422-235.973) | -3.45 (-3.57 to -3.33) |
| Seychelles | 184 (161-209) | 588.997 (516.001-669.739) | 4687 (3741-5615) | 158.122 (126.216-189.409) | -1.43 (-1.76 to -1.1) |
| Sierra Leone | 2954 (2038-4025) | 184.907 (127.562-251.96) | 143 (123-165) | 371.597 (319.323-430.573) | 1.23 (1.03 to 1.43) |
| Singapore | 5580 (5269-5942) | 369.724 (349.079-393.68) | 8796 (6065-12273) | 235.843 (162.625-329.059) | -3.16 (-3.42 to -2.9) |
| Slovakia | 11505 (10062-12981) | 562.025 (491.557-634.146) | 3116 (2914-3337) | 161.961 (151.471-173.492) | -3 (-3.25 to -2.74) |
| Slovenia | 1608 (1457-1783) | 209.777 (190.091-232.687) | 3374 (2764-4088) | 197.158 (161.494-238.898) | -5.14 (-5.47 to -4.81) |
| Solomon Islands | 1032 (561-1442) | 804.539 (437.02-1124.388) | 259 (217-305) | 45.522 (38.187-53.583) | 0.94 (0.85 to 1.03) |
| Somalia | 4998 (3430-7384) | 172.398 (118.29-254.675) | 2824 (2060-3714) | 1032.55 (753.246-1357.903) | 0.73 (0.6 to 0.86) |
| South Africa | 54086 (48588-61041) | 343.711 (308.772-387.913) | 16204 (10759-23377) | 195.454 (129.769-281.965) | -2.21 (-3.16 to -1.25) |
| South Sudan | 3573 (2479-5049) | 154.799 (107.371-218.706) | 50126 (44410-56861) | 206.73 (183.157-234.506) | 1.2 (0.72 to 1.69) |
| Spain | 32500 (30232-34974) | 219.151 (203.859-235.834) | 10909 (9897-11956) | 87.858 (79.708-96.295) | -3.19 (-3.36 to -3.02) |
| Sri Lanka | 33172 (28893-38657) | 448.591 (390.73-522.767) | 27341 (19416-36698) | 339.026 (240.759-455.052) | -1.22 (-1.56 to -0.87) |
| Sudan | 87529 (62801-118406) | 1148.593 (824.103-1553.77) | 145375 (87198-212297) | 786.425 (471.71-1148.448) | -1.18 (-1.3 to -1.07) |
| Suriname | 718 (531-846) | 440.932 (326.16-519.403) | 608 (477-768) | 283.417 (222.179-357.931) | -1.78 (-2.17 to -1.39) |
| Sweden | 3993 (3725-4275) | 136.025 (126.902-145.646) | 929 (803-1069) | 28.657 (24.774-32.984) | -4.84 (-5.07 to -4.61) |
| Switzerland | 5790 (5318-6302) | 219.747 (201.841-239.189) | 1100 (988-1224) | 39.619 (35.591-44.066) | -6.19 (-6.48 to -5.89) |
| Syrian Arab Republic | 82964 (67190-100337) | 1729.335 (1400.531-2091.469) | 47179 (35561-62401) | 927.596 (699.164-1226.886) | -1.86 (-2.15 to -1.57) |
| Taiwan (Province of China) | 14640 (13531-15811) | 158.678 (146.654-171.36) | 8908 (8033-9861) | 118.062 (106.468-130.696) | -0.31 (-0.59 to -0.03) |
| Tajikistan | 12383 (10157-14620) | 585.559 (480.329-691.375) | 15452 (11770-19417) | 370.448 (282.164-465.516) | -2.99 (-3.61 to -2.37) |
| Thailand | 65587 (48562-80627) | 252.919 (187.266-310.917) | 68789 (52710-88443) | 324.481 (248.637-417.192) | -0.85 (-1.73 to 0.04) |
| Timor-Leste | 1377 (990-1835) | 432.719 (311.1-576.488) | 2714 (1847-3841) | 475.103 (323.348-672.332) | 0.35 (-0.23 to 0.93) |
| Togo | 2334 (1821-2964) | 170.284 (132.851-216.246) | 6498 (4384-9112) | 193.118 (130.294-270.825) | 0.3 (0.04 to 0.56) |
| Tokelau | 4 (3-6) | 664.432 (442.261-966.695) | 5 (4-6) | 959.561 (795.749-1218.262) | 0.81 (0.62 to 1) |
| Tonga | 120 (94-150) | 326.075 (253.804-407.115) | 180 (131-259) | 462.514 (337.668-664.607) | 1.33 (1.12 to 1.54) |
| Trinidad and Tobago | 2325 (2159-2492) | 463.678 (430.611-497.127) | 1996 (1552-2538) | 400.961 (311.882-509.909) | -0.93 (-1.49 to -0.35) |
| Tunisia | 15430 (11773-19290) | 448.562 (342.253-560.777) | 17265 (12074-23752) | 396.725 (277.453-545.792) | -0.73 (-0.84 to -0.61) |
| Turkey | 121938 (100249-147216) | 509.8 (419.122-615.481) | 73725 (58248-90290) | 231.405 (182.828-283.398) | -2.24 (-3.09 to -1.39) |
| Turkmenistan | 11016 (9692-12231) | 717.609 (631.397-796.813) | 12440 (9549-16402) | 598.094 (459.094-788.62) | 0.56 (0.48 to 0.65) |
| Tuvalu | 38 (28-49) | 1052.385 (776.333-1353.529) | 63 (48-79) | 1265.843 (957.548-1593.431) | -2.88 (-3.22 to -2.54) |
| Uganda | 8760 (6023-12067) | 136.564 (93.9-188.121) | 33772 (23809-46570) | 196.368 (138.438-270.779) | 0.16 (-0.44 to 0.77) |
| Ukraine | 90030 (77532-105137) | 473.982 (408.185-553.516) | 97764 (71470-129207) | 709.207 (518.466-937.303) | -0.03 (-0.52 to 0.46) |
| United Arab Emirates | 4242 (3183-5628) | 443.799 (333.005-588.778) | 9887 (7185-12695) | 246.096 (178.838-315.995) | -1.98 (-2.26 to -1.69) |
| United Kingdom | 45782 (45096-46563) | 219.057 (215.771-222.79) | 18295 (17789-18940) | 84.108 (81.781-87.076) | -3.06 (-3.23 to -2.89) |
| United Republic of Tanzania | 19128 (14286-24677) | 197.419 (147.45-254.691) | 70369 (49048-94829) | 301.577 (210.203-406.404) | 1.32 (1.12 to 1.52) |
| United States of America | 241048 (236356-246427) | 235.929 (231.336-241.194) | 178952 (167807-187603) | 160.783 (150.77-168.555) | -1.42 (-1.59 to -1.26) |
| United States Virgin Islands | 199 (157-247) | 501.492 (396.2-624.633) | 93 (60-127) | 401.831 (258.757-549.544) | -0.88 (-1.23 to -0.53) |
| Uruguay | 3097 (2859-3336) | 272.626 (251.726-293.72) | 1407 (1284-1544) | 117.607 (107.368-129.085) | -2.53 (-2.97 to -2.09) |
| Uzbekistan | 56684 (50328-62971) | 660.388 (586.331-733.63) | 93353 (78067-108788) | 679.569 (568.291-791.923) | -0.13 (-0.45 to 0.18) |
| Vanuatu | 682 (471-896) | 1163.934 (803.915-1529.602) | 1783 (1311-2241) | 1430.303 (1051.805-1797.6) | 0.5 (0.41 to 0.58) |
| Venezuela (Bolivarian Republic of) | 36768 (34585-39305) | 459.077 (431.813-490.752) | 44638 (34452-57375) | 476.762 (367.967-612.806) | 0.01 (-0.47 to 0.49) |
| Viet Nam | 46437 (33372-60194) | 162.837 (117.021-211.076) | 69795 (50346-95839) | 181.805 (131.142-249.645) | 0.71 (0.35 to 1.07) |
| Yemen | 29883 (17468-46536) | 650.074 (379.992-1012.332) | 71465 (45654-108386) | 519.377 (331.796-787.707) | -0.69 (-0.83 to -0.55) |
| Zambia | 4467 (3432-5761) | 147.215 (113.116-189.879) | 16838 (11110-23568) | 208.064 (137.279-291.225) | 1.1 (0.88 to 1.32) |
| Zimbabwe | 2484 (1966-3095) | 62.66 (49.589-78.06) | 12364 (8330-17471) | 195.096 (131.435-275.668) | 4.95 (3.88 to 6.04) |

IHD, ischemic heart disease; ASR, age-standardized rate; EAPC, estimated annual percentage change; UI, uncertainty interval; CI, confidence interval.
