# Supplementary figures and images for: Global, regional, and national burden of ischemic heart disease in youths and young adults aged 15–39 years in 204 countries/territories, 1990–2021: a systematic analysis of global burden of disease study 2021
Source: Front Cardiovasc Med. 2025 Oct 14;12:1649408. doi: 10.3389/fcvm.2025.1649408 (PMC12559993; doi:10.3389/fcvm.2025.1649408)

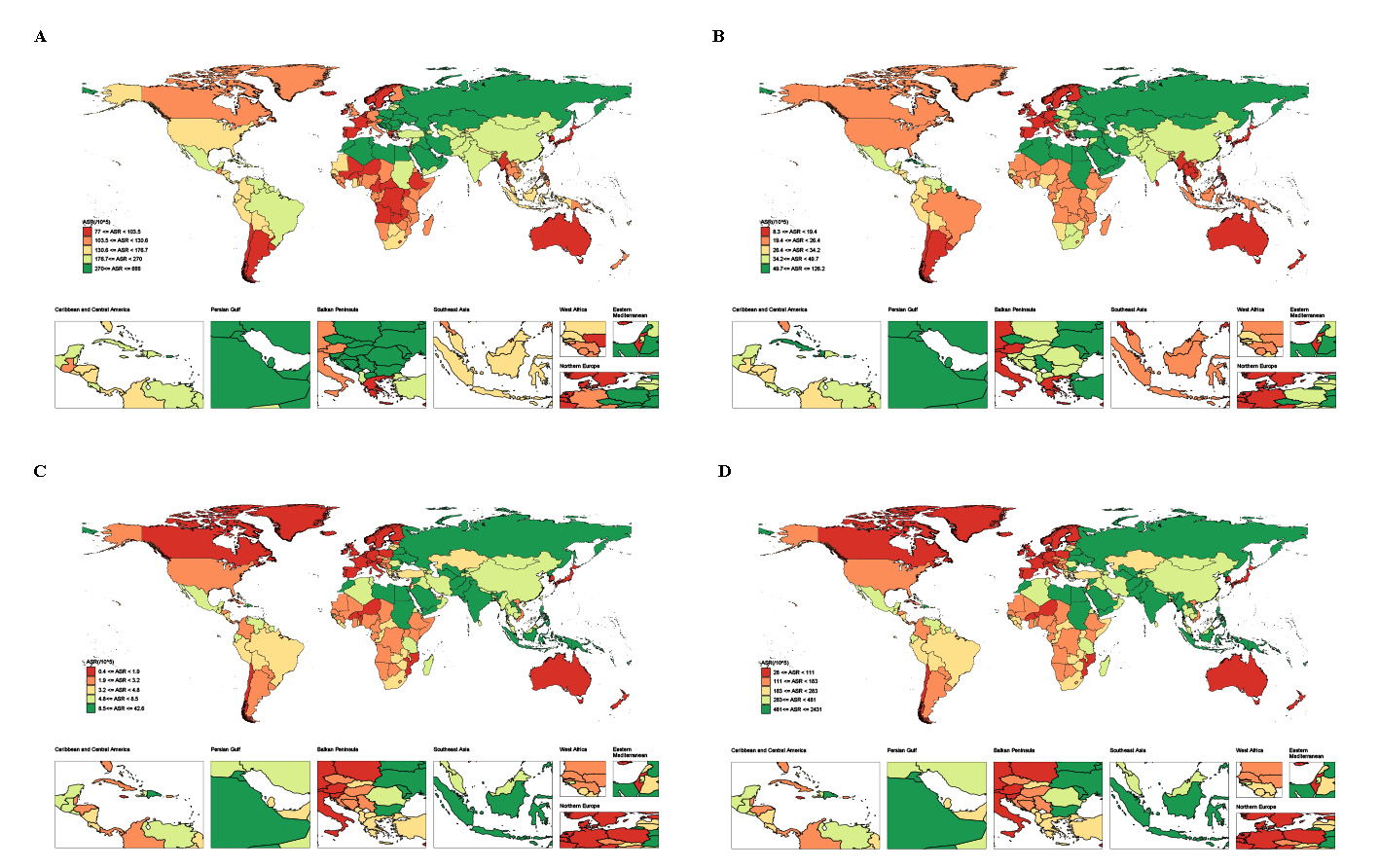

Supplement: Supplementary Figure 1 — The ASR map of IHD in 204 countries and territories. (A) Prevalence. (B) Incidence. (C) Deaths. (D) DALYs. ASR, age-standardized rate; IHD, ischemic heart disease; DALYs, disability-adjusted life years. [file Image1.jpeg]
